# Physiological and Anatomical Alterations in Children with Liver Cirrhosis

**DOI:** 10.1007/s11095-026-04119-y

**Published:** 2026-05-23

**Authors:** Femke A. Elzinga, Samira Lier, Paul R. V. Malik, Bart L. Rottier, Onno W. Akkerman, Henkjan J. Verkade, Frank Bodewes, Daan J. Touw, Paola Mian

**Affiliations:** 1 Department of Clinical Pharmacy and Pharmacology, University Medical Center Groningen, University of Groningen, Groningen, the Netherlands; 2 Pharmacometrics Expertise Center of the Northern Netherlands, University Medical Center Groningen, University of Groningen, Groningen, the Netherlands; 3 Ionis, Carlsbad, CA USA; 4 Department of Pediatric Pulmonology and Pediatric Allergology, University Medical Center Groningen, Beatrix Children’s Hospital, University of Groningen, Groningen, the Netherlands; 5 Groningen Research Institute for Asthma and COPD (GRIAC), University Medical Center Groningen, University of Groningen, Groningen, the Netherlands; 6 Department of Pulmonary Diseases and Tuberculosis, University Medical Centre Groningen, University of Groningen, Groningen, the Netherlands; 7 TB Centre Beatrixoord, University Medical Centre Groningen, University of Groningen, Groningen, the Netherlands; 8 Department of Pediatrics, Division of Pediatric Gastroenterology and Hepatology, University of Groningen, University Medical Center Groningen, Groningen, the Netherlands; 9 Department of Pharmaceutical Analysis, Groningen Research Institute for Pharmacy (GRIP), University of Groningen, Groningen, the Netherlands; 10 Department of Pediatrics, Beatrix Children’s Hospital, University Medical Center Groningen, University of Groningen, Groningen, the Netherlands; 11 Department of Pharmaceutical Technology and Biopharmacy, Groningen Research Institute for Pharmacy (GRIP), University of Groningen, Groningen, the Netherlands

**Keywords:** liver cirrhosis, pediatrics, physiologically based pharmacokinetic modeling

## Abstract

**Objective:**

To evaluate physiological and anatomical changes in children with liver cirrhosis supporting the development of physiologically based pharmacokinetic (PBPK) models.

**Methods:**

A literature review was conducted (December 2023–May 2024) using PubMed and Google Scholar to identify studies reporting physiological and anatomical parameters in children (< 18 years) with liver cirrhosis. Parameters were analyzed in relation to disease severity (Child–Pugh and/or MELD/PELD scores), stratified by age, and compared to adult data. This study examined parameters modified in adult liver cirrhosis PBPK models to assess if similar changes occur in children.

**Results:**

Parameters such as albumin, α1-acid glycoprotein, glomerular filtration rate, functional liver mass, portal blood flow, hepatic arterial blood flow, renal blood flow, and cardiac index showed either comparable alterations or lacked sufficient pediatric data to confirm differences from adult data. Hematocrit was significantly lower in children aged 2 to < 6 years (*P* = 0.022), with up to 25% greater fractional decline compared to adults, possibly due to developmental and nutritional factors.

**Conclusion:**

While children with liver cirrhosis exhibit physiological trends similar to adults, hematocrit shows a clear age-specific difference. For other parameters, limited pediatric data prevents firm conclusions, highlighting the need for age-specific studies to improve PBPK models and guide pediatric drug therapy.

**Graphical Abstract:**

Key pharmacokinetic alterations in children with liver cirrhosis compared to adults. Created with BioRender.com, incorporating data from Edginton *et al.* (2008). Abbreviations: *CP* Child–Pugh. ^=^ Parameter is similar as observed in adults. ^≠^ Parameter is different as observed in adults.

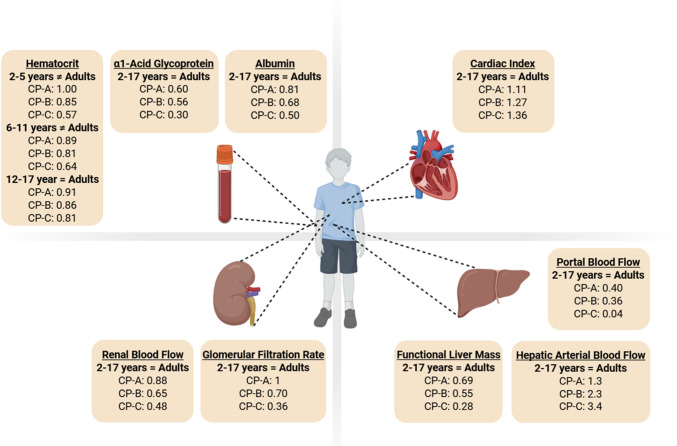

**Supplementary Information:**

The online version contains supplementary material available at 10.1007/s11095-026-04119-y.

## Introduction

Chronic liver disease is a major contributor to morbidity and mortality in children [1]. In the United States, approximately 15,000 pediatric hospitalizations annually are attributed to liver disease [2]. Progressive disease can result in liver cirrhosis, characterized by hepatocyte injury and necrosis, replacement of liver parenchyma with fibrotic tissue and regenerative nodules, and reduced hepatic function [3]. Several non-invasive markers are used to predict cirrhosis, including the aspartate aminotransferase (AST)-to-alanine aminotransferase (ALT) ratio, the AST-to-platelet ratio, and, in non-alcoholic fatty liver disease (NAFLD), the Fibrosis-4 and NAFLD fibrosis scores [4].

The etiology of liver cirrhosis differs markedly between children and adults. In adults, cirrhosis is primarily associated with hepatitis B and C, alcoholic-related liver disease, nonalcoholic steatohepatitis, and autoimmune hepatitis [5]. In contrast, pediatric cirrhosis is predominantly driven by biliary atresia and genetic or metabolic conditions, including cystic fibrosis, particularly in early childhood. With increasing age, viral hepatitis and autoimmune diseases become more prevalent causes [6]. These differences in etiology are accompanied by variation in disease progression and complications, indicating that liver cirrhosis represents a heterogenous condition across age groups. Beyond hepatic dysfunction, liver cirrhosis alters multiple physiological and anatomical processes that govern drug pharmacokinetics (PK). Drug exposure is generally increased in cirrhotic individuals, particularly for compounds undergoing hepatic metabolism. This increased exposure reflects alterations in both first-pass and systemic metabolism and depends on the metabolic pathway involved, including cytochrome P450 (CYP)-mediated oxidation and conjugation pathways such as UDP-glucuronosyltransferase. As a result, the risk of drug-induced toxicity is elevated [7]. In addition, PK of drugs differs between children and adults due to developmental changes in body composition and organ function, affecting drug and metabolite concentrations [8]. Together, disease-related and developmental changes introduce substantial variability in the PK in pediatric cirrhosis. Comprehensive characterization of absorption, distribution, metabolism and excretion (ADME) is required to establish safe and effective dosing in children. However, pediatric PK studies remain limited due to ethical and practical constraints [9]. This limitation is more pronounced in children with liver cirrhosis, as most studies evaluating hepatic impairment (HI) are conducted in adults. Consequently, pediatric dosing recommendations for HI populations are often extrapolated from adult data, as illustrated by the labeling for the triple combination therapy elexacaftor/tezacaftor/ivacaftor (marketed as Kaftrio®/Trikafta®) [10]. Importantly, PK alterations may further depend on the underlying etiology of cirrhosis, which differs not only between adults and children but also across pediatric age groups [11]. Physiologically based pharmacokinetic (PBPK) modeling provides a mechanistic approach to predict drug PK in such complex populations. These models integrate drug-specific characteristics with system-specific parameters describing organ structure, blood flow, and enzymatic processes to simulate whole-body PK [12]. By incorporating disease-related physiological changes, PBPK models can be applied to predict PK in specific populations [13, 14]. Several PBPK models have been developed for adults with liver cirrhosis [15–18]. For example, Kalam *et al.* demonstrated increased propranolol exposure with disease progression using a PBPK approach [19]. In addition, Edginton *et al.* and Johnson *et al.* defined cirrhosis-specific physiological parameter sets for adult PBPK models based on literature data [16, 17]. However, equivalent models for pediatric cirrhosis are currently lacking.

Development of pediatric cirrhosis PBPK models requires integration of age-dependent physiological changes with disease-specific alterations. Therefore, this study aimed to evaluate anatomical and physiological changes in children with liver cirrhosis to support the development of PBPK models for this population.

## Methods

### Severity Scores used within PBPK Models with Liver Cirrhosis

Liver cirrhosis severity is classified using several scoring systems. An overview of relevant definitions and scoring systems (Child–Pugh, MELD, and PELD) is provided in the Glossary. In this study, anatomical and physiological changes in pediatric liver cirrhosis were interpreted in relation to disease severity, as defined by Child–Pugh and/or MELD/PELD scores, depending on data availability. In pediatric populations, MELD/PELD scores are most commonly applied.

### Comprehensive Literature Search

A comprehensive literature search was conducted to assess changes in anatomical and physiological parameters in children with liver cirrhosis. The search focused on parameters incorporated in adult liver cirrhosis PBPK models developed by Edginton *et al.* and Johnson *et al.,* including albumin and α1-acid glycoprotein (AAG) levels, hematocrit, functional liver mass (FLM), hepatic arterial blood flow (HABF), renal blood flow (RBF), portal blood flow (PBF), glomerular filtration rate (GFR), and cardiac index (CI) [16, 17]. These parameters were selected based on their relevance in established adult PBPK models and served as a framework for evaluation in the pediatric population,

The literature search was conducted in PubMed and Google Scholar between December 2023 and May 2024 [20, 21]. Search strategies combined three key domains: (i) liver diseases (e.g., ‘liver cirrhosis’, ‘liver’, ‘hepatic’, ‘biliary’, fibrosis’); (ii) pediatric populations (e.g., ‘pediatrics’, ‘child’, ‘adolescents’); and (iii) physiological and anatomical parameters (e.g., ‘serum albumin’, ‘glomerular filtration rate’, ‘organ size’). These domains were systematically combined for each parameter of interest. Reference lists of included studies were screened to identify additional relevant publications. Detailed search terms for each parameter are provided in Supplementary Tables S1 and S2.

The expression and activity of metabolic enzymes and transporters in children with liver cirrhosis were considered outside the scope of this study. Instead, this study focuses on foundational anatomical and physiological parameters, which form the basis of PBPK models and are required prior to the incorporation of drug-specific processes such as enzyme- and transporter-mediated metabolism. Notably, even in adults with liver cirrhosis, changes in the expression and activity of major metabolic enzymes remain insufficiently characterized [22, 23].

### Inclusion and Exclusion Criteria

Studies were included if they met the following inclusion criteria: (i) conducted in children aged 2–18 years, studies including children < 2 years were considered when data for older children were unavailable; (ii) included children with HI, excluding those with major non-cirrhosis-related comorbidities that could confound anatomical and physiological parameters (e.g., malignancies, cardiac diseases); (iii) investigated the anatomical or physiological parameter of interest; and (iv) were published in English. Only studies reporting hepatic disease severity scores were included in the graphical analysis.

This study primarily focused on liver cirrhosis, while other liver diseases were considered when cirrhosis-specific data were unavailable. Liver disease was broadly defined as conditions associated with reduced hepatic function. Studies not explicitly reporting cirrhosis, but including children with HI (e.g., following liver transplantation or with chronic liver disease of various etiologies), were included only when cirrhosis-specific data were lacking. In these cases, the presence of cirrhosis could not be confirmed.

### Data Extraction and Processing

The following characteristics were extracted: country in which the study was conducted, number of subjects, ages and sex, liver disease etiology, parameter-specific values, and disease severity.

**Fig. 1:**
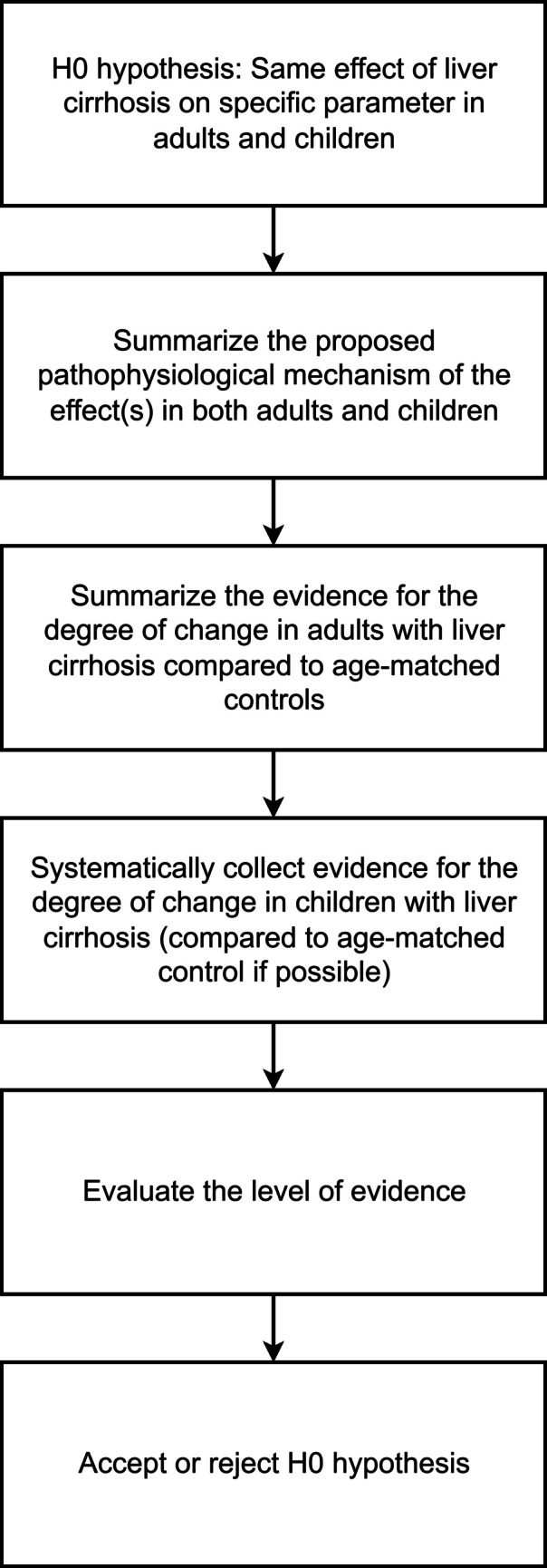
Literature-based workflow for decision-making on anatomical and physiological changes in children with liver cirrhosis.

Data processing followed the workflow outlined in Fig. 1. First, pathophysiological mechanisms underlying changes in anatomical and physiological parameters in adults with liver cirrhosis were described. Next, corresponding mechanisms in children were evaluated. The influence of age on these parameters was assessed to enable comparison between pediatric and adult populations. In addition, parameter modifications applied in adult liver cirrhosis PBPK models (Edginton *et al*. and Johnson *et al.*) and their underlying evidence base were evaluated [16, 17] (Fig. 1). The null hypothesis was that alterations in anatomical and physiological parameters are comparable between adults and children with liver cirrhosis, and substantial evidence was required to reject this hypothesis. Literature-derived data in children with liver cirrhosis were visualized for each parameter as a function of disease severity, using mean or median Child–Pugh and/or MELD/PELD scores. When data from controls were available, parameter values were normalized to age-matched controls and expressed as ratios, absolute values were also plotted. For comparison, parameter changes implemented in the adult liver cirrhosis PBPK model by Edginton *et al.* were included in the graphs [16]. Where possible, data were stratified by age groups (2 to < 6, 6 to < 12, and 12 to < 18 years), based on the reported mean or median age of each study group, corresponding to early childhood, middle childhood, and early-adolescence as defined by the National Institute of Child Health and Human Development (NICHD) [24]. To enable comparison across studies using different severity metrics, Child–Pugh scores were converted to MELD/PELD scores using an established correlation. For studies reporting Child–Pugh classes, median scores were assigned (5.5 for class A, 8 for class B, and 12.5 for class C), or mean scores were calculated when class distributions were provided. Variability was expressed using standard deviations.

After processing the literature data, parameter-specific changes in children with liver cirrhosis were characterized at the individual or mean level. When pediatric data were lacking, a null hypothesis was applied, assuming that anatomical and physiological alterations in children with liver cirrhosis are comparable to those observed in adults.

Model selection was performed by comparing linear, logarithmic, and exponential functions. Models were evaluated using the Akaike Information Criterion (AIC), in combination with visual inspection of the fit. The final model was selected based on the best balance between goodness-of-fit and physiological plausibility.

To statistically evaluate whether the relationship between disease severity (MELD/PELD) and anatomical or physiological parameters differed between pediatric age groups and adults, weighted linear regression analyses were performed. Models included an interaction term between MELD/PELD and age group, with study sample size applied as a weighting factor. Separate models were constructed for each pediatric age category compared with adults. Differences in slopes between groups were assessed based on the statistical significance of the interaction term. Two-tailed nominal *P* values ≤ 0.05 were considered statistically significant.

## Results

### Literature Search Alterations in Children with Liver Cirrhosis

A total of 55 studies were included. The number of studies informing each parameter was as follows: albumin (*n* = 11), AAG (*n* = 2), hematocrit (*n* = 6), GFR (*n* = 7), FLM (*n* = 8), PBF (*n* = 8), HABF (*n* = 3), RBF (*n* = 5), and CI (*n* = 14). Characteristics of the included studies are provided in in Supplementary Tables S3-S11.

### Relationship between Child–pugh and MELD or PELD Score

**Fig. 2:**
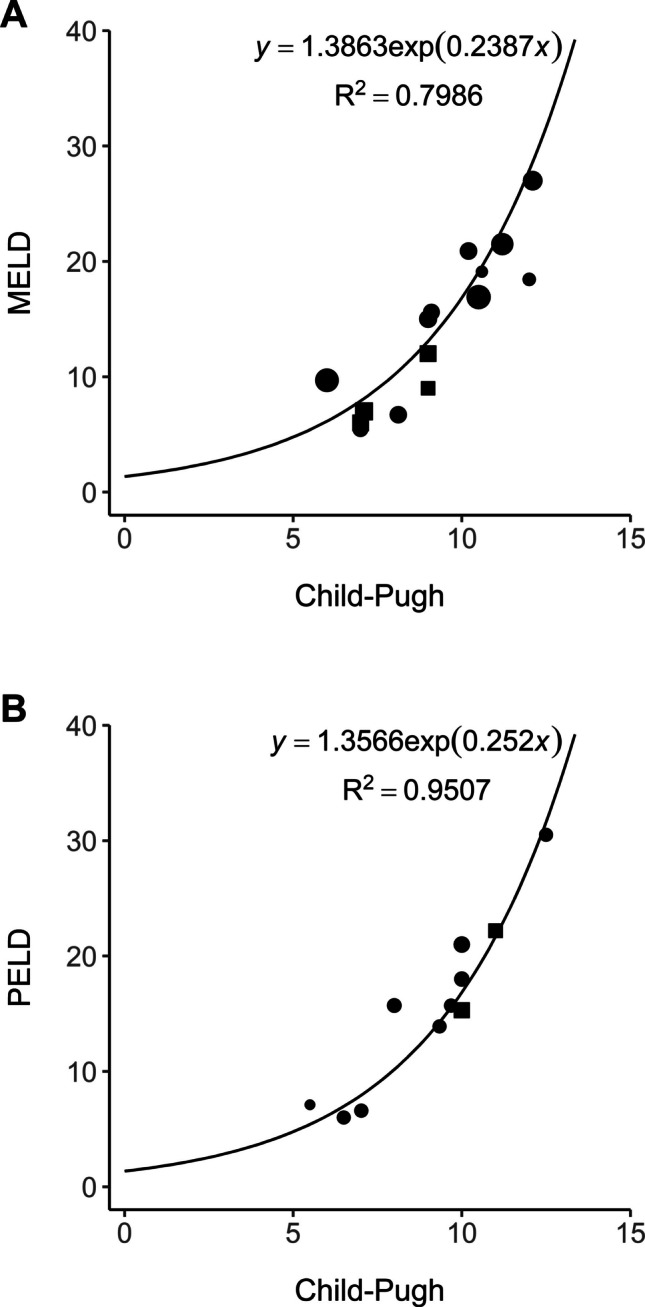
Relationship between Child–Pugh scores and (**A**) MELD and (**B**) PELD scores in children with liver disease. Point size reflects the relative number of subjects. Mean ± SD values are represented by circles, and median (IQR) values are represented by squares.

To enable comparison across studies using different severity metrics, relationships between Child–Pugh and MELD or PELD scores were established. For adults, studies reporting both Child–Pugh and MELD scores were used to define this relationship (Fig. 2A). Variation in MELD score calculation methods across studies may have introduced minor differences [25–34]. Similarly, the relationship between Child–Pugh and PELD scores was derived using studies in children reporting both metrics [35–39] (Fig. 2B). In studies reporting only PELD scores, including populations up to 18 years old, it was assumed here that PELD was used instead of MELD.

### Albumin

Albumin is the most abundant plasma protein and, due to its binding capacity, plays a crucial role in drug PK by regulating the free fraction of drugs in the blood [40]. We hypothesize that liver cirrhosis affects serum albumin levels similarly in adults and children. In adults with HI, albumin levels decrease due to reduced synthesis resulting from hepatocyte loss, dilution associated with fluid retention, and redistribution to the extravascular space driven by an increased transcapillary escape rate (TER) [41, 42]. Albumin is synthesized in hepatocytes, and renal manifestations and ascites may further contribute to altered albumin levels, a mechanism hypothesized considered similar in adults and children with HI. Although TERs has not been studied in children, the presence of portal hypertension, a major complication in pediatric cirrhosis, suggests a comparable pattern [43].

**Fig. 3:**
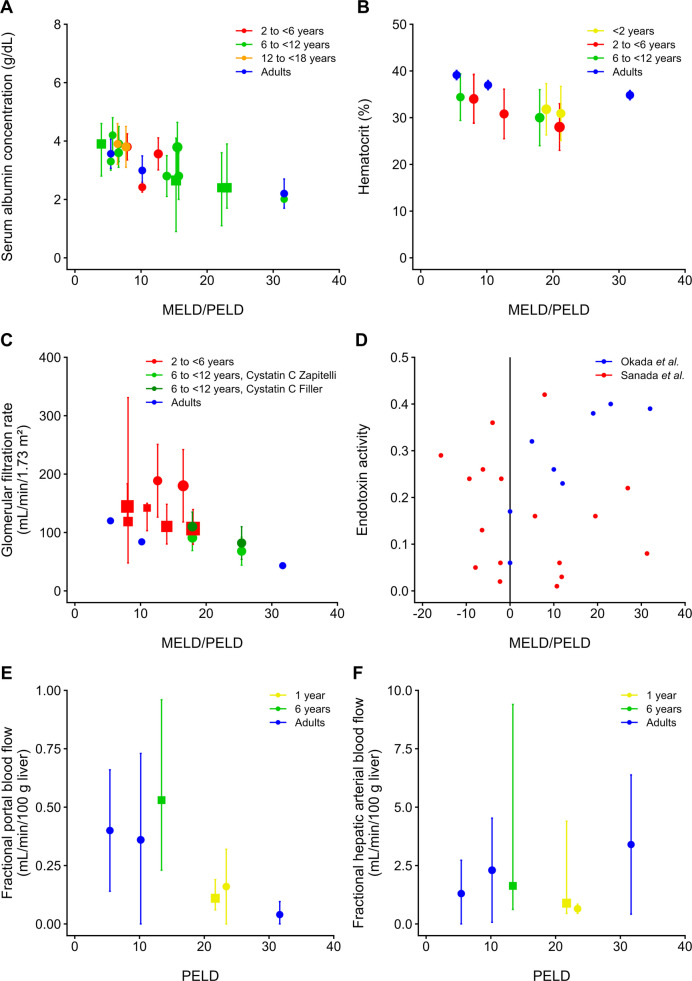

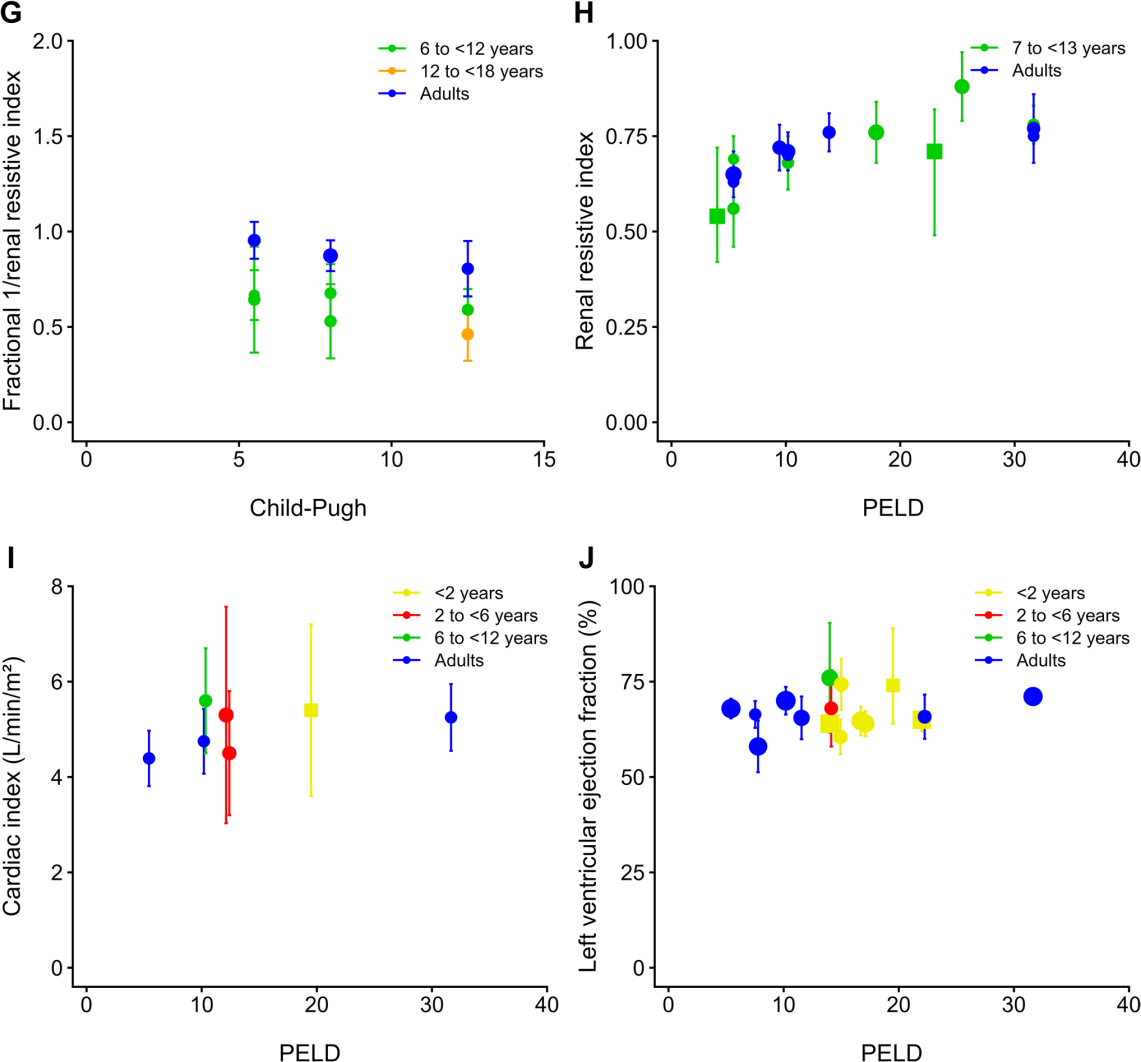
Relationship between MELD/PELD scores and physiological parameters in adults and children with liver disease. (**A**) Serum albumin concentrations, (**B**) hematocrit, (**C**) glomerular filtration, (**D**) endotoxin activity, (**E**) fractional portal blood flow, (**F**) fractional hepatic arterial blood flow, (**G**) renal blood flow (inverse of the fraction of renal resistive index), (**H**) renal resistive index, (**I**) cardiac index, and (**J**) left ventricular ejection fraction. Cirrhosis severity is classified by Child–Pugh or MELD/PELD score. Point size reflects the relative number of subjects. Error bars represent standard deviation. Mean ± SD values are represented by circles, and median (IQR) values are represented by squares.

**Fig. 4:**
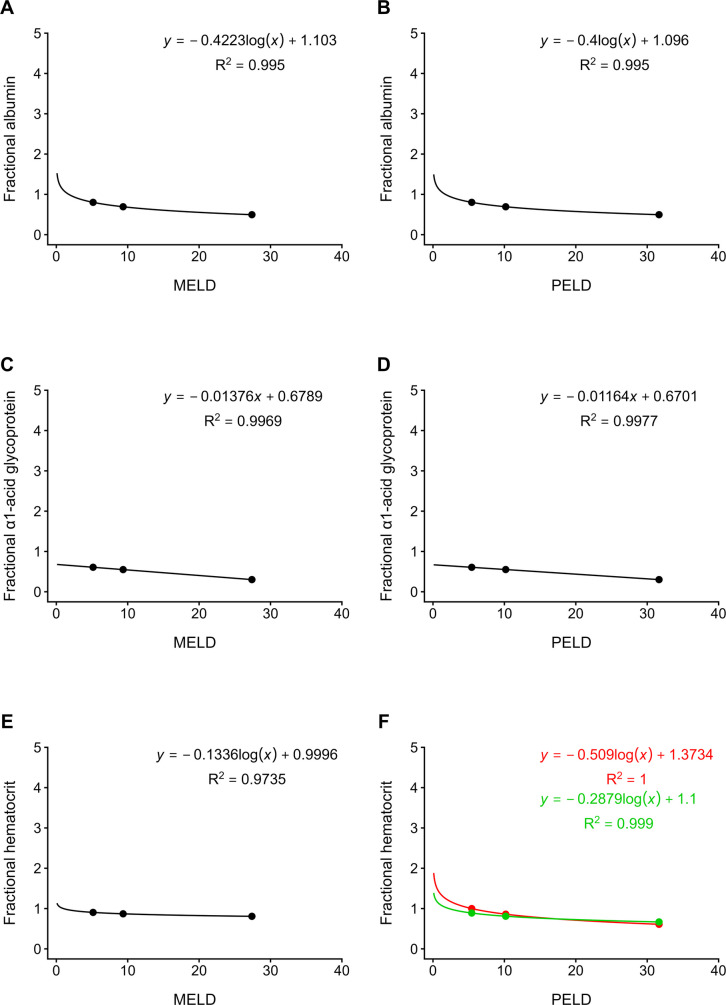

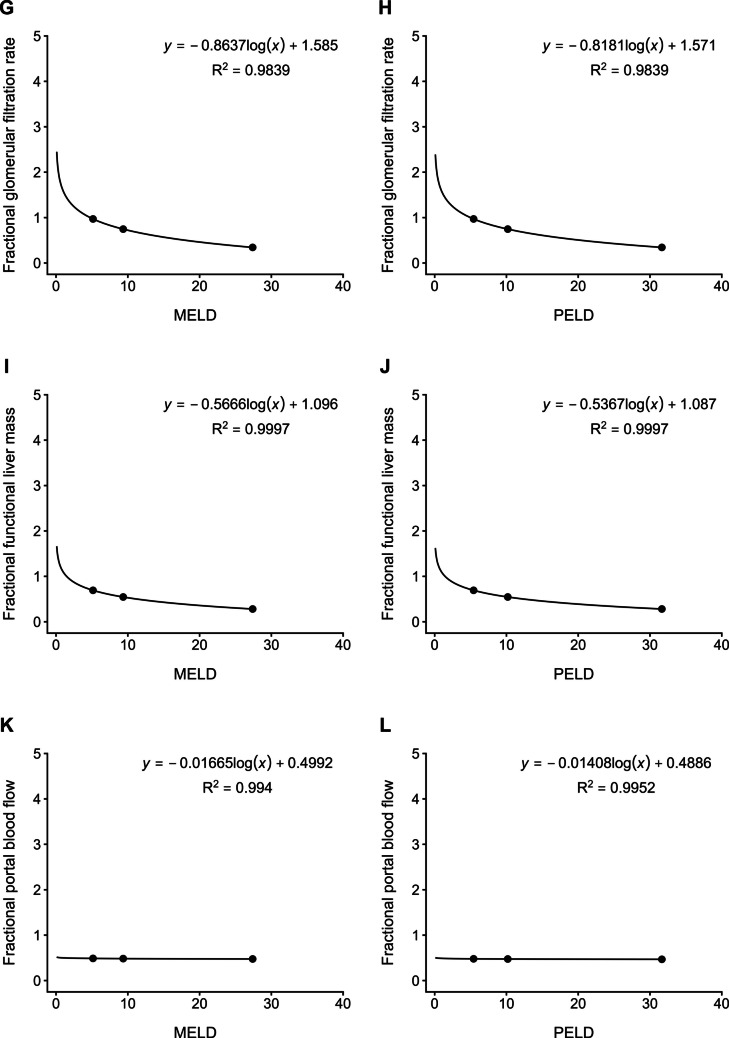

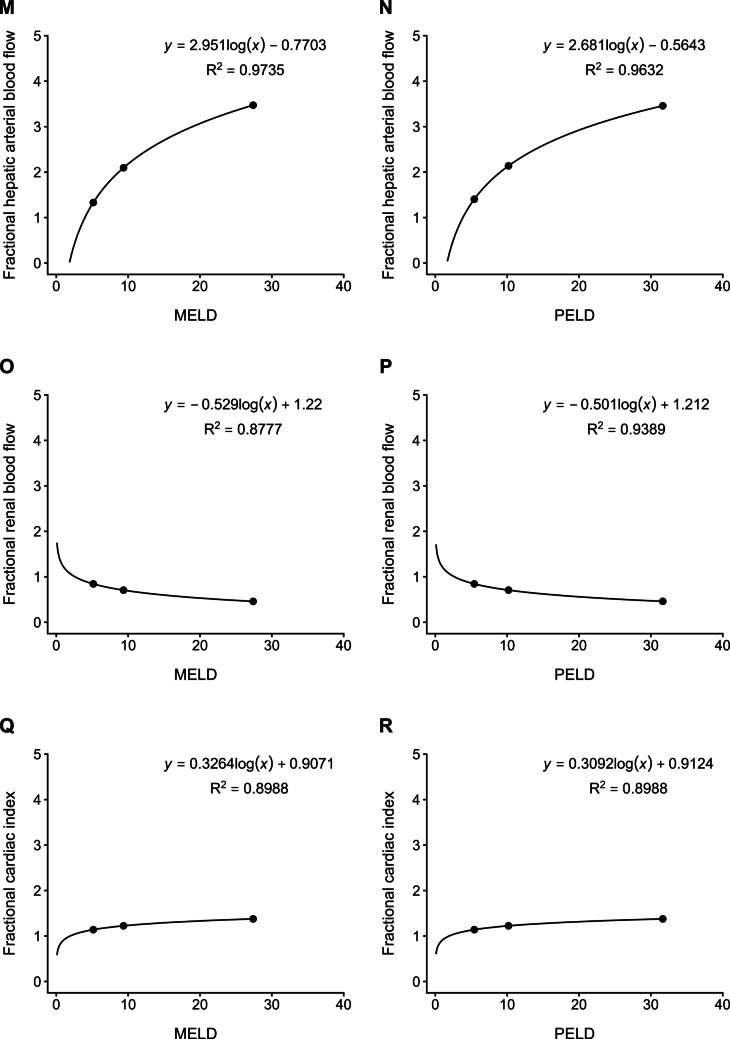
Predicted fraction of (**A, B**) serum albumin, (**C, D**) α1-acid glycoprotein, (**E, F**) hematocrit, (**G, H**) glomerular filtration rate, (**I, J**) functional liver mass, (**K, L**) portal blood flow, (**M, N**) hepatic arterial blood flow, (**O, P**) renal blood flow, and (**Q, R**) cardiac index in children with liver cirrhosis compared to healthy children according to the MELD and PELD score.

Figure 3A shows that the decline in albumin with increasing cirrhosis severity follows a similar pattern in adults and children (i.e., expressed as a fraction of age-matched controls across disease severity). No significant interaction between MELD/PELD and age group was observed (β = −0.03, *P* = 0.446), indicating similar associations between MELD/PELD and albumin levels in children and adults (Supplementary Figure S1). Using the established correlation between Child–Pugh and MELD or PELD scores, trends in albumin changes in children with liver cirrhosis were described using a logarithmic line of best fit (Fig. 4A and B). Despite variability in albumin levels across disease severity (Fig. 4A), the consistency in underlying mechanism does not support a distinct difference between adults and children [44]. Therefore, the null hypothesis cannot be rejected, indicating that albumin decreases in a comparable pattern in in adults and children with liver cirrhosis. Overall, albumin levels were estimated to decrease by 19% in Child–Pugh A, 32% in Child–Pugh B, and 50% in Child–Pugh C in children with liver cirrhosis (Table I).

**Table I:** Overview of pathophysiological parameters between adults and children with liver cirrhosis

Parameter	Adults with liver cirrhosis	Children with liver cirrhosis
Albumin	Serum albumin levels decrease due to pathophysiological mechanisms: Impaired hepatic synthesis Redistribution to the extravascular space via increased TER [42, 43]	Serum albumin levels decrease through similar mechanisms compared to adults. TER has not been studied in children, but portal hypertension suggests a comparable redistribution relative to adults [44] Decline in albumin levels [2]: Child–Pugh A: 19% Child–Pugh B: 32% Child–Pugh C: 50%
α1-Acid glycoprotein	AAG levels are unchanged or reduced, depending on the etiology and cirrhosis severity [46]	AAG levels are reduced compared to healthy children. Decline assumed to follow a similar pattern to adults [2]: Child–Pugh A: 40% Child–Pugh B: 44% Child–Pugh C: 70%
Hematocrit	Hematocrit decreases due to anemia resulting from hypersplenism, GI blood loss, nutritional deficiencies [44, 55–58]. Decline with cirrhosis severity [2]: Child–Pugh A: 9% Child–Pugh B: 14% Child–Pugh C: 19%	Lower baseline hematocrit levels due to age-related physiology. Greater susceptibility to anemia from nutritional deficiencies and growth-related factors [38, 63, 64, 71]. Steeper decline with cirrhosis severity in younger children [2]: 2–5 years: Child–Pugh A: 0% Child–Pugh B: 15% Child–Pugh C: 43% 6–11 years: Child–Pugh A: 11% Child–Pugh B: 19% Child–Pugh C: 36% 12–16 years: matches adult decline
Glomerular filtration rate	GFR decreases with cirrhosis progression due to pathophysiological mechanisms: Splanchnic vasodilatation; RAAS activation; Reduced CO; Risk of HRS [74, 75]	GFR decreases with cirrhosis progression with similar pathophysiological mechanisms compared to adults [2, 44]: Child–Pugh A: 0% Child–Pugh B: 30% Child–Pugh C: 64%
Functional liver mass	FLM decreases with cirrhosis severity due to pathophysiological mechanisms: Fibrosis; Regenerative nodules; Architectural distortion [83–87]	FLM decreases with cirrhosis severity. Decline in FLM follows a similar pattern in children and adults [2, 91, 92]: Child–Pugh A: 31% Child–Pugh B: 45% Child–Pugh C: 72%
Portal blood flow	PBF decreases with cirrhosis progression due to pathophysiological mechanisms: Sinusoidal resistance; Portosystemic shunting; Hepatocyte damage [118]	PBF decreases from intrahepatic portal venule constriction due to pathophysiological mechanisms: Hepatocyte swelling; Hyperplasia inflammation; Fibrosis [44] In advanced stages (e.g., BA) mechanism align with adults, with an estimated decline in PBF [2]: Child–Pugh A: 60% Child–Pugh B: 64% Child–Pugh C: 96%
Hepatic arterial blood flow	HABF increases as a compensatory mechanism via HABR when portal flow decreases, especially in advanced cirrhosis (Child–Pugh B/C) [18, 94, 122, 126, 127]	Portal flow is reduced; HABF is *not* proportionately increased and remains insufficient in children with BA [123, 130, 131] Predicted HABF increase [2]: Child–Pugh A: 30% Child–Pugh B: 130% Child–Pugh C: 240% *Limited data is available*
Renal blood flow	RBF decreases with cirrhosis progression due to pathophysiological mechanisms: Arterial underfilling; RAAS activation; Vasoconstriction RBF impairment correlates with increased RRI and Child–Pugh score [135–140]	RBF decreases; RRI increases with cirrhosis severity, similar to adults. Decline estimated from adult models due to lack of direct pediatric RBF studies [144–149]. Predicted reduction [2]: Child–Pugh A: 12% Child–Pugh B: 35% Child–Pugh C: 52%
Cardiac index	CI increases with cirrhosis progression due to pathophysiological mechanisms: Vasodilation; Portosystemic shunting; Low systemic resistance [154–159]	CI increases with cirrhosis severity, with similar patterns compared to adults. Hyperdynamic states may be less pronounced in younger children [166–168]. Predicted increase [2]: Child–Pugh A: 11% Child–Pugh B: 27% Child–Pugh C: 36% *Limited data is available*

Abbreviations: *AAG* α1-acid glycoprotein, *BA* biliary atresia, *CI* cardiac index, *CO* cardiac output, *FLM* functional liver mass, *GFR* glomerular filtration rate, *GI* gastrointestinal, *HABR* hepatic arterial buffer response, *HRS* hepatorenal syndrome, *PBF* portal blood flow, *RAAS* renin–angiotensin–aldosterone system, *RBF* renal blood flow, *RRI* Renal Resistive Index, *TER* transcapillary escape rate

### α1-Acid Glycoprotein

AAG is a plasma protein involved in the binding and transport of many drugs [17]. A reduced FLM in cirrhosis may impair hepatic synthetic capacity, resulting in decreased production of glycoproteins [41]. In adults with HI, AAG levels are reported to be either unchanged or reduced, depending on etiology and disease severity [45]. Several studies have shown that AAG levels in adults with cirrhosis are lower compared to age-matched controls [46–48]. As reduced FLM is also expected in children with HI, AAG levels are hypothesized to be similarly decreased. In children, baseline AAG levels may exceed those in adults, potentially due to a higher prevalence of infections, as AAG is an acute-phase protein [49, 50]. However, this study focuses on chronic liver disease rather than (sub)acute conditions. Available data indicate that AAG levels are reduced in children with cirrhosis compared to age-matched controls, although these differences are not statistically significant [51, 52]. Using the established correlation between Child–Pugh and MELD or PELD scores, trends in AAG changes in children with liver cirrhosis were described using a logarithmic line of best fit (Fig. 4C and D). Given the limited data in children and the consistency of available evidence with the null hypothesis, this hypothesis could not be rejected, indicating that AAG decreases in a similar pattern in adults and children with liver cirrhosis. Overall, AAG levels were estimated to decrease by 40% in Child–Pugh A, 44% in Child–Pugh B, and 70% in Child–Pugh C in children with liver cirrhosis (Table 1).

### Hematocrit

Hematocrit, defined as the ratio of red blood cell (RBC) volume to total blood volume, is often decreased in cirrhosis and is associated with anemia [16, 53]. Similar pathophysiological mechanisms underlying anemia are described in both children and adults. In cirrhosis, anemia results from hypersplenism, blood loss (e.g., variceal hemorrhages, ulcers), and nutritional deficiencies (e.g., iron, Vitamin B12, folate), while impaired coagulation further increases bleeding risk [43, 54–57]. As RBCs can influence drug PK, particularly for drugs with high affinity for or within RBCs, changes in hematocrit may have clinical implications [58–61]. Children have lower total blood volume and hematocrit levels than adults, which contributes to differences in anemia severity [62]. Hematocrit levels increase with age, particularly from birth to adolescence [63, 64]. Figure 3B shows a decrease in hematocrit with increasing disease severity in both adults and children with HI, with a steeper decline in younger age groups [16, 38, 65–69]. Using the established correlation between the Child–Pugh and MELD or PELD scores, hematocrit changes were described using linear trendlines, expressed as a fraction of control values (Fig. 4E and F). Age-stratified analyses indicate a more pronounced reduction in hematocrit in younger children compared to adults. This was supported by interaction analyses, which showed no significant interaction between PELD and age group for children < 2 years (β = −0.266, *P* = 0.640), and 6 to < 12 years (β = −0.233, *P* = 0.277), but a significant interaction for children aged 2 to < 6 years (β = −0.312, *P* = 0.022), indicating a more pronounced decline in this age group (Supplementary Figure S2). This may reflect increased susceptibility to disease-related factors during growth and development, including nutritional deficiencies such as iron, folate and vitamin B12, which might exacerbate anemia in liver cirrhosis [70]. Therefore, the null hypothesis is rejected, indicating that liver cirrhosis has a greater impact on hematocrit in children than in adults, particularly in those aged 2 to < 6 years. Overall, hematocrit remained unchanged in Child–Pugh A and decreased by 15% and 43% in Child–Pugh B and C, respectively, in children aged 2 to < 6 years. In children aged 6 to < 12 years, decreases of 11%, 19%, and 36% were observed for Child–Pugh A, B, and C, respectively. In adolescents (12 to < 18 years), hematocrit changes were comparable to adults, with decreases of 9%, 14%, and 19% for Child–Pugh A, B, and C, respectively (Table 1).

### Glomerular Filtration Rate

GFR is a key determinant of the PK of renally excreted drugs. A reduction in GFR decreases renal clearance and may require dose adjustments in patients with impaired renal function [71]. By the age of 2 years, GFR reaches approximately 98% of adult levels [72].

In liver cirrhosis, splanchnic vasodilation and reduced systemic vascular resistance trigger renal vasoconstriction through activation of the sympathetic nervous system and the renin–angiotensin–aldosterone system (RAAS) activation, potentially leading to hepatorenal syndrome (HRS). HRS represents a severe form of renal dysfunction, including acute kidney injury [73]. With disease progression, reduced cardiac output (CO) further aggravates renal hypoperfusion, which may result in renal failure [74]. These pathophysiological mechanisms are considered similar in adults and children [43].

GFR is commonly estimated using serum creatinine levels, however, this approach is less reliable in liver disease. Reduced hepatic function is associated with decreased muscle mass and creatine production, resulting in lower creatinine levels and overestimation of GFR [43]. Gold-standard methods involve the clearance of exogenous markers (e.g., inulin, iothalamate, iohexol), but these require invasive and labor-intensive procedures. Despite their limitations, most studies rely on creatinine-based estimations [65, 75–79]. Only one used cystatin C to estimate GFR in children with cirrhosis, providing a less invasive and potentially more reliable alternative [80]. Figure 3C shows that GFR values in children with HI are higher than in adults, which may reflect the age-related decline in GFR starting after approximately 30 years of age [80]. However, no significant interaction between MELD/PELD and age group was observed (β = −1.33, *P* = 0.821), indicating similar associations between MELD/PELD and GFR in children and adults (Supplementary Figure S3). One study reported higher GFR in children with cirrhosis compared to age matched controls, likely reflecting overestimation due to creatinine-based assessment [65].

Given the limited availability of GFR data based on exogenous markers or alterative biomarkers in children and the consistent evidence for similar pathophysiology, the null hypothesis could not be rejected. Using the established correlation between Child–Pugh and MELD or PELD scores and adult GFR trends, predicted GFR fractions in children relative to age-matched controls were derived for each severity level (Fig. 4G and H). Overall, GFR was estimated to decrease by 30% in Child–Pugh B and 64% in Child–Pugh C in children with liver cirrhosis (Table 1).

### Functional Liver Mass

The liver is the primary organ for drug metabolism, with FLM determined by both hepatocyte quantity and functional capacity [81]. In liver cirrhosis, fibrosis and regenerative nodules replace hepatocytes, resulting in reduced liver function [82–86]. Liver volume increases with body surface area (BSA) [87]. In children, the liver volume-to-body weight ratio decreases with age, leading to proportionately larger liver volumes in younger children [88]. However, liver volume alone does not reflect functional capacity, which depends on the amount of functional tissue and the degree or architectural distortion [89]. In children, cirrhosis reduces liver size and function through mechanisms similar to those observed in adults [90, 91]. In adults, FLM is assessed using techniques such as ^99m^technetium-galactosyl-neoglycoalbumin imaging [92], D-sorbitol hepatic clearance [93], radioligands [94, 95], and CT-based liver volumetry [96]. These methods have not been applied in children with cirrhosis. Therefore, this study focuses on alternative markers used in both adults and children, including endotoxin activity, β-D-Glucan (BGD), galactose elimination capacity (GEC), and albumin. Endotoxin activity reflects hepatic reserve capacity, as endotoxins accumulate in liver failure due to impaired clearance, intestinal barrier dysfunction, and microbiota alterations [97–100]. In children, findings are inconsistent: increased endotoxin activity with higher MELD/PELD scores was observed in biliary atresia prior to liver transplantation, whereas no clear trend was reported in end-stage liver disease [97, 101] (Fig. 3D). In adults, endotoxin activity increases with cirrhosis severity, particularly between Child–Pugh A and B [102].

As liver function declines, levels of BDG, a major cell wall polysaccharide found in most fungi, increases [100, 101, 103–107]. In children with biliary atresia undergoing liver transplantation, BDG clearance showed a negative correlation with PELD score, indicating worsening of liver function as cirrhosis severity increased [103]. A similar trend is observed in adults, with higher BDG levels at increased Child–Pugh scores and compared to age-matched controls [103]. GEC assesses hepatic metabolic capacity by measuring the rate of elimination of galactose by hepatocytes [108–110]. In children with chronic liver disease, prolonged galactose half-life correlates with higher MELD/PELD scores, indicating reduced hepatocyte function [108]. Comparable findings have been reported in adults [111–113]. However, as GEC reflects overall liver function rather than functional reserve specifically, its interpretability may be limited [108]. Serum albumin has also been associated with hepatic functional reserve [114]. Similar reductions in albumin levels in children and adults with cirrhosis support a comparable decline in FLM between these populations [115]. Overall, data on FLM in children with cirrhosis remain limited, and available markers provide indirect and variable estimates. However, evidence from endotoxin activity, BDG, GEC, and albumin suggests that both the magnitude and mechanisms of FLM decline are similar in children and adults. Therefore, the null hypothesis could not be rejected. Using the established correlation between Child–Pugh and MELD or PELD scores and adult FLM data, predicted FLM fractions in children relative to healthy controls were derived for each severity level (Fig. 4I and J). Overall, FLM was estimated to decrease by 31%, 45%, and 72% in Child–Pugh A, B, and C, respectively, in children with liver cirrhosis (Table 1).

### Portal Blood Flow

PBF determines drug delivery from the portal organs (e.g., gallbladder, spleen, pancreas, gastrointestinal tract) to the liver, particularly after oral absorption, and thereby influences drug PK [116]. Reduced PBF may lead to impaired hepatic perfusion and decreased drug clearance. In cirrhosis, increased intrahepatic resistance and the formation of portosystemic collateral vessels result in shunting and bypassing liver circulation, lowering PBF. In children with chronic liver diseases, this resistance primarily arises from portal venule constriction due to hepatocyte swelling, hyperplasia, inflammation, and fibrosis, rather than the sinusoidal changes observed in adults. In advanced disease stages, sinusoidal involvement becomes more prominent, resembling the adult pattern and further increasing resistance [42, 116]. Collagen deposition may contribute but is less studied. Biliary tract diseases such as biliary atresia and cystic fibrosis contribute through bile duct proliferation, portal inflammation, and fibrosis, impairing portal venules [43, 117]. In healthy children, PBF increases with age and parallels portal vein growth, maintaining relatively constant portal blood velocity [118, 119]. Consistent with this, PBPK modeling data indicate an increase in portal blood flow from infancy to adulthood [120]. In adults, dynamic contrast-enhanced MRI studies demonstrate a decrease in portal with worsening liver function, correlating with Child–Pugh class [121].

Although data in children with liver disease are limited, observed trends are generally consistent with those in adults (Fig. 3E). Reduced portal vein flow has been reported in cirrhotic children compared to non-cirrhotic controls, particularly in biliary atresia, where higher PELD scores were associated with more severe fibrosis and reduced vascular space around the portal vein and hepatic artery [122]. These findings are supported by additional studies showing greater PBF reductions in biliary atresia compared to other etiologies [122]. Younger children with biliary atresia appear to follow the decline observed in adults, whereas older children with non-biliary atresia cirrhosis show less pronounced reductions. However, no significant interaction between PELD and age group was observed (β = −0.030, *P* = 0.100) (Supplementary Figure S4). Additional evidence indicates reduced PBF in children with congenital cholestasis, although interpretation is limited by incomplete reporting of patient characteristics [122].

Overall, data on PBF in children with cirrhosis are sparse, but available evidence indicates a reduction in PBF, particularly in advanced disease stages. Therefore, the null hypothesis could not be rejected. Using established correlations between Child–Pugh and MELD/PELD scores and PBF data in cirrhotic adults, predicted portal flow fraction in cirrhotic children relative to healthy controls were derived for each severity level (Fig. 4K and L). Overall, PBF was estimated to decrease by 60%, 64%, and 96% in Child–Pugh A, B, and C, respectively, in children with liver cirrhosis (Table 1).

### Hepatic Arterial Blood Flow

The liver receives approximately 75–80% of its blood supply from the portal vein, with the remainder provided by the hepatic artery [123]. HABF contributes to hepatic drug metabolism and clearance and therefore influences drug PK [124]. We hypothesize that liver cirrhosis affects HABF similarly in adults and children.

In liver cirrhosis, particularly in advanced stages (Child–Pugh B and C), total hepatic blood flow (THBF) decreases despite partial compensation by HABF through the hepatic arterial buffer response (HABR) [17, 93, 121, 125, 126]. Chronic activation of HABR may impair its acute regulatory capacity [127]. In children with HI, HABR remains poorly characterized due to limited data [43].

In healthy children, hepatic arterial flow increases with age, although substantial variability is observed due to factors such as food intake and physiological regulation [129[(Fig. 3F). PBPK modeling data support an age-related increase in hepatic blood flow from infancy to adulthood [120]. In adults with HI, imaging studies indicate an increase in HABF with worsening liver function [16, 121]. In children with cirrhosis, portal blood flow is reduced, while the HABF does not show a compensatory increase, suggesting impaired HABR function [129]. Reduced THBF has been observed in cirrhotic children, particularly in biliary atresia, where fibrosis may further restrict vascular flow [129]. Studies consistently report limited or absent increases in HABF in cirrhotic children post-reperfusion compared to controls or donors, in contrast to observations in adults [122, 130]. Figure 3F indicates that HABF increases less in children with liver cirrhosis than in adults. However, no significant interaction between PELD and age group was observed (β = −0.16, *P* = 0.069), indicating that the association between PELD and HABF does not differ between children and adults (Supplementary Figure S5). Overall, available data suggest that HABF does not increase to the same extent in children with cirrhosis as in adults, particularly in biliary atresia. However, interpretation is limited by small sample sizes, methodological variability, confounding disease etiology, and a lack of data in older children [121, 122, 129, 130]. Additionally variability may arise from developmental differences, donor-recipient characteristics, and postprandial effects [128, 131–133]. Given these limitations, the null hypothesis could not be rejected, and no definitive conclusion can be drawn regarding differences in HABF between children and adults with cirrhosis. Using established correlations between Child–Pugh and MELD/PELD scores and adult data, predicted HABF fractions in children were derived (Fig. 4M and N**).** Overall, HABF was estimated to increase by 30%, 130%, and 240% in Child–Pugh A, B, and C, respectively, in children with liver cirrhosis (Table 1).

### Renal Blood Flow

RBF influences the drug filtration, reabsorption, and excretion, and thereby affects PK [71]. In cirrhosis, arteriolar vasodilation leads to arterial underfilling, hypotension, and activation of compensatory systems including RAAS, sympathetic nervous system, and antidiuretic hormone, resulting in sodium and water retention and potential renal dysfunction [134, 135]. Both adults and children with liver failure exhibit a hyperdynamic circulatory state characterized by vasodilatation, affecting blood volume and increasing circulatory vasodilators [134, 135]. In advanced cases, this may progress to renal vasoconstriction, reduced RBF, and decreased GFR [134, 135].

In healthy children, PBPK modeling indicates that renal blood flow increases with age, reflecting physiological development]121].

In adults with cirrhosis, RBF decreases with disease progression. Doppler ultrasound studies show that an increased renal resistive index (RRI) is associated with reduced renal perfusion and GFR [136–139]. RRI has also been shown to correlate with Child–Pugh score and age [140, 141]. Reduced RBF in cirrhosis has been further supported by imaging studies using DTPA scintigraphy [16, 142].

Although data in children are limited, available studies suggest trends similar to those observed in adults (Fig. 3G). Consistent to this, no significant interaction between Child–Pugh and age group was observed (β = 0.002, *P* = 0.892) (Supplementary Figure S6). Increased RRI correlates with higher Child–Pugh scores and is more pronounced in decompensated cirrhosis [143, 144]. Studies lacking control groups showed comparable RRI patterns when interpreted alongside adult data, supporting similar disease-related changes in renal hemodynamics [144–148] (Fig. 3H). No significant interaction between PELD and age group was observed (β = −0.011, *P* = 0.900), further indicating that these patterns do not differ between children and adults (Supplementary Figure S8).

Overall, evidence on RBF in children with cirrhosis remains limited. While RRI may reflect changes in renal perfusion, its quantitative relationship with RBF is uncertain. Available data suggests similar trends in children and adults, but interpretation is constrained by methodological differences and limited pediatric data. Therefore, the null hypothesis could not be rejected. Adult cirrhosis models were used as a reference to estimate RBF changes in children (Fig. 4O and P), although further studies are required for validation. Overall, RBF was estimated to decrease by 12%, 35%, and 52% in Child–Pugh A, B, and C, respectively, C in children with liver cirrhosis (Table 1).

### Cardiac Index

CI, defined as cardiac output (CO) normalized to BSA, reflects cardiac function and influences drug distribution, as blood flow determines drug delivery to tissues [149–152].

Cirrhosis induces a hyperdynamic circulatory state and portal hypertension characterized by increased CO, reduced vascular resistance, and hypotension, driven by vasodilation, portosystemic shunting, and bacterial translocation. This may progress to cirrhotic cardiomyopathy and cardiorenal syndrome [153–158]. In children, data on liver-related cardiac alterations are limited, although cirrhotic cardiomyopathy has been reported in approximately 20% of children with portal hypertension [159].

After the neonatal period, BSA is a reliable predictor of CO and stroke volume. CI increases during childhood, peaking around 10 years of age at 4.0 L/min/m^2^, and gradually declines thereafter to 2.4 L/min/m^2^ around the age of 80 years. Adult values range from 2.0 to 4.5 L/min/m^2^ [151, 160–163]. PBPK modeling data similarly show an age-related increase in CO from infancy (0.6 L/min) to adolescence (6 L/min) [120]. In adults with cirrhosis, CI increases with the disease severity and correlates with Child–Pugh score, reflecting the hyperdynamic state even in early stages [16, 142, 164].

Although pediatric data are limited. Available studies suggest broadly similar trends to those observed in adults (Fig. 3I). Consistent with this, no significant interaction between PELD and age was observed (β = −0.575, *P* = 0.381), indicating similar associations between children and adults (Supplementary Figure S9). Increased CI has been reported in cirrhotic children prior to liver transplantation, with reductions following transplantation, likely reflecting normalization of vascular resistance [165–167]. However, some studies report no significant difference in pre-LT CI between cirrhotic and non-cirrhotic children, suggesting that the hyperdynamic state may be less pronounced in younger children due to shorter disease duration relative to adults [122].

CI data in cirrhotic children show substantial variability but generally align with adult trends, with some higher values attributable to age-related physiological differences [142, 165, 168–170]. Measurements obtained using different techniques (e.g., PiCCO (Pulse Index Continuous Cardiac Output) and thermodilution) were comparable, although no consistent trend across disease severity was identified [142, 165, 168–170].

Left ventricular ejection fraction (LVEF), a related marker of cardiac function, does not show a consistent increase with disease severity in children with liver disease [157, 165, 171–182] (Fig. 3J). Findings are broadly comparable to adults, although age-related differences complicate direct comparison. Some studies report higher LVEF in children with liver disease compared to age-matched controls, whereas other report no significant differences [122, 177, 182].

Overall, data on CI in children with cirrhosis are limited and variable, but available evidence suggests patterns broadly comparable to adults. The underlying mechanisms of the hyperdynamic state in children remain insufficiently characterized. Therefore, the null hypothesis could not be rejected, and CI changes in children were modeled similarly to adults, showing an increase with disease severity (Fig. 4Q and R). Overall, CI was estimated to increase by 11%, 27%, and 36% in Child–Pugh A, B, and C, respectively, in children with liver cirrhosis (Table 1).

## Discussion

This study provides insights into the similarities and differences in how liver cirrhosis affects fundamental anatomy and physiology parameters in children, in the context of well-characterized effects in adults. These findings are relevant for predicting drug PK in children with liver cirrhosis, particularly for compounds lacking pediatric data, such as elexacaftor/tezacaftor/ivacaftor. Notably, up to 10% of individuals with cystic fibrosis develop liver cirrhosis within the first decade of life, emphasizing the clinical relevance of this population [183]. Understanding the impact of HI on PK is also important for other therapeutic areas, including oncology, where treatment-induced liver injury is common in pediatric patients [184]. Overall, most parameters showed trends in children comparable to adults. However, hematocrit levels in children aged 2 to < 12 years differed, which may reflect developmental factors and increased susceptibility to nutritional deficiencies.

### Limitations

This study has several limitations. First, the Child–Pugh scoring system is not designed to predict PK in children with HI. It includes subjective components (e.g., encephalopathy and ascites), does not account for etiology, and has limited discriminative power within score categories [185]. These limitations reduce the scoring system's ability to predict PK accurately, making MELD/PELD more suitable for addressing these issues and providing a more reliable assessment of liver disease severity in pediatric PBPK modeling [185]. In addition, different functional forms (linear, logarithmic, and exponential) were explored to describe relationships between disease severity and physiological parameters. Model selection was guided by statistical performance and physiological plausibility. Although linear models may theoretically yield non-physiological values outside the observed range, no negative values were obtained within the MELD/PELD range included in this study. Therefore, these models were considered appropriate for describing the observed data, but caution is warranted when extrapolating beyond this range. Additional limitations of this study include the need to incorporate liver diseases other than cirrhosis due to the limited availability of pediatric cirrhosis data. Although these conditions were not always explicitly linked to cirrhosis in the original studies, they still offer relevant insights into how impaired hepatic function influences PK. Some studies also included patients awaiting liver transplantation, a group that may not fully represent the intended PBPK modeling population. Another constraint is that several studies did not clearly define liver cirrhosis, resulting in a broad and heterogeneous interpretation of the condition. For certain physiological parameters, data availability was limited, which may affect the robustness of PK predictions. The correlations drawn between Child–Pugh and MELD/PELD scores were useful for comparing severity classifications, but they may not fully reflect true clinical relationships. We derived correlations between Child–Pugh and MELD scores, and between Child–Pugh and PELD scores, using literature-based data from adults and children with cirrhosis and an exponential function. These correlations were used to harmonize results across studies, but they rely on available, not clinically validated, relationships, introducing uncertainty into their application. Inconsistencies in how MELD and PELD scores were reported also posed challenges, as some studies referred to them collectively as "MELD/PELD" without specifying which score was used. The study did not account for potential differences related to gender or race. Furthermore, standards of care for chronic liver disease have improved over recent decades, meaning that older literature may not fully reflect current clinical practice [186, 187]. Advantages of MELD/PELD include their reliance on objective laboratory values and, in the case of MELD, the incorporation of renal function [188]. MELD and PELD provide continuous numerical measures of disease severity, whereas the Child–Pugh score is a categorical classification, which may limit its granularity [189, 190]. Despite these limitations, the compiled data still provide meaningful insights into how reduced hepatic function affects key PK parameters in children.

### Future Directions

Future research would benefit from several targeted developments that strengthen the mechanistic basis and clinical relevance of pediatric PBPK models for liver cirrhosis. A key priority is generating data on the expression of metabolic enzymes and transporters in children with cirrhosis and validating these findings across a range of drugs. At present, clinical PK data in this population are scarce, limiting the ability to rigorously evaluate PBPK model performance. Another important direction is the incorporation of mechanistic shunting models. Small *et al*. demonstrated in adults that modeling portacaval shunting improves the representation of cirrhotic physiology, and it would be valuable to investigate whether similar approaches enhance predictions in children [15]. As the clinical definition of cirrhosis has recently been formalized, emphasizing HI with portal hypertension, splenomegaly, and thrombocytopenia, further PBPK models should align with this updated framework to ensure clinical relevance [191]. Furthermore, the duration of cirrhosis remains an unexplored factor. Longitudinal studies monitoring physiological changes over time could clarify when key alterations emerge and whether disease progression differs between adults and children. Age-related differences also warrant deeper investigation. This study used NICHD Pediatric Terminology, which highlighted a steeper hematocrit decline in younger children, but alternative age stratifications may reveal different trends. Exploring multiple classification systems could help identify which physiological parameters most clearly distinguish healthy children from those with cirrhosis, and how these differ from adult patterns [24]. Scaling adult cirrhosis PBPK models to children requires integrating of age-dependent pediatric-specific pathophysiology. While this study focused on parameters previously used in adult models, such as albumin and AAG levels, hematocrit, FLM, HABF, RBF, PBF, GFR, and CI, there is a need to define additional pediatric-specific parameters [16, 17, 191]. Many of these are already used clinically to characterize cirrhosis, including total and conjugated bilirubin, bile acid, thrombocyte and leukocyte counts, AST and ALT, the gamma-glutamyl transpeptidase-to-platelet ratio index, and elastography-based measures of liver stiffness, morphology, and portal flow. Incorporating such clinical markers may strengthen the correlation between real-world practice and future pediatric PBPK models.

## Conclusion

In conclusion, this study identifies important similarities and differences in the physiological consequences of liver cirrhosis between children and adults, offering essential insights for understanding PK in pediatric cirrhosis. Although many trends in children parallel those observed in adults, certain distinctions, such as the pronounced decrease in hematocrit among children aged 2 to < 12 years, highlight the need to account for developmental physiology when evaluating disease impact. Limitations, including the inclusion of non-cirrhotic liver diseases and gaps in available pediatric data, underscore the need for further research. Nonetheless, this work represents an important first step toward addressing that gap and provides a foundation for improving PBPK modeling in pediatric liver cirrhosis. Further progress will depend on expanding age-specific data and clarifying how factors such as cirrhosis duration influence physiological changes relevant tot PK. Continued efforts in these areas will be critical for refining PBPK modes and enhancing our understanding of drug PK in children with liver cirrhosis.

## Fundings

This research did not receive any grant from funding agencies in the public, commercial, or not-for-profit sectors. Competing interests: Femke A. Elzinga, Samira Lier, Bart L. Rottier, Onno W. Akkerman, Henkjan J. Verkade, Daan J. Touw, and Paola Mian declare that they have no financial or nonfinancial interests that are directly or indirectly related to the work submitted for publication. Paul R.V. Malik is a full-time employee of Ionis Pharmaceuticals Inc. and may hold stock or stock options. Frank Bodewes employes medical consultancy activities for Vertex Pharmaceuticals Inc., outside the submitted work.

## Electronic supplementary material

Below is the link to the electronic supplementary material.

Supplementary file1 (DOCX 547 KB)

## Data Availability

Data supporting the findings of this study are available as electronic supplementary material.

## Glossary

- Liver cirrhosis: scarring of the liver due to prolonged liver damage [192].
- Hepatic impairment (HI): a condition in which the function of the liver is reduced
- Child–Pugh scoring system: liver cirrhosis can be classified according to this system. This scales disease severity based on encephalopathy score, ascites, and levels of serum bilirubin and serum albumin [193, 194].
  o Child-Pugh A: mild disease progression
  o Child-Pugh B: moderate disease progression
  o Child-Pugh C: severe disease progression
- Model for end-stage liver disease (MELD) score: originally developed to predict mortality in portal hypertension patients undergoing placement of trans jugular intrahepatic portosystemic shunts, but it is currently more used to predict survival and prioritization for liver transplantation [189, 195]. [188] Originally, the MELD score was calculated using the bilirubin level, creatinine level, INR and cause of liver disease, but it has evolved to exclude causes of disease, and a version is created which incorporates the serum sodium level and the fact if a patient is on dialysis [189].
- Pediatric end-stage liver disease (PELD) score: can be used to assess liver cirrhosis severity in children. The PELD score is established to allocate livers for transplantation in children objectively, similarly to the MELD score in adults. As with the MELD score, the PELD score is calculated using biological markers of liver function. The PELD score is restricted to children below the age of 12 [190].
