# Supplementary material for: Physiological and Anatomical Alterations in Children with Liver Cirrhosis
Source: Pharm Res. 2026 May 23;43(6):1891–914. doi: 10.1007/s11095-026-04119-y (PMC13350154; doi:10.1007/s11095-026-04119-y)

**Supplementary Materials**

**Physiological and Anatomical Alterations in Children with Liver Cirrhosis**

Femke A. Elzinga ^1,2 #^, Samira Lier ^1 #^, Paul R.V. Malik ^3^, Bart L. Rottier ^4,5^, Onno W. Akkerman ^6,7^, Henkjan J. Verkade ^8^, Frank Bodewes ^8^, Daan J. Touw ^1,2,9^, Paola Mian ^1,2,10^ *

**Affiliations**:

1. Department of Clinical Pharmacy and Pharmacology, University Medical Center Groningen and University of Groningen, Groningen, the Netherlands.
2. Pharmacometrics Expertise Center of the Northern Netherlands, University Medical Center Groningen, University of Groningen, Groningen, the Netherlands.
3. Ionis, Carlsbad, CA, USA.
4. Department of Pediatric Pulmonology and Pediatric Allergology, University Medical Center Groningen, Beatrix Children's Hospital, University of Groningen, Groningen, the Netherlands.
5. Groningen Research Institute for Asthma and COPD (GRIAC), University Medical Center Groningen, University of Groningen, Groningen, the Netherlands.
6. Department of Pulmonary Diseases and Tuberculosis, University Medical Centre Groningen, University of Groningen, Groningen, the Netherlands.
7. University Medical Centre Groningen, TB Centre Beatrixoord, University of Groningen, Groningen, the Netherlands.
8. Department of Pediatrics, Division of Pediatric Gastroenterology and Hepatology, University of Groningen, University Medical Center Groningen, Groningen, the Netherlands.
9. Department of Pharmaceutical Analysis, Groningen Research Institute for Pharmacy (GRIP), University of Groningen, Groningen, the Netherlands.
10. Department of Pediatrics, Beatrix Children’s Hospital, University Medical Center Groningen, University of Groningen, the Netherlands.

^#^ Authors contributed equally

**Supplementary Table S1** Search terms used for the literature review on physiological and anatomical parameters in children with liver cirrhosis in PubMed.

| Albumin | ("Liver Cirrhosis"[Mesh:NoExp] OR ((liver[tiab] OR hepatic[tiab] OR biliary[tiab]) AND (cirrhosis[tiab] OR fibrosis[tiab])) OR "Liver Cirrhosis, Biliary"[Mesh]) AND ("Pediatrics"[Mesh:NoExp] OR pediatri*[tiab] OR paediatric*[tiab] OR "Child"[Mesh] OR child*[tiab] OR schoolchild*[tw] OR boy[tiab] OR boys[tiab] OR boyhood[tiab] OR girl*[tiab] OR girlhood[tiab] OR youth*[tiab] OR teen*[tiab] OR puberty[tiab] OR preschool*[tiab] OR "Adolescent"[Mesh] OR adolescen*[tiab]) AND "Serum Albumin"[Mesh]  ("Liver Cirrhosis"[Mesh:NoExp] OR ((liver[tiab] OR hepatic[tiab] OR biliary[tiab]) AND (cirrhosis[tiab] OR fibrosis[tiab])) OR "Liver Cirrhosis, Biliary"[Mesh]) AND ("Pediatrics"[Mesh:NoExp] OR pediatri*[tiab] OR paediatric*[tiab] OR "Child"[Mesh] OR child*[tiab] OR schoolchild*[tw] OR boy[tiab] OR boys[tiab] OR boyhood[tiab] OR girl*[tiab] OR girlhood[tiab] OR youth*[tiab] OR teen*[tiab] OR puberty[tiab] OR preschool*[tiab] OR "Adolescent"[Mesh] OR adolescen*[tiab]) AND "Blood Proteins"[Mesh] |
| --- | --- |
| α1-Acid glycoprotein | ("Liver Cirrhosis"[Mesh:NoExp] OR ((liver[tiab] OR hepatic[tiab] OR biliary[tiab]) AND (cirrhosis[tiab] OR fibrosis[tiab])) OR "Liver Cirrhosis, Biliary"[Mesh]) AND ("Pediatrics"[Mesh:NoExp] OR pediatri*[tiab] OR paediatric*[tiab] OR "Child"[Mesh] OR child*[tiab] OR schoolchild*[tw] OR boy[tiab] OR boys[tiab] OR boyhood[tiab] OR girl*[tiab] OR girlhood[tiab] OR youth*[tiab] OR teen*[tiab] OR puberty[tiab] OR preschool*[tiab] OR "Adolescent"[Mesh] OR adolescen*[tiab]) AND "Blood Proteins"[Mesh]  ("Liver Cirrhosis"[Mesh:NoExp] OR ((liver[tiab] OR hepatic[tiab] OR biliary[tiab]) AND (cirrhosis[tiab] OR fibrosis[tiab])) OR "Liver Cirrhosis, Biliary"[Mesh]) AND ("Pediatrics"[Mesh:NoExp] OR pediatri*[tiab] OR paediatric*[tiab] OR "Child"[Mesh] OR child*[tiab] OR schoolchild*[tw] OR boy[tiab] OR boys[tiab] OR boyhood[tiab] OR girl*[tiab] OR girlhood[tiab] OR youth*[tiab] OR teen*[tiab] OR puberty[tiab] OR preschool*[tiab] OR "Adolescent"[Mesh] OR adolescen*[tiab]) AND "Acute-Phase Proteins"[Mesh]  ("Liver Cirrhosis"[Mesh:NoExp] OR ((liver[tiab] OR hepatic[tiab] OR biliary[tiab]) AND (cirrhosis[tiab] OR fibrosis[tiab])) OR "Liver Cirrhosis, Biliary"[Mesh]) AND ("Pediatrics"[Mesh:NoExp] OR pediatri*[tiab] OR paediatric*[tiab] OR "Child"[Mesh] OR child*[tiab] OR schoolchild*[tw] OR boy[tiab] OR boys[tiab] OR boyhood[tiab] OR girl*[tiab] OR girlhood[tiab] OR youth*[tiab] OR teen*[tiab] OR puberty[tiab] OR preschool*[tiab] OR "Adolescent"[Mesh] OR adolescen*[tiab]) AND "Orosomucoid"[Mesh] |
| Hematocrit | ("Liver Cirrhosis"[Mesh:NoExp] OR ((liver[tiab] OR hepatic[tiab] OR biliary[tiab]) AND (cirrhosis[tiab] OR fibrosis[tiab])) OR "Liver Cirrhosis, Biliary"[Mesh]) AND ("Pediatrics"[Mesh:NoExp] OR pediatri*[tiab] OR paediatric*[tiab] OR "Child"[Mesh] OR child*[tiab] OR schoolchild*[tw] OR boy[tiab] OR boys[tiab] OR boyhood[tiab] OR girl*[tiab] OR girlhood[tiab] OR youth*[tiab] OR teen*[tiab] OR puberty[tiab] OR preschool*[tiab] OR "Adolescent"[Mesh] OR adolescen*[tiab]) AND "Hematocrit"[Mesh] |
| Glomerular filtration rate | ("Liver Cirrhosis"[Mesh:NoExp] OR ((liver[tiab] OR hepatic[tiab] OR biliary[tiab]) AND (cirrhosis[tiab] OR fibrosis[tiab])) OR "Liver Cirrhosis, Biliary"[Mesh]) AND ("Pediatrics"[Mesh:NoExp] OR pediatri*[tiab] OR paediatric*[tiab] OR "Child"[Mesh] OR child*[tiab] OR schoolchild*[tw] OR boy[tiab] OR boys[tiab] OR boyhood[tiab] OR girl*[tiab] OR girlhood[tiab] OR youth*[tiab] OR teen*[tiab] OR puberty[tiab] OR preschool*[tiab] OR |

**Supplementary Table S1** (continued)

| Glomerular filtration rate  (continued) | "Adolescent"[Mesh] OR adolescen*[tiab]) AND "Glomerular Filtration Rate"[Mesh]  ("Liver Cirrhosis"[Mesh:NoExp] OR ((liver[tiab] OR hepatic[tiab] OR biliary[tiab]) AND (cirrhosis[tiab] OR fibrosis[tiab])) OR "Liver Cirrhosis, Biliary"[Mesh]) AND ("Pediatrics"[Mesh:NoExp] OR pediatri*[tiab] OR paediatric*[tiab] OR "Child"[Mesh] OR child*[tiab] OR schoolchild*[tw] OR boy[tiab] OR boys[tiab] OR boyhood[tiab] OR girl*[tiab] OR girlhood[tiab] OR youth*[tiab] OR teen*[tiab] OR puberty[tiab] OR preschool*[tiab] OR "Adolescent"[Mesh] OR adolescen*[tiab]) AND "Cystatin C"[Mesh]  ("Liver Cirrhosis"[Mesh:NoExp] OR ((liver[tiab] OR hepatic[tiab] OR biliary[tiab]) AND (cirrhosis[tiab] OR fibrosis[tiab])) OR "Liver Cirrhosis, Biliary"[Mesh]) AND ("Pediatrics"[Mesh:NoExp] OR pediatri*[tiab] OR paediatric*[tiab] OR "Child"[Mesh] OR child*[tiab] OR schoolchild*[tw] OR boy[tiab] OR boys[tiab] OR boyhood[tiab] OR girl*[tiab] OR girlhood[tiab] OR youth*[tiab] OR teen*[tiab] OR puberty[tiab] OR preschool*[tiab] OR "Adolescent"[Mesh] OR adolescen*[tiab]) AND "Inulin"[Mesh] |
| --- | --- |
| Functional liver mass | ("Liver Cirrhosis"[Mesh:NoExp] OR ((liver[tiab] OR hepatic[tiab] OR biliary[tiab]) AND (cirrhosis[tiab] OR fibrosis[tiab])) OR "Liver Cirrhosis, Biliary"[Mesh]) AND ("Pediatrics"[Mesh:NoExp] OR pediatri*[tiab] OR paediatric*[tiab] OR "Child"[Mesh] OR child*[tiab] OR schoolchild*[tw] OR boy[tiab] OR boys[tiab] OR boyhood[tiab] OR girl*[tiab] OR girlhood[tiab] OR youth*[tiab] OR teen*[tiab] OR puberty[tiab] OR preschool*[tiab] OR "Adolescent"[Mesh] OR adolescen*[tiab]) AND "Organ Size"[Mesh]  ("Liver Cirrhosis"[Mesh:NoExp] OR ((liver[tiab] OR hepatic[tiab] OR biliary[tiab]) AND (cirrhosis[tiab] OR fibrosis[tiab])) OR "Liver Cirrhosis, Biliary"[Mesh]) AND ("Pediatrics"[Mesh:NoExp] OR pediatri*[tiab] OR paediatric*[tiab] OR "Child"[Mesh] OR child*[tiab] OR schoolchild*[tw] OR boy[tiab] OR boys[tiab] OR boyhood[tiab] OR girl*[tiab] OR girlhood[tiab] OR youth*[tiab] OR teen*[tiab] OR puberty[tiab] OR preschool*[tiab] OR "Adolescent"[Mesh] OR adolescen*[tiab]) AND "Asialoglycoprotein Receptor"[Mesh]  ("Liver Cirrhosis"[Mesh:NoExp] OR ((liver[tiab] OR hepatic[tiab] OR biliary[tiab]) AND (cirrhosis[tiab] OR fibrosis[tiab])) OR "Liver Cirrhosis, Biliary"[Mesh]) AND ("Pediatrics"[Mesh:NoExp] OR pediatri*[tiab] OR paediatric*[tiab] OR "Child"[Mesh] OR child*[tiab] OR schoolchild*[tw] OR boy[tiab] OR boys[tiab] OR boyhood[tiab] OR girl*[tiab] OR girlhood[tiab] OR youth*[tiab] OR teen*[tiab] OR puberty[tiab] OR preschool*[tiab] OR "Adolescent"[Mesh] OR adolescen*[tiab]) AND "Radionuclide Imaging"[Mesh] |
| Portal blood flow | ("Liver Cirrhosis"[Mesh:NoExp] OR ((liver[tiab] OR hepatic[tiab] OR biliary[tiab]) AND (cirrhosis[tiab] OR fibrosis[tiab])) OR "Liver Cirrhosis, Biliary"[Mesh]) AND ("Pediatrics"[Mesh:NoExp] OR pediatri*[tiab] OR paediatric*[tiab] OR "Child"[Mesh] OR child*[tiab] OR schoolchild*[tw] OR boy[tiab] OR boys[tiab] OR boyhood[tiab] OR girl*[tiab] OR girlhood[tiab] OR youth*[tiab] OR teen*[tiab] OR puberty[tiab] OR preschool*[tiab] OR "Adolescent"[Mesh] OR adolescen*[tiab]) AND “portal blood flow”  ("Liver Cirrhosis"[Mesh:NoExp] OR ((liver[tiab] OR hepatic[tiab] OR biliary[tiab]) AND (cirrhosis[tiab] OR fibrosis[tiab])) OR "Liver Cirrhosis, Biliary"[Mesh]) AND ("Pediatrics"[Mesh:NoExp] OR pediatri*[tiab] OR paediatric*[tiab] OR "Child"[Mesh] OR child*[tiab] OR schoolchild*[tw] OR boy[tiab] OR boys[tiab] OR boyhood[tiab] OR girl*[tiab] OR girlhood[tiab] |

**Supplementary Table S1** (continued)

| Portal blood flow  (continued) | OR youth*[tiab] OR teen*[tiab] OR puberty[tiab] OR preschool*[tiab] OR "Adolescent"[Mesh] OR adolescen*[tiab]) AND "Blood Flow Velocity"[Mesh]  ("Liver Cirrhosis"[Mesh:NoExp] OR ((liver[tiab] OR hepatic[tiab] OR biliary[tiab]) AND (cirrhosis[tiab] OR fibrosis[tiab])) OR "Liver Cirrhosis, Biliary"[Mesh]) AND ("Pediatrics"[Mesh:NoExp] OR pediatri*[tiab] OR paediatric*[tiab] OR "Child"[Mesh] OR child*[tiab] OR schoolchild*[tw] OR boy[tiab] OR boys[tiab] OR boyhood[tiab] OR girl*[tiab] OR girlhood[tiab] OR youth*[tiab] OR teen*[tiab] OR puberty[tiab] OR preschool*[tiab] OR "Adolescent"[Mesh] OR adolescen*[tiab]) AND "Portal Pressure"[Mesh]  ("Liver Cirrhosis"[Mesh:NoExp] OR ((liver[tiab] OR hepatic[tiab] OR biliary[tiab]) AND (cirrhosis[tiab] OR fibrosis[tiab])) OR "Liver Cirrhosis, Biliary"[Mesh]) AND ("Pediatrics"[Mesh:NoExp] OR pediatri*[tiab] OR paediatric*[tiab] OR "Child"[Mesh] OR child*[tiab] OR schoolchild*[tw] OR boy[tiab] OR boys[tiab] OR boyhood[tiab] OR girl*[tiab] OR girlhood[tiab] OR youth*[tiab] OR teen*[tiab] OR puberty[tiab] OR preschool*[tiab] OR "Adolescent"[Mesh] OR adolescen*[tiab]) AND "Magnetic Resonance Imaging"[Mesh]  ("Liver Cirrhosis"[Mesh:NoExp] OR ((liver[tiab] OR hepatic[tiab] OR biliary[tiab]) AND (cirrhosis[tiab] OR fibrosis[tiab])) OR "Liver Cirrhosis, Biliary"[Mesh]) AND ("Pediatrics"[Mesh:NoExp] OR pediatri*[tiab] OR paediatric*[tiab] OR "Child"[Mesh] OR child*[tiab] OR schoolchild*[tw] OR boy[tiab] OR boys[tiab] OR boyhood[tiab] OR girl*[tiab] OR girlhood[tiab] OR youth*[tiab] OR teen*[tiab] OR puberty[tiab] OR preschool*[tiab] OR "Adolescent"[Mesh] OR adolescen*[tiab]) AND "Ultrasonography, Doppler"[Mesh] |
| --- | --- |
| Hepatic arterial blood flow | ("Liver Cirrhosis"[Mesh:NoExp] OR ((liver[tiab] OR hepatic[tiab] OR biliary[tiab]) AND (cirrhosis[tiab] OR fibrosis[tiab])) OR "Liver Cirrhosis, Biliary"[Mesh]) AND ("Pediatrics"[Mesh:NoExp] OR pediatri*[tiab] OR paediatric*[tiab] OR "Child"[Mesh] OR child*[tiab] OR schoolchild*[tw] OR boy[tiab] OR boys[tiab] OR boyhood[tiab] OR girl*[tiab] OR girlhood[tiab] OR youth*[tiab] OR teen*[tiab] OR puberty[tiab] OR preschool*[tiab] OR "Adolescent"[Mesh] OR adolescen*[tiab]) AND "Magnetic Resonance Imaging"[Mesh]  ("Liver Cirrhosis"[Mesh:NoExp] OR ((liver[tiab] OR hepatic[tiab] OR biliary[tiab]) AND (cirrhosis[tiab] OR fibrosis[tiab])) OR "Liver Cirrhosis, Biliary"[Mesh]) AND ("Pediatrics"[Mesh:NoExp] OR pediatri*[tiab] OR paediatric*[tiab] OR "Child"[Mesh] OR child*[tiab] OR schoolchild*[tw] OR boy[tiab] OR boys[tiab] OR boyhood[tiab] OR girl*[tiab] OR girlhood[tiab] OR youth*[tiab] OR teen*[tiab] OR puberty[tiab] OR preschool*[tiab] OR "Adolescent"[Mesh] OR adolescen*[tiab]) AND "Ultrasonography, Doppler"[Mesh]  ("Liver Cirrhosis"[Mesh:NoExp] OR ((liver[tiab] OR hepatic[tiab] OR biliary[tiab]) AND (cirrhosis[tiab] OR fibrosis[tiab])) OR "Liver Cirrhosis, Biliary"[Mesh]) AND ("Pediatrics"[Mesh:NoExp] OR pediatri*[tiab] OR paediatric*[tiab] OR "Child"[Mesh] OR child*[tiab] OR schoolchild*[tw] OR boy[tiab] OR boys[tiab] OR boyhood[tiab] OR girl*[tiab] OR girlhood[tiab] OR youth*[tiab] OR teen*[tiab] OR puberty[tiab] OR preschool*[tiab] OR "Adolescent"[Mesh] OR adolescen*[tiab]) AND "Hepatic Artery"[Mesh]  ("Liver Cirrhosis"[Mesh:NoExp] OR ((liver[tiab] OR hepatic[tiab] OR biliary[tiab]) AND (cirrhosis[tiab] OR fibrosis[tiab])) OR "Liver Cirrhosis, Biliary"[Mesh]) AND ("Pediatrics"[Mesh:NoExp] OR pediatri*[tiab] OR |

**Supplementary Table S1** (continued)

| Hepatic arterial blood flow  (continued) | paediatric*[tiab] OR "Child"[Mesh] OR child*[tiab] OR schoolchild*[tw] OR boy[tiab] OR boys[tiab] OR boyhood[tiab] OR girl*[tiab] OR girlhood[tiab] OR youth*[tiab] OR teen*[tiab] OR puberty[tiab] OR preschool*[tiab] OR "Adolescent"[Mesh] OR adolescen*[tiab]) AND "Blood Flow Velocity"[Mesh] |
| --- | --- |
| Renal blood flow | ("Liver Cirrhosis"[Mesh:NoExp] OR ((liver[tiab] OR hepatic[tiab] OR biliary[tiab]) AND (cirrhosis[tiab] OR fibrosis[tiab])) OR "Liver Cirrhosis, Biliary"[Mesh]) AND ("Pediatrics"[Mesh:NoExp] OR pediatri*[tiab] OR paediatric*[tiab] OR "Child"[Mesh] OR child*[tiab] OR schoolchild*[tw] OR boy[tiab] OR boys[tiab] OR boyhood[tiab] OR girl*[tiab] OR girlhood[tiab] OR youth*[tiab] OR teen*[tiab] OR puberty[tiab] OR preschool*[tiab] OR "Adolescent"[Mesh] OR adolescen*[tiab]) AND "Magnetic Resonance Imaging"[Mesh]  ("Liver Cirrhosis"[Mesh:NoExp] OR ((liver[tiab] OR hepatic[tiab] OR biliary[tiab]) AND (cirrhosis[tiab] OR fibrosis[tiab])) OR "Liver Cirrhosis, Biliary"[Mesh]) AND ("Pediatrics"[Mesh:NoExp] OR pediatri*[tiab] OR paediatric*[tiab] OR "Child"[Mesh] OR child*[tiab] OR schoolchild*[tw] OR boy[tiab] OR boys[tiab] OR boyhood[tiab] OR girl*[tiab] OR girlhood[tiab] OR youth*[tiab] OR teen*[tiab] OR puberty[tiab] OR preschool*[tiab] OR "Adolescent"[Mesh] OR adolescen*[tiab]) AND "Ultrasonography, Doppler"[Mesh]  ("Liver Cirrhosis"[Mesh:NoExp] OR ((liver[tiab] OR hepatic[tiab] OR biliary[tiab]) AND (cirrhosis[tiab] OR fibrosis[tiab])) OR "Liver Cirrhosis, Biliary"[Mesh]) AND ("Pediatrics"[Mesh:NoExp] OR pediatri*[tiab] OR paediatric*[tiab] OR "Child"[Mesh] OR child*[tiab] OR schoolchild*[tw] OR boy[tiab] OR boys[tiab] OR boyhood[tiab] OR girl*[tiab] OR girlhood[tiab] OR youth*[tiab] OR teen*[tiab] OR puberty[tiab] OR preschool*[tiab] OR "Adolescent"[Mesh] OR adolescen*[tiab]) AND "Blood Flow Velocity"[Mesh]  ("Liver Cirrhosis"[Mesh:NoExp] OR ((liver[tiab] OR hepatic[tiab] OR biliary[tiab]) AND (cirrhosis[tiab] OR fibrosis[tiab])) OR "Liver Cirrhosis, Biliary"[Mesh]) AND ("Pediatrics"[Mesh:NoExp] OR pediatri*[tiab] OR paediatric*[tiab] OR "Child"[Mesh] OR child*[tiab] OR schoolchild*[tw] OR boy[tiab] OR boys[tiab] OR boyhood[tiab] OR girl*[tiab] OR girlhood[tiab] OR youth*[tiab] OR teen*[tiab] OR puberty[tiab] OR preschool*[tiab] OR "Adolescent"[Mesh] OR adolescen*[tiab]) AND "Regional Blood Flow"[Mesh]  ("Liver Cirrhosis"[Mesh:NoExp] OR ((liver[tiab] OR hepatic[tiab] OR biliary[tiab]) AND (cirrhosis[tiab] OR fibrosis[tiab])) OR "Liver Cirrhosis, Biliary"[Mesh]) AND ("Pediatrics"[Mesh:NoExp] OR pediatri*[tiab] OR paediatric*[tiab] OR "Child"[Mesh] OR child*[tiab] OR schoolchild*[tw] OR boy[tiab] OR boys[tiab] OR boyhood[tiab] OR girl*[tiab] OR girlhood[tiab] OR youth*[tiab] OR teen*[tiab] OR puberty[tiab] OR preschool*[tiab] OR "Adolescent"[Mesh] OR adolescen*[tiab]) AND "Renal Blood Flow, Effective"[Mesh]  ("Liver Cirrhosis"[Mesh:NoExp] OR ((liver[tiab] OR hepatic[tiab] OR biliary[tiab]) AND (cirrhosis[tiab] OR fibrosis[tiab])) OR "Liver Cirrhosis, Biliary"[Mesh]) AND ("Pediatrics"[Mesh:NoExp] OR pediatri*[tiab] OR paediatric*[tiab] OR "Child"[Mesh] OR child*[tiab] OR schoolchild*[tw] OR boy[tiab] OR boys[tiab] OR boyhood[tiab] OR girl*[tiab] OR girlhood[tiab] OR youth*[tiab] OR teen*[tiab] OR puberty[tiab] OR preschool*[tiab] OR "Adolescent"[Mesh] OR adolescen*[tiab]) AND "Renal Circulation"[Mesh] |

**Supplementary Table S1** (continued)

| Cardiac index | ("Liver Cirrhosis"[Mesh:NoExp] OR ((liver[tiab] OR hepatic[tiab] OR biliary[tiab]) AND (cirrhosis[tiab] OR fibrosis[tiab])) OR "Liver Cirrhosis, Biliary"[Mesh]) AND ("Pediatrics"[Mesh:NoExp] OR pediatri*[tiab] OR paediatric*[tiab] OR "Child"[Mesh] OR child*[tiab] OR schoolchild*[tw] OR boy[tiab] OR boys[tiab] OR boyhood[tiab] OR girl*[tiab] OR girlhood[tiab] OR youth*[tiab] OR teen*[tiab] OR puberty[tiab] OR preschool*[tiab] OR "Adolescent"[Mesh] OR adolescen*[tiab]) AND "Cardiac Output"[Mesh] |
| --- | --- |

**Supplementary Table S2** Search terms used for the literature review on physiological and anatomical parameters in children with liver cirrhosis in Google Scholar

| Albumin | Albumin liver cirrhosis pediatrics PELD  Albumin liver cirrhosis pediatrics Child-Pugh  Albumin “chronic liver disease” children  Albumin “chronic liver disease” pediatrics  Albumin liver cirrhosis intitle:children  Albumin liver cirrhosis intitle:pediatric(s) |
| --- | --- |
| α1-Acid glycoprotein | Alpha 1 acid glycoprotein liver cirrhosis pediatrics PELD  Alpha 1 acid glycoprotein liver cirrhosis pediatrics child-pugh  Alpha 1 acid glycoprotein liver cirrhosis children  Alpha 1 acid glycoprotein liver cirrhosis children -antitrypsin  α1-acid glycoprotein liver cirrhosis pediatrics PELD  α1-acid glycoprotein liver cirrhosis pediatrics child-pugh  α1-acid glycoprotein liver cirrhosis children  α1-acid glycoprotein liver cirrhosis intitle:children  orosomucoid liver cirrhosis pediatrics PELD  orosomucoid liver cirrhosis pediatrics child-pugh  orosomucoid liver cirrhosis children  α1-acid glycoprotein “chronic liver disease” children  α1-acid glycoprotein “chronic liver disease” intitle:children  α1-acid glycoprotein “chronic liver disease” intitle:pediatric(s)  “plasma protein” liver cirrhosis pediatrics PELD  Glycoproteins liver cirrhosis children  Glycoproteins liver cirrhosis pediatrics PELD |
| Hematocrit | Hematocrit liver cirrhosis pediatrics PELD  Hematocrit liver cirrhosis intitle:children  Hematocrit liver cirrhosis intitle:pediatric(s)  Hematocrit “chronic liver disease” children child-pugh  Hematocrit “chronic liver disease” PELD  Haematocrit liver cirrhosis pediatrics PELD  Haematocrit liver cirrhosis intitle:children  Haematocrit liver cirrhosis intitle:pediatric(s)  Haematocrit “chronic liver disease” children child-pugh  Haematocrit “chronic liver disease” PELD  Anemia liver cirrhosis children  Anemia liver cirrhosis pediatrics  “red blood cells” liver cirrhosis children  “red blood cells” liver cirrhosis pediatrics |
| Glomerular filtration rate | Cystatin C gfr children liver cirrhosis  Cystatin C gfr children liver cirrhosis PELD  Inulin gfr liver cirrhosis PELD  Inulin gfr cirrhosis PELD  EDTA GFR cirrhosis PELD  Inulin clearance pediatrics cirrhosis  Inulin clearance pediatrics cirrhosis PELD  Renal function pediatrics cirrhosis  Renal function pediatrics cirrhosis PELD  Renal function GFR pediatrics PELD  GFR chronic liver disease children  GFR chronic liver disease children child-pugh  GFR chronic liver disease children PELD |

**Supplementary Table S2** (continued)

| Glomerular filtration rate  (continued) | Biomarker renal function pediatrics cirrhosis  Biomarker renal function pediatrics cirrhosis PELD  Glomerular filtration pediatrics cirrhosis PELD  Biomarker GFR pediatrics cirrhosis  GFR pediatrics cirrhosis  GFR children chronic liver disease  GFR children chronic liver disease PELD  Glomerular filtration rate pediatrics cirrhosis |
| --- | --- |
| Functional liver mass | Cirrhosis hepatic binding protein pediatrics  Cirrhosis hepatic binding protein pediatrics PELD  Cirrhosis PELD “liver function”  Cirrhosis PELD paediatrics “liver function”  Cirrhosis pediatrics “hepatic binding protein”  Cirrhosis pediatrics PELD 99m TC  Cirrhosis pediatrics PELD 99mTc -lung  Cirrhosis pediatrics PELD “radionuclide imaging”  Cirrhosis pediatrics PELD “radionuclide imaging” -lung  Cirrhosis PELD “radionuclide imaging”  Cirrhosis PELD pediatrics liver volume CT  Cirrhosis PELD pediatrics liver volume ultrasound  Cirrhosis PELD pediatric CT “liver volume”  Cirrhosis PELD pediatric CT “liver volume” -GV/SLV  Cirrhosis PELD pediatrics “liver function”  Cirrhosis PELD pediatrics “liver function” -acute  Cirrhosis PELD pediatrics “hepatic volume”  Cirrhosis PELD pediatrics “hepatic functional reserve”  Cirrhosis PELD pediatrics “functional hepatic”  Cirrhosis PELD pediatrics “hepatic function”  Cirrhosis PELD pediatrics “functional hepatic” -carcinoma  SV/SLV cirrhosis pediatrics PELD  Hepatic binding protein  Hepatic binding protein PELD children  Hepatic binding protein PELD children liver volume  Hepatic binding protein PELD children liver volume -acute  “liver volume” “chronic liver disease” PELD  “liver volume” “chronic liver disease” PELD -acute |
| Portal blood flow | Portal blood flow in children  Normal portal blood flow in children  Liver cirrhosis PELD “portal blood flow”  Liver cirrhosis hepatichemodynamics  Liver cirrhosis PELD portal vein blood flow  Liver cirrhosis PELD pediatrics “portal blood flow”  Liver cirrhosis child-pugh pediatrics “portal blood flow”  Liver cirrhosis PELD pediatrics “portal vein”  Liver cirrhosis PELD child-pugh “portal vein”  Liver cirrhosis PELD pediatrics MRI portal vein  Liver cirrhosis PELD pediatrics MRI “portal blood flow”  Liver cirrhosis PELD pediatrics Doppler “portal blood flow”  Liver cirrhosis PELD pediatrics Doppler portal hemodynamics  Liver cirrhosis PELD pediatrics Doppler portal vein  Liver cirrhosis PELD pediatrics portal vein ml/min/100  Liver cirrhosis PELD pediatrics portal ml/min/100  Liver cirrhosis PELD pediatrics “portal perfusion” |

**Supplementary Table S2** (continued)

| Hepatic arterial blood flow | Liver cirrhosis PELD “portal perfusion”  Liver cirrhosis PELD “portal perfusion” children  Liver cirrhosis PELD ml/min/100  Liver cirrhosis PELD pediatrics “portal vein flow”  Liver cirrhosis PELD portal hemodynamics  Liver cirrhosis child-pugh portal hemodynamics  Liver cirrhosis “hepatic artery blood flow” pediatrics  liver cirrhosis "hepatic arterial blood flow" pediatrics  liver cirrhosis "hepatic arterial blood flow" pediatrics -carcinoma  liver cirrhosis "hepatic artery flow" pediatrics -carcinoma  liver cirrhosis "hepatic artery perfusion" pediatrics -carcinoma  liver cirrhosis "hepatic arterial" pediatrics -carcinoma  liver cirrhosis "hepatic arterial" pediatrics doppler  liver cirrhosis "hepatic arterial" pediatrics doppler -cancer  liver cirrhosis "hepatic arterial" pediatrics doppler -cancer peld  liver cirrhosis "hepatic arterial" pediatrics doppler -cancer pugh  liver cirrhosis "hepatic arterial" pediatrics MRI -cancer  liver cirrhosis "hepatic artery" pediatrics MRI -cancer  liver cirrhosis "hepatic artery" pediatrics MRI -cancer PELD  liver cirrhosis "hepatic artery" pediatrics MRI -cancer Pugh  liver cirrhosis "hepatic artery" pediatrics doppler -cancer -thrombosis Pugh  liver cirrhosis "hepatic artery" pediatrics doppler -cancer -thrombosis Peld  liver cirrhosis "hepatic blood flow" pediatrics  liver cirrhosis "hepatic blood flow" pediatrics PELD  liver cirrhosis "hepatic blood flow" pediatrics Pugh  liver cirrhosis "hepatic blood flow" pediatrics Pugh -carcinoma  liver cirrhosis "mg/ml/100" pediatrics  liver cirrhosis "mg/ml/100" pediatrics blood flow  liver cirrhosis "ml/min/100" pediatrics blood flow  liver cirrhosis "hemodynamics" pediatrics  liver cirrhosis "hemodynamics" pediatrics PELD  liver cirrhosis " liver hemodynamics" pediatrics PELD  liver cirrhosis " liver hemodynamics" pediatrics pugh  liver cirrhosis " hepatic hemodynamics" pediatrics pugh  liver cirrhosis " hepatic hemodynamics" PELD  liver cirrhosis " hepatic arterial blood" pediatrics  liver cirrhosis " hepatic arterial blood" pediatrics -cancer  liver cirrhosis " haemodynamics" PELD  liver cirrhosis " haemodynamics" PELD artery |
| --- | --- |
| Renal blood flow | Renal doppler liver cirrhosis children  Renal resistive index liver cirrhosis  Renal resistive index liver cirrhosis child-pugh  “Renal resistive index” liver cirrhosis child-pugh  “Renal resistive index” liver cirrhosis child-pugh doppler  Renal doppler liver cirrhosis children  “renal resistive index” doppler liver cirrhosis children  “renal resistive index” doppler liver cirrhosis pediatrics  “renal resistive index" biliary atresia children  “renal resistive index" biliary atresia child-pugh  “renal resistive index" biliary atresia PELD  “Renal resistive index” liver cirrhosis children  “Renal resistive index” liver cirrhosis intitle:children  “Renal resistive index” liver cirrhosis intitle:pediatric(s)  Liver cirrhosis children renal hemodynamics |

**Supplementary Table S2** (continued)

| Renal blood flow  (continued) | Liver cirrhosis children renal hemodynamics intitle:children  Liver cirrhosis children renal hemodynamics PELD intitle:children  Liver cirrhosis children renal hemodynamics PELD intitle:pediatric(s)  Liver cirrhosis children renal hemodynamics child-pugh intitle:children  Liver cirrhosis children renal hemodynamics child-pugh intitle:pediatric(s)  Liver cirrhosis “renal hemodynamics” PELD intitle:children  Liver cirrhosis “renal hemodynamics” PELD intitle: pediatric(s)  Liver cirrhosis “renal hemodynamics” child-pugh intitle:children  Liver cirrhosis “renal hemodynamics” child-pugh intitle: pediatric(s)  Liver cirrhosis “renal blood flow” PELD  “renal blood flow index”  Liver cirrhosis “renal blood flow” PELD doppler  “renal blood flow” liver cirrhosis intitle:children  Decreased “renal blood flow” liver cirrhosis  Decreased “renal blood flow” liver cirrhosis systemic vasodilatation  Renal blood flow liver cirrhosis children  Renal blood flow cirrhosis pediatrics  Renal blood flow cirrhosis pediatrics PELD  “renal perfusion” liver cirrhosis pediatrics PELD  “arterial spin labeling” liver cirrhosis renal  “arterial spin labeling” liver cirrhosis renal inttile:children  “arterial spin labeling” liver cirrhosis renal PELD  “arterial spin labeling” liver cirrhosis renal child-pugh  “renal blood flow” liver cirrhosis scintigraphy  “renal blood flow” liver cirrhosis scintigraphy intitle:children  “renal blood flow” liver cirrhosis scintigraphy intitle:pediatric(s)  “renal blood flow” liver cirrhosis scintigraphy PELD  “renal blood flow” liver cirrhosis scintigraphy child-pugh  Tc-99m diethyltriaminopentaacetic acid liver cirrhosis renal blood flow  Tc-99m DTPA liver cirrhosis renal blood flow  Tc-99m DTPA liver cirrhosis renal blood flow intitle:children  Tc-99m DTPA liver cirrhosis renal blood flow intitle:pediatric(s)  Tc-99m DTPA liver cirrhosis renal blood flow PELD  Para-aminohippurate renal plasma flow liver cirrhosis  Para-aminohippurate renal plasma flow liver cirrhosis child-pugh  Para-aminohippurate renal plasma flow liver cirrhosis PELD  Para-aminohippurate renal flow liver cirrhosis intitle:children  Para-aminohippurate renal flow liver cirrhosis intitle:pediatric(s)  “MR perfusion imaging” liver cirrhosis  “MR perfusion imaging” liver cirrhosis intitle:children  “MR perfusion imaging” liver cirrhosis renal blood flow  MRI “renal blood flow” liver cirrhosis  MRI “renal blood flow” liver cirrhosis intitle:children  Correlation “renal resistive index” “renal blood flow”  “arterial spin labeling” “renal blood flow”  PAH clearance “renal blood flow”  Serum creatinine cirrhosis  Serum creatinine cirrhosis child-pugh  DTPA scintigraphy liver cirrhosis PELD  DTPA scintigraphy liver cirrhosis PELD renal blood flow  "renal blood flow" cirrhosis PELD |
| --- | --- |
| Cardiac index | “cardiac index” liver cirrhosis pediatrics  “cardiac index” liver cirrhosis PELD  “cardiac index” liver cirrhosis pediatrics child-pugh |

**Supplementary Table S2** (continued)

| Cardiac index  (continued) | “Cardiac index” intitle:children intitle:liver  “Cardiac index” intitle:young intitle:liver  “Cardiac index” intitle:pediatric(s) intitle:liver  “Cardiac index” "Pediatric End-Stage Liver Disease"  “left ventricular ejection fraction” liver cirrhosis pediatrics PELD  “left ventricular ejection fraction” liver cirrhosis intitle:children  “left ventricular ejection fraction” liver cirrhosis intitle:pediatric(s)  “left ventricular ejection fraction” child-pugh intitle:pediatric(s)  “left ventricular ejection fraction” child-pugh intitle:children  “left ventricular ejection fraction” child-pugh intitle:infants  “left ventricular ejection fraction” child-pugh intitle:young  “left ventricular ejection fraction” child-pugh liver cirrhosis  “left ventricular ejection fraction” intitle:children  “stroke volume” liver cirrhosis pediatrics PELD  “stroke volume” liver cirrhosis pediatrics PELD -SVV  Correlation “left ventricular ejection fraction” “cardiac index”  “cardiac output” liver cirrhosis pediatrics PELD  “cardiac output” liver cirrhosis pediatrics child-pugh  "Cardiac output" child-pugh intitle:children  "Cardiac output" child-pugh intitle:pediatric  “stroke volume index” liver cirrhosis pediatrics PELD  “stroke volume index” liver cirrhosis pediatrics child-pugh  “stroke volume index” liver cirrhosis child-pugh intitle:children  “stroke volume index” liver cirrhosis child-pugh intitle:pediatric(s)  “stroke volume index” liver cirrhosis intitle:children  “stroke volume index” liver cirrhosis intitle:pediatric(s)  “stroke volume index” liver cirrhosis intitle:children PELD  “stroke volume index” liver cirrhosis intitle:pediatric(s) PELD  “cardiac index” liver cirrhosis intitle: pediatrics  “cardiac index” liver cirrhosis intitle: pediatric -fontan -carcinoma -sepsis  "cardiac index" liver cirrhosis intitle:children  "cardiac index" liver cirrhosis intitle:children intitle:liver -fontan -carcinoma -sepsis -emergency  "cardiac index" "chronic liver disease" intitle:children  “cardiac index” child-pugh in title:children  “cardiac index” child-pugh in title:pediatric(s)  “cardiac index” PELD liver  “cardiac index” PELD transplantation  “cardiac index” chronic liver disease children  “cardiac index” chronic liver disease pediatrics  "cardiac index" chronic liver disease pediatrics -fontan -alcoholic  "cardiac index" chronic liver disease intitle:children -fontan -shock  "cardiac index" chronic liver disease intitle:pediatric -fontan -shock  "cardiac index" child-pugh pediatrics  "cardiac index" Child-pugh pediatrics cirrhosis -fontan -shock -alcoholi  “cardiac index” liver cirrhosis intitle:children  “cardiac index” “pediatric chronic liver disease”  “cardiac index” “biliary atresia”  “cardiac index” “biliary atresia” PELD  “Cardiac index” "biliary atresia" child-pugh  “cardiac index” hepatic impairment children  “cardiac index” child-pugh PELD cirrhosis  “cardiac index” liver cirrhosis pediatrics  "cardiac index" liver cirrhosis pediatrics -fontan -shock -carcinoma -prostanoid  "cardiac index" liver cirrhosis PELD |
| --- | --- |

**Supplementary Table S2** (continued)

| Cardiac index  (continued) | "cardiac index" liver disease PELD  "cardiac index" liver cirrhosis child-pugh  "cardiac index" liver cirrhosis intitle:kids  "cardiac index" liver cirrhosis intitle:youth  "cardiac index" liver cirrhosis intitle:child  “Cardiac index” liver disease intitle:young  “Cardiac index” intitle:young intitle: liver  “Cardiac index” intitle:young intitle: hepatic  “Cardiac index” intitle:children intitle:liver  “Cardiac index” intitle:pediatric intitle:liver  “Cardiac index” intitle:infants PELD  “Cardiac index” intitle:infants intitle:cirrhosis  “Cardiac index” intitle:infants intitle:liver  “Cardiac index” intitle:children intitle:liver  "Cardiac index" intitle:pediatric intitle:liver  “cardiac index” hyperdynamic circulation liver cirrhosis  “cardiac index” hyperdynamic liver cirrhosis  “Cardiac index” Pediatric End-Stage Liver Disease  “Cardiac index” "child-pugh" children |
| --- | --- |

**Supplementary Table S3** Characteristics of studies included in the literature search for plotting and additional information on albumin alterations in children with liver cirrhosis.

| **PMID/**  **DOI** | **Number of children and sex** | **Age** | **Population(s)** | **Etiology liver disease** | **Value parameter (-P value – factor change)** | **Child-Pugh score** | **Distribution Child-Pugh** | **PELD/**  **MELD score** | **Country** | **Remarks** |
| --- | --- | --- | --- | --- | --- | --- | --- | --- | --- | --- |
| 35797560 | With cirrhosis: 40 (45% male), without cirrhosis: 20 (75% male) | Mean age ± SD; with cirrhosis: 5.94 ±6.44 years, without cirrhosis: 3.58 ±3.99 years | With cirrhosis: children who underwent LT and mostly Caucasian, without cirrhosis: children without cirrhosis who underwent LT (hepatoblastoma, metabolic liver disease, genetic liver disease) | 62.5% BA, 15% autoimmune liver disease, 7.5% Alagille disease, 5% α1-acid antitrypsin deficiency, 10% other causes | Mean albumin ± SD; with cirrhosis: 3.56 ±0.55 g/dL, without cirrhosis: 4.10 ±0.54 g/dL - P0.001 - factor 87% | Mean CP±SD; with cirrhosis: 8.15 ±1.61, without cirrhosis: 5 | With cirrhosis: 12.5% A, 65% B, 22.5% C | Mean PELD/MELD±SD; with cirrhosis: 12.61 ±6.76, without cirrhosis: 0.3 ±1.34 | US | Measurement before LT |
| 23432574 | With cirrhosis: 83 (54,2% male) | Mean age ± SD; with cirrhosis: 7.2 ± 5.4 years | Children with cirrhosis awaiting LT | 33.8% cryptogenic cirrhosis, 21.8% progressive familial intrahepatic cholestasis, 15.7% BA, 10.8% Wilson's disease, 8.4% tyrosinemia, 7.2% neonatal hepatitis, 1.2% Budd-Chiari syndrome, 1.2% cardiac cirrhosis | Mean albumin ± SD; with cirrhosis: 37.9 ± 8.5 g/L | Mean CP±SD; with cirrhosis: 7.8 ± 2.1 | With cirrhosis: 28.9% A, 49.4% B, 21.7% C | Mean PELD/MELD±SD; with cirrhosis: 15.5 ± 12.5 | Iran |  |
| 35067841 | Decompensated: 51 (64.7% male), compensated: 48 (52.1% male) | Median age(range); both groups together: 11 (6-18) years | Study cohort: decompensated cirrhosis with tense ascites requiring paracentesis and albumin infusion. Controls: compensated cirrhosis. | Decompensated: 43.1% Wilson disease, 41.2% autoimmune liver disease, 2.0% chronic hepatitis B, 13.7% cryptogenic cirrhosis, compensated: 27.1% Wilson's disease, 58.3% autoimmune liver disease, 6.3% chronic hepatitis B, 8.3% | Median albumin(range); decompensated: 2.4 (1.7–3.9) g/dL, compensated: 3.9 (2.8–4.6) - P<0.001 - factor 62% | NR | NR | Median PELD (range); decompensated: 23 (11 to 39), compensated: 4 (−8 to 15) | India |  |

| **Supplementary Table S3** (continued) | | | | | | | | | | |
| --- | --- | --- | --- | --- | --- | --- | --- | --- | --- | --- |
| **PMID/**  **DOI** | **Number of children and sex** | **Age** | **Population(s)** | **Etiology liver disease** | **Value parameter (-P value – factor change)** | **Child-Pugh score** | **Distribution Child-Pugh** | **PELD/**  **MELD score** | **Country** | **Remarks** |
| 21123015 | Patients: 33 (39% male), controls: 18 (44% male) | Mean age(range); patients: 3.1 years (2 months - 11,8 years), controls: 3.2 years (3 days - 11,1 years) | Patients: pediatrics receiving MRI due to complications of BA without renal or cardiac abnormalities, controls: normal liver function, MRI for other indications | 100% BA | Mean albumin ± SD; patients: 3.8 ± 0.45 g/dL, controls 4.07 ± 0.47 g/dL - P0.0562 - factor 93% | Mean CP±SD; patients: 7 ± 1 (5 to 11), controls: 5 ± 0 | NR | NR | Taiwan |  |
| 29024698 | Survivors: 73 (68.4% male), non-survivors: 37 (64.8% male) | Median age(range); survivors: 8 years (3 months - 17 years), non-survivors: 9 years (5 months - 16 years) | Children with decompensated chronic liver disease | Both groups together: 20% autoimmune liver disease, 17.1% Wilson's disease, 16.3% Budd-Chiari syndrome, 14.5% BA, 18.1% cryptogenic cirrhosis | Median albumin(range); survivors: 2.65 g/dL (0.9-4.1), non-survivors: 2.4 g/dL (1.1-3.6) - P0.03 - factor 91% | Median CP (range); survivors: 10 (8-14), non-survivors: 11 (9-15) | NR | Median PELD (range); survivors: 15.3 (4.5-23.9), non-survivors: 22.2 (7.5-45.3) | India | Survivor vs. Non-survivor: outcome at 28 days after enroll-ment |
| 31864303 | Patients: 69 (52.2% male), controls: 50 (54%male) | Mean age ± SD (range); cases: 2.1±1.5 (0.6-5) years, controls: 2±1.5 (0.5-5.8) years | Patients: chronic liver disease, controls: healthy | 56.5% BA, 14.5% neonatal hepatitis, 10.1% progressive familial intrahepatic cholestasis, 7.3% glycogen storage disease, 5.8% Alagille disease, 5.8% congenital hepatic fibrosis | Mean albumin ± SD; patients: 3.4 ± 0.8 μmol/L, controls: 4.2 ± 0.7 μmol/L - P<0.001 - factor: 81% | NR | Patients: 24.6% A, 50.8% B, 24.6% C | NR | Egypt |  |

| **Supplementary Table S3** (continued) | | | | | | | | | | |
| --- | --- | --- | --- | --- | --- | --- | --- | --- | --- | --- |
| **PMID/**  **DOI** | **Number of children and sex** | **Age** | **Population(s)** | **Etiology liver disease** | **Value parameter (-P value – factor change)** | **Child-Pugh score** | **Distribution Child-Pugh** | **PELD/**  **MELD score** | **Country** | **Remarks** |
| 23817595 | Cases: 40 (53% male), controls: 30 (53% male), CPA: 15, CPB: 15, CPC: 10 | Mean age ± SD (range); cases: 7.6 ± 5.3 years (0.5–16), controls 7.2 ± 3 years (1–15.5) , CPA: 6.5 ± 4.5 (1–13.5) ,CPB: 5.2 ± 6.2 (0.5–15), CPC: 7.6 ± 3.0 (1–16) | Cases: cirrhosis with portal hypertension, controls: healthy | 15.0% post-hepatitis B/C, 17.5% glycogen storage disease, 17.5% BA, 15.0% Niemann Pick disease, 10% autoimmune hepatitis, 10% Budd Chiari syndrome, 8.0% Wilson's disease | Mean albumin ± SD (range); cases: 3.15 ± 0.46 g/dL (1.89–4.2), controls: 4.88 ± 0.56 g/dL (3.82–5.92) - P0.04 , CPA 3.30 ± 0.30 g/dL (2.9–4.2), CPB 2.42 ± 0.17 g/dL (2.20–2.60), CPC 2.01 ± 0.11 g/dL (1.89–2.99)- P0.032 - factors: CPA 67,6% CPB 49,6% CPC 41,2% | NR | Cases: 37.5% A, 37.5% B, 25.0% A | NR | Egypt |  |
| 27957245 | 6-12; cases:29(55% male), controls: 13 controls (61.5%). 12-18; cases: 40 (51% male) , controls: 16 (44% male) | Mean age ± SD; 6-12 years group; cases: 9.7±2.0 years, controls: 10±1,9 years. 12-18 years group; cases: 14.2±1.7 years, controls: 13.5±1,2 years | Cases: cirrhosis with esophageal varices, controls: cirrhosis without esophageal varices | Both groups together: 58,0% cryptogenic cirrhosis, 50.7% Wilson's disease, 2.9% familial intrahepatic cholestasis, 5.8% sclerosing cholangitis, 5.8% Budd-Chiari syndrome, 4.3% tyrosinemia, 4.3% glycogen storage disease, 2.9% autoimmune hepatitis, 2.9% hepatitis B infection, 2.9% Alagille disease, 1.4% α1-acid antitrypsin deficiency | Mean albumin ± SD; 6-12; cases: 3.9±0.6 mg/dL, controls: 4.2±0.6 mg/dL - P0.231 -factor=93%. 12-18; cases: 3.8±0.7 mg/dL, controls: 3.9±0.7 mg/dL - P0.757 - factor=97% | Mean CP±SD; 6-12; cases: 6.3±1,5 mg/dl, controls: 5.7±1.4. 12-18; cases: 6.9±1.9, controls 6.2±1.8 | NR | NR | Turkey | Grouped into 6-12 and 12-18 years of age |

| **Supplementary Table S3** (continued) | | | | | | | | | | |
| --- | --- | --- | --- | --- | --- | --- | --- | --- | --- | --- |
| **PMID/**  **DOI** | **Number of children and sex** | **Age** | **Population(s)** | **Etiology liver disease** | **Value parameter (-P value – factor change)** | **Child-Pugh score** | **Distribution Child-Pugh** | **PELD/**  **MELD score** | **Country** | **Remarks** |
| 24122953 | No death: 461 (43.4%male), death: 61 (54% male) | Mean age ± SD; no death: 1.7 ± 2.4 years, death: 1.2± 2.1 years | Patients with cirrhosis listed to LT | Both groups together: 72% BA, 10% α1-acid antitrypsin deficiency, 7.6% cryptogenic cirrhosis, 6.3% autoimmune hepatitis, 2% type 1 tyrosinemia, 1% biliary hypoplasia, 0.3% Wilson's disease | Mean albumin ± SD; no death: 3.0± 0,7 g/dL, death: 2.53± 0.81 g/dL -factor 84% | NR | NR | Mean PELD±SD; no death: 12.7± 9 g/dL, death: 22.7± 8,8 g/dL | Brazil | Grouped into death or no death before transplantation (death within 90 days after inclusion of patients on waiting list) |
| 22375177 | Cases: 33 (48% male), controls: 33 (48% male) | Mean age ± SD; cases: 9.4±5.4 years, controls: 9.3±5.5 years | cases: newly diagnosed chronic liver disease, controls: healthy | 63.6% metabolic liver diseases including Wilson's disease, 18.1% cholestatic liver disease, 9.1% autoimmune hepatitis, 9.1% cryptogenic cirrhosis | Mean albumin ± SD; cases: 3.6± 0,5 mg/dL, controls: 3.8± 0,4 mg/dL - P0.039 - factor 95% | NR | 66.7%A, 18.1% B, 15.2% C | Mean PELD±SD (range); cases: 6.6± 12.1 (-8 to 43) | Turkey |  |
| 34506335 | RAI: 34 (77% male), NAF: 29 (79% male) | Mean age ± SD; RAI: 11.25±3.9 years, NAF: 9.9±4.4 years | Decompensated cirrhosis: relative adrenal insufficiency (RAI) or normal adrenal function (NAF) | RAI: 35% Wilson's disease, 24% autoimmune liver disease, 24% chronic Budd-Chiari syndrome with cirrhosis, 9% chronic hepatitis B, 9% cryptogenic cirrhosis. NAF: 21% Wilson's disease, 10% autoimmune liver disease, 41% chronic Budd-Chiari syndrome with cirrhosis, 3% chronic hepatitis B, 14% cholestatic liver disease, 10% cryptogenic cirrhosis. | Mean albumin ± SD; RAI: 2.8±0.8 g/dL, NAF: 2.8±0.7 g/dL - P0.94 - factor 100% | Mean CP±SD; RAI: 9.68±2.1, NAF: 9.34±2.0 | NR | Mean PELD±SD; RAI: 15.7±14.1, NAF 13.9±12.4 | India |  |

Abbreviations: *BA* biliary atresia, *CP* Child-Pugh, *HI* hepatic impairment, *LT* liver transplantation, *NR* not reported, *SD* standard deviation.

**Supplementary Table S4** Characteristics of studies included in the literature search for plotting and additional information on α1-acid glycoprotein alterations in children with liver cirrhosis.

| **PMID/**  **DOI** | **Number of children and sex** | **Age** | **Population(s)** | **Etiology liver disease** | **Value parameter (-P value – factor change)** | **Child-Pugh score** | **Distribution Child-Pugh** | **PELD/**  **MELD score** | **Country** | **Remarks** |
| --- | --- | --- | --- | --- | --- | --- | --- | --- | --- | --- |
| [10.1111/j.1460-9592.1993.tb00028.x](https://doi.org/10.1111/j.1460-9592.1993.tb00028.x) | Liver disease: 21, controls: 28 | Mean age ± SD; liver disease: 7.6±4.2 years, controls: 6.9±4.0 years | Liver disease: children about to undergo orthotopic LT, controls: healthy children | Not reported | Mean AAG±SD; liver disease: 65.8±31.8 mg/dL, healthy: 71.8±30.7 g/dL - P>0.05 - factor 91.6% | NR | NR | NR | US |  |
| 6618555 | Patients: 20, controls: 20 | Range age; patients: 6 months to 3 years. | Patients: infants with cirrhosis, controls: children without HI | Not reported | Mean Alpha1-globulins±SE; patients: 3.90±0.17 %, controls: 3.94±0.21 % - not significantly different - factor 99% | NR | NR | NR | India |  |

Abbreviations: *HI* hepatic impairment, *LT* liver transplantation, *NR* not reported, *SD* standard deviation.

**Supplementary Table S5** Characteristics of studies included in the literature search for plotting and additional information on hematocrit alterations in children with liver cirrhosis.

| **PMID/**  **DOI** | **Number of children and sex** | **Age** | **Population(s)** | **Etiology liver disease** | **Value parameter (-P value – factor change)** | **Child-Pugh score** | **Distribution Child-Pugh** | **PELD/**  **MELD score** | **Country** | **Remarks** |
| --- | --- | --- | --- | --- | --- | --- | --- | --- | --- | --- |
| 35797560 | With cirrhosis: 40 (45% male), without cirrhosis: 20 (75% male) | Mean age ±SD; with cirrhosis: 5.94 ±6.44 years, without cirrhosis: 3.58 ±3.99 years | With cirrhosis: children who underwent LT and mostly Caucasian, without cirrhosis: children without cirrhosis who underwent LT (hepatoblastoma, metabolic liver disease, genetic liver disease) | 62.5% BA, 15% autoimmune liver disease, 7.5% Alagille disease, 5% α1-acid antitrypsin deficiency, 10% other causes | Mean HTC ± SD; with cirrhosis: 30.8±5,31%, without cirrhosis: 32.46±4.82 - P0.26 (not significantly different) - factor 95% | Mean CP±SD; with cirrhosis: 8.15 ±1.61, without cirrhosis: 5 | With cirrhosis: 12.5% A, 65% B, 22.5% C | Mean PELD/MELD±SD; with cirrhosis: 12.61 ±6.76, without cirrhosis: 0.3 ±1.34 | US |  |
| 20339881 | DDLT:56 DDLT (62.5% male), LDLT: 101 (47.5% male) | Mean age ± SD; DDLT: 9.5±6.1 years, LDLT 4.6±4.8 years | Children who underwent orthotopic LT for end-stage liver disease | DDLT: 14% BA, 16% fulminant hepatic failure, 16% Wilson's disease, 13% congenital hepatic fibrosis, 7% cryptogenic cirrhosis, 9% familial intrahepatic cholestasis, 9% autoimmune hepatitis, 5% hepatitis B, 2% glycogen storage disease, 9% others. LDLT: 39% BA, 12% fulminant hepatic failure, 5% Wilson's disease, 9% tyrosinemia, 2% congenital hepatic fibrosis, 6% cryptogenic cirrhosis, 6% familial intrahepatic cholestasis, 4% autoimmune hepatitis, 3% hepatitis B, 3% glycogen storage disease, 12% others. | Mean HTC±SD; DDLT: 30±6 %, LDLT: 28±5 % | Mean CP±SD; DDLT: 10±3, LDLT 10±2 | NR | Mean PELD±SD; DDLT: 18±13, LDLT: 21±12 | Turkey | Grouped into deceased donor liver transplantation (DDLT) and living donor liver transplantation (LDLT), preoperative HTC value |

| **Supplementary Table S5** (continued) | | | | | | | | | | |
| --- | --- | --- | --- | --- | --- | --- | --- | --- | --- | --- |
| **PMID/**  **DOI** | **Number of children and sex** | **Age** | **Population(s)** | **Etiology liver disease** | **Value parameter (-P value – factor change)** | **Child-Pugh score** | **Distribution Child-Pugh** | **PELD/**  **MELD score** | **Country** | **Remarks** |
| 30577227 | 24 (33.3% male) | Mean age ± SD: 8.0±5.2 years | Cirrhosis with intrapulmonary vascular shunting undergoing LT | 70.8% BA, 16.6% congenital hepatic fibrosis, 4.0% Caroli's disease, 4.0% autoimmune hepatitis, 4.0% idiopathic copper toxicosis | Mean HTC±SD: 34.4±5.0 % | Median CP (range): 6.5 (5-10) | NR | Median PELD/MELD (range): 6 (0-25) | Japan |  |
| 23142314 | Cirrhosis: 39 (48.7% male), controls: (48.7% male) | Median age (25th - 75th percentiles); cirrhotic: 2.5 (0.7–9.6), controls: 2.6 (1.3–9.5) | Cirrhosis: diagnosis of cirrhosis established histologically, controls: healthy controls matched by sex and age | Cirrhosis: 66.7% BA, 2.6% choledochal cyst, 7.7% autoimmune hepatitis, 5.1 α1-acid antitrypsin deficiency, 17.9% cryptogenic | Mean HTC±SD; cirrhosis: 34.1±5.2%, controls: 36.8±2.75% -P=0.008 -Factor 92,7% | NR | Cirrhosis: 51.3% A, 41.0%B, 7.7% C | NR | Brazil |  |
| 27382957 | Cirrhosis: 53 (34% male), control: 33 (39.4% male) | Median age (25th-75th percentiles); cirrhosis: 1.97 (0.7-7.3) years, controls: 2.4 (1.3-7.5) years | Cirrhosis: cirrhosis due to BA, controls: healthy, matched by gender and age | 100% BA | Mean HTC±SD; cirrhosis: 34.0 ± 5.2% - controls: higher HTC | NR | Cirrhosis: 5.5% A, 30.2% B, 11.3% C | NR | Brazil | Age cirrhosis rounded to 2, HTC strong correlation with Child-Pugh score |
| 34494348 | Non-neutropenia: 61 (49.2% male), neutropenia: 39 (46.2% male) | Median age (IQR); non-neutropenia: 1.42 (0.83, 9.17) years, neutropenia: 1.5 (0.67, 11.42) years | Neutropenia: children with neutropenia after LT, non-neutropenia: children without neutropenia after LT | Both groups together: 41% BA, 18% metabolic liver disease, 15% liver tumor disease, 10% genetic liver disease, 8% autoimmune liver disease, 3% acute liver failure, 5% other causes | Mean HTC±SD; non-neutropenia: 31.8±5.5 %, neutropenia: 30.9±5.8 % - P0.45 | NR | NR | Mean PELD±SD; non-neutropenia: 19, neutropenia: 21,2 | US | Preoperative HTC value |

Abbreviations: *BA* biliary atresia, *CP* Child-Pugh, *HI* hepatic impairment, *HTC* hematocrit, *IQR* interquartile range, *LT* liver transplantation, *NR* not reported, *SD* standard deviation.

**Supplementary Table S6** Characteristics of studies included in the literature search for plotting and additional information on alterations in glomerular filtration rate in children with liver cirrhosis.

| **PMID/**  **DOI** | **Number of children and sex** | **Age** | **Population(s)** | **Etiology liver disease** | **Value parameter (-P value – factor change)** | **Child-Pugh score** | **Distribution Child-Pugh** | **PELD/**  **MELD score** | **Country** | **Remarks** |
| --- | --- | --- | --- | --- | --- | --- | --- | --- | --- | --- |
| 35797560 | With cirrhosis: 40 (45% male), without cirrhosis: 20 (75% male) | Mean age ± SD; with cirrhosis: 5.94 ±6.44 years, without cirrhosis: 3.58 ±3.99 years | With cirrhosis: children who underwent LT and mostly Caucasian, without cirrhosis: children without cirrhosis who underwent LT (hepatoblastoma, metabolic liver disease, genetic liver disease) | 62.5% BA, 15% autoimmune liver disease, 7.5% Alagille disease, 5% α1-acid antitrypsin deficiency, 10% other causes | Mean GFR±SD; with cirrhosis: 188.6±62.34 ml/min/1.73m2, without cirrhosis: 145.94±40.58 ml ml/min/1.73m2 - P0.002 - factor 129% | Mean CP±SD; with cirrhosis: 8.15 ±1.61, without cirrhosis: 5 | With cirrhosis: 12.5% A, 65% B, 22.5% C | Mean PELD/MELD±SD; with cirrhosis: 12.61 ±6.76, without cirrhosis: 0.3 ±1.34 | US | Assumed creatinine was used for GFR, data from electronic chart of patient |
| 33978827 | AKI: 41 (68% male), non-AKI: 59 (50.8% male) | Mean age ± SD; AKI: 9.2±5.3 years, non-AKI: 7.7±5.4 years | children with diagnosis of chronic liver disease with portal hypertension | AKI: 27% autoimmune hepatitis, 24% Wilson's disease, 22% BA, 10% progressive familial intrahepatic cholestasis, 5% hepatitis B, 5% primary sclerosing cholangitis, 5% Alagille syndrome | Mean GFR±SD; AKI: Schwartz equation creatinine based 167±108 ml/min/1.73m2, Zapitelli equation cystatin C based 68±24 ml/min/1.73m2, filler equation cystatin C based 82±28 ml/min/1.73m2, Bouvet equation cystatin C + creatinine based 108±69 ml/min/1.73m2. Non-AKI: Schwartz equation creatinine based 189±98 ml/min/1.73m2, Zapitelli equation cystatin C based 91±22 ml/min/1.73m2, filler equation cystatin C based 110±25 ml/min/1.73m2, Bouvet equation cystatin C + creatinine based 129±49 ml/min/1.73m2 | NR | NR | Mean PELD±SD; AKI: 25.4±13.2, non-AKI: 17.9±13.8 | India | Group with and group without acute kidney injury (AKI), eGFR calculated using both serum creatinine and cystatin C based equations - latter one is more reliable |
| **Supplementary Table S6** (continued) | | | | | | | | | | |
| **PMID/**  **DOI** | **Number of children and sex** | **Age** | **Population(s)** | **Etiology liver disease** | **Value parameter (-P value – factor change)** | **Child-Pugh score** | **Distribution Child-Pugh** | **PELD/**  **MELD score** | **Country** | **Remarks** |
| 35696710 | Alagille: 156 (57.7% male), BA: 312 (39.4% male) | Median age (IQR); Alagille: 3.0 (2.0-8.5) years, BA: 3.0 (2.0-8.5) years | Alagille: children (21 or younger) undergoing first-time LT for Alagille syndrome, BA: age matched LT recipients with BA. | Alagille: 100% Alagille syndrome, BA: 100% BA | Median eGFR (IQR); Alagille: 110.4 (80.3-148.3) ml/min/1.73m2, BA: 144.6 (113.9-183.6) | NR | NR | Median PELD/MELD (IQR range); Alagille: 14.0 (9.0-20.0), BA: 8.0 (1.0-16.0) | US | eGFR at time of LT, Revised Schwartz equation for eGFR |
| 22994862 | 8976 (48% male) | Median age (IQR): 2.3 (0.8, 10.3) | Children who received a first LT | 39% BA, 13% other cholestatic, 14% fulminant liver failure, 14% metabolic disease (α1-acid antitrypsin deficiency, CF, hemochromatosis, homozygous hypercholesterolemia, glycogen storage disease, maple syrup urine disease, primary hyperoxaluria, tyrosinemia, Wilson's disease, other metabolic), 9% cirrhosis, 5% tumor, 6% other | Median eGFR (IQR): 106.7 (79.8, 139.4) ml/min/1.73m2 | NR | NR | Median PELD/MELD (IQR range): 18 (11-27) | US | Pre-transplant eGFR, eGFR calculated using bedside CKiD formula (based on serum creatinine) |
| 31571364 | 10 (50% male) | Median age (range): 2.3 years (1-7) years | Children with Alagille syndrome who underwent LT | 100% Alagille syndrome, 30% cirrhosis | Median eGFR: (range): 142 (103-150) ml/min/1.73m2 | NR | NR | Median PELD(range): 11 (1-35) | India | eGFR calculated using creatinine-based Schwartz equation |

**Supplementary Table S6** (continued)

| **PMID/**  **DOI** | **Number of children and sex** | **Age** | **Population(s)** | **Etiology liver disease** | **Value parameter (-P value – factor change)** | **Child-Pugh score** | **Distribution Child-Pugh** | **PELD/**  **MELD score** | **Country** | **Remarks** |
| --- | --- | --- | --- | --- | --- | --- | --- | --- | --- | --- |
| 36624564 | 136 (37.6% male) | Mean age ± SD: 3.7±4.6 | Children who underwent LT | 76.9% BA, 10.3% acute liver failure, 5.1% Alagille syndrome, 7.7% other (idiopathic neonatal hepatitis/cirrhosis, Wilson's disease, primary sclerosing cholangitis, portal vein atresia, citrullinemia, congenital pyruvate carboxylase deficiency) | Mean eGFR ± SD: 180±62 ml/min/1.73 m2 | NR | NR | Mean PELD±SD: 15.8±9.4, mean MELD: 17.2±6.8 - mean PELD/MELD=16.5 | Japan | Creatinine based eGFR at transplant |
| 16939513 | 51 (both groups: 52% male) | Mean age (range): 4.3 (1.1-19.3) years | Children with BA undergoing LT | 100% BA | Mean cGFR(range): 118.7 (47.8-331) ml/min/1.73 m2 | Mean Child-Pugh: 8.4 | NR | Mean PELD (range): 8.1 (1-19) | Taiwan | Grouped into <10 kg and >10 kg, only used data from children >10 kg, preoperative cGFR (calculated with Counahan formula (creatinine based)) |

Abbreviations: *BA* biliary atresia, *CP* Child-Pugh, *GFR* glomerular filtration rate, *HI* hepatic impairment, *IQR* interquartile range, *LT* liver transplantation, *NR* not reported, *SD* standard deviation.

**Supplementary Table S7** Characteristics of studies included in the literature search for plotting and additional information on alterations in functional liver mass in children with liver cirrhosis.

| **PMID/**  **DOI** | **Number of children and sex** | **Age** | **Population(s)** | **Etiology liver disease** | **Value parameter (-P value – factor change)** | **Child-Pugh score** | **Distribution Child-Pugh** | **PELD/**  **MELD score** | **Country** | **Remarks** |
| --- | --- | --- | --- | --- | --- | --- | --- | --- | --- | --- |
| 28834223 | With cirrhosis without BA: 13, with cirrhosis with BA: 33, without cirrhosis: 6 | Median age (95%CI); with cirrhosis without BA: 5.6 (2.5-8.8) years, with cirrhosis with BA: 0.8 (0.7-1.0) years, without cirrhosis: 2.3 (1.4-3.5) years | Children who received a primary LT from a living related donor | All groups: 63.5% BA, 9.6% liver malignancy, 5.8% Alagille syndrome, 5.8% metabolic diseases, 3.8% progressive familial intrahepatic cholestasis, 3.8% cirrhosis of unknown etiology, 3.8% auto-immune hepatitis, 1.9% primary sclerosing cholangitis, 1.9% non-syndromic bile duct paucity | Median native liver weight/body weight (95% CI); with cirrhosis without BA: 3.9 (2.7-4.3) %, with cirrhosis with BA: 5.9 (5.4-6.1) %, without cirrhosis: 4.1 (3.2-4.9) % - with cirrhosis without BA: factor 95%, with cirrhosis with BA: factor 144% | NR | NR | Median PELD (95% CI); with cirrhosis without BA: 13.4 (3.8-22.6), with cirrhosis with BA: 21.7 (18.3-25.6), without cirrhosis: 0 (0.0-5.1) | Belgium | Groups: cirrhosis with or without BA, and without cirrhosis; all received LT. Native liver weighed immediately after hepatectomy |
| 28417522 | 104 (55% male) | Mean age (SD, range); 3.9 (4.6, 0.1-17.4) years | children who received a LT | 46% BA, 4% acute liver failure, 4% primary sclerosing cholangitis, 6% liver tumors, 3% Alagille syndrome, 5% progressive familial intrahepatic cholestasis, 15% re-transplantation, 3% congenital hepatic fibrosis, 2% intestinal failure-associated liver disease, 3% CF, 3% cryptogenic cirrhosis, 9% miscellaneous | Mean native organ-to-recipient weight ratio ± SD: 4.2±1.9 | NR | NR | Mean PELD±SD: 17.1±11.3 | Italy |  |

| **Supplementary Table S7** (continued) | | | | | | | | | | |
| --- | --- | --- | --- | --- | --- | --- | --- | --- | --- | --- |
| **PMID/**  **DOI** | **Number of children and sex** | **Age** | **Population(s)** | **Etiology liver disease** | **Value parameter (-P value – factor change)** | **Child-Pugh score** | **Distribution Child-Pugh** | **PELD/**  **MELD score** | **Country** | **Remarks** |
| 29705277 | 8 (12.5% male) | Age (years); patient 1: 1, patient 2: 13, patient 3: 0, patient4: 0, patient 5: 14, patient 6: 1, patient 7: 1, patient 8: 0 | children with BA who underwent LT | 100% BA | Endotoxin activity before LT; patient 1: 0.38, patient 2: 0.4, patient 3: 0.26, patient 4: 0.39, patient 5: 0.23, patient 6: 0.17, patient 7: 0.06, patient 8: 0.32 | NR | NR | PELD/MELD; patient 1: 19, patient 2: 23, patient 3: 10, patient 4: 32, patient 5: 12, patient 6: 0, patient 7: 0, patient 8: 5 | Japan |  |
| 22677614 | 17 (47% male) | Age (months): patient 1: 10, patient 2: 12, patient 3: 27, patient 4: 14, patient 5: 12, patient 6: 9, patient 7: 124, patient 8: 64, patient 9:6, patient 10: 12, patient 11: 229, patient 12: 181, patient 13: 153, patient 14: 11, patient 15: 21, patient 16: 193, patient 17: 168 | children who underwent LDLT | 71% BA, 18% ornithine transcarbamylase deficiency, 6% graft failure, 6% primary sclerosing cholangitis | Endotoxin activity before LT; patient 1: 0.24, patient 2: 0.22, patient 3: 0.16, patient 4: 0.06, patient 5: 0.01, patient 6: 0.16, patient 7: 0.36, patient 8: 0.29, patient 9: 0.08, patient 10: 0.03, patient 11: 0.24, patient 12: 0.02, patient 13: 0.26, patient 14: 0.06, patient 15: 0.13, patient 16: 0.05, patient 17: 0.42 | NR | NR | PELD/MELD; patient 1: -9.3, patient 2: 26.9, patient 3: 5.7 patient 4: 11.3, patient 5: 10.7, patient 6: 19.5, patient 7: -4.0, patient 8: -15.8, patient 9: 31.3, patient 10: 11.8, patient 11: -2.0, patient 12: -2.3, patient 13: -6.2, patient 14: -2.2, patient 15: -6.4, patient 16: -7.9, patient 17:7.9 | Japan |  |

| **Supplementary Table S7** (continued) | | | | | | | | | | |
| --- | --- | --- | --- | --- | --- | --- | --- | --- | --- | --- |
| **PMID/**  **DOI** | **Number of children and sex** | **Age** | **Population(s)** | **Etiology liver disease** | **Value parameter (-P value – factor change)** | **Child-Pugh score** | **Distribution Child-Pugh** | **PELD/**  **MELD score** | **Country** | **Remarks** |
| 36978697 | Group 1: 11 (63.6% male), group 2: 19 (63.2 % male) | Mean age (range); group 1: 15 (10-17) years, group 2:14.6 (8-18 years) | Children with BA with a native liver after Kasai portoenterostomy | Both groups: 100% BA | Mean liver volume ± SD; group 1: 1140.94±134.62 cm3, group 2: 954.88±218.31 cm3 | NR | NR | Median MELD; group 1: 7, group 2:9. Mean PELD; group 1: -9.5, group 2: -8.5. | Italy | Group 1: patients with ideal medical outcomes (laboratory parameter values in normal range and no evidence of chronic liver disease complications, group 2: nonideal medical outcomes |
| 22464833 | Group A: 19 (26.3% male), group B: 12 (50% male) | Mean age ± SD at CT examination; group A: 5.9±5.1 years, group B: 1.2±2.2 years | Children with BA who underwent a Kasai operation | Both groups: 100% BA | Mean volume index; caudate segment: group A: 58±69 mm3, group B:105±273. left hepatic lobe: group A: 1860±5559, group B: 385±226 | NR | NR | Mean PELD/MELD±SD, group A: 12±9, group B: 13±12 | Taiwan | One group with esophageal variceal bleeding (EVB) (group A), and one group without EVB (group B). |
| 22903261 | 21 (38.1% male) | Median age (range): 1.4 (0.5-16.1) years | Children with BA who underwent LT | 100% BA | Hepatic clearance of beta-D glucan against the PELD score: see paragraph | NR | NR | Median PELD (range): 5.7 (13.4-31.3) | Japan |  |

| **Supplementary Table S7** (continued) | | | | | | | | | | |
| --- | --- | --- | --- | --- | --- | --- | --- | --- | --- | --- |
| **PMID/**  **DOI** | **Number of children and sex** | **Age** | **Population(s)** | **Etiology liver disease** | **Value parameter (-P value – factor change)** | **Child-Pugh score** | **Distribution Child-Pugh** | **PELD/**  **MELD score** | **Country** | **Remarks** |
| 22816448 | Group 1: 28, group 2: 64 | Median age (IQR) at measurement; group 1: 2.0 (0.8-13.0), group 2: 7.4 (3.0-13.1) | Children with chronic liver disease | Group 1: 42.9% BA, 14.3% metabolic liver disease, 17.9% autosomal recessive polycystic kidney disease, 3.57% intestinal failure associated liver disease, 7.14% Alagille syndrome, 3.57% Budd-Chiari syndrome, 3.57% primary sclerosing cholangitis, 7.14% miscellaneous. Group 2: 37.5% BA, 17.2% metabolic liver disease, 7.81 % autosomal recessive polycystic kidney disease, 9.38% autoimmune hepatitis, 7.81% intestinal failure associated liver disease, 1.56% Alagille syndrome, 1.56% Budd-Chiari syndrome, 3.13% Mulibrey nanism, 1.56% primary sclerosing cholangitis, 12.5% miscellaneous. | Median galactose half-life (Gal1/2) (IQR); group 1: 17.0 (12.5-28.5) min, group 2: 10.5 (9.5-12.5) min | NR | NR | Median PELD/MELD (IQR); group 1: 13 (-1 - 23), group 2: -1 (-8 - 8) | Finland | Group 1: children listed for LT or died within 1 year, group 2: children who survived without listing beyond 1 year. |

Abbreviations: *BA* biliary atresia, *CP* Child-Pugh, *HI* hepatic impairment, *IQR* interquartile range, *LT* liver transplantation, *NR* not reported, *SD* standard deviation.

**Supplementary Table S8** Characteristics of studies included in the literature search for plotting and additional information on alterations in portal blood flow in children with liver cirrhosis.

| **PMID/**  **DOI** | **Number of children and sex** | **Age** | **Population(s)** | **Etiology liver disease** | **Value parameter (-P value – factor change)** | **Child-Pugh score** | **Distribution Child-Pugh** | **PELD/**  **MELD score** | **Country** | **Remarks** |
| --- | --- | --- | --- | --- | --- | --- | --- | --- | --- | --- |
| 28834223 | With cirrhosis without BA: 13, with cirrhosis with BA: 33, without cirrhosis: 6 | Median age (95%CI); with cirrhosis without BA: 5.6 (2.5-8.8) years, with cirrhosis with BA: 0.8 (0.7-1.0) years, without cirrhosis: 2.3 (1.4-3.5) years | Children who received a primary LT from a living related donor | All groups: 63.5% BA, 9.6% liver malignancy, 5.8% Alagille syndrome, 5.8% metabolic diseases, 3.8% progressive familial intrahepatic cholestasis, 3.8% cirrhosis of unknown etiology, 3.8% auto-immune hepatitis, 1.9% primary sclerosing cholangitis, 1.9% non-syndromic bile duct paucity | Median PV flow (95% CI); with cirrhosis without BA: 261 (122-382) ml/min, with cirrhosis with BA: 78 (51-105) ml/min, without cirrhosis: 230 (91-321) ml/min. Median PV flow/100 g of liver; with cirrhosis without BA: 34 (16-54) ml/min/100 g, with cirrhosis with BA: 7 (4-11) ml/min/100 g, without cirrhosis: 64 (56-71) ml/min/100 g - significantly lower in children without cirrhosis compared to children with cirrhosis- factor 18.75%. | NR | NR | Median PELD (95% CI); with cirrhosis without BA: 13.4 (3.8-22.6), with cirrhosis with BA: 21.7 (18.3-25.6), without cirrhosis: 0 (0.0-5.1) | Belgium | Doppler US of native liver. PV flow calculated with portal vein velocity and internal diameter. Pretransplant PV flow was negatively correlated with PELD score. |
| 8792268 | Liver disease: 18 (38.9 % male), ETC: 9, EC: 5, NC: 4, controls: 20 (45% male) | Mean age (range); liver disease: 4.8 (0.1-11) years, controls: 6 (0.1-12) years | Liver disease: Children admitted to the hospital for evaluation of unknown liver disease, without decompensated liver disease. Controls: without evidence of liver disease. | Liver disease: 33.3% progressive familial intrahepatic cholestasis, 16,7% neonatal hepatitis, 5.56% glycogen storage disease, 16.7% primary sclerosing cholangitis, 5.56% extrahepatic BA, 5.56% cryptogenic cirrhosis, 5.56% Wilson's disease, 5.56% metabolic disease with severe steatosis, 5.56% unknown | Mean portal vein velocity ± SD; controls: 31.0±SD1.4 cm/s, EC: 20.8±SD4.0 cm/s, ETC: 15.1±SD4.2 cm/s, NC: 30.8±SD3.5 - EC vs NC factor 68% | NR | NR | NR | Saudi Arabia | Liver disease; 3 groups: established cirrhosis (ETC), early cirrhosis (EC), no cirrhosis (NC), according to histologic evaluation. Pulsed Doppler to measure PVV. |

**Supplementary Table S8** (continued)

| **PMID/**  **DOI** | **Number of children and sex** | **Age** | **Population(s)** | **Etiology liver disease** | **Value parameter (-P value – factor change)** | **Child-Pugh score** | **Distribution Child-Pugh** | **PELD/**  **MELD score** | **Country** | **Remarks** |
| --- | --- | --- | --- | --- | --- | --- | --- | --- | --- | --- |
| 9799319 | Liver disease: 22 (54.5% male), cirrhosis: 12 cirrhosis (41,7% male), chronic active hepatitis: 10 (70% male), controls: 8 | Mean age (range); liver disease: 7.5 years (0.5-16), controls: 8.9 years | Liver disease: children with cirrhosis or chronic active hepatitis under investigation for liver disease, without decompensated liver disease. Controls: without liver disease. | Cirrhosis: 25% chronic hepatitis B, 8.3% galactosaemia, 25% Wilson's disease, 8.3% progressive familial intrahepatic cholestasis, 8.3% unknown, 8.3% glycogen storage disease, 8.3% congenital hepatic fibrosis, 8.3% selective IgA deficiency with autoimmune hepatitis | Mean portal vein velocity; cirrhosis: 18 cm/sec, controls: 33 cm/sec - P<0.05 - factor 54.5% | NR | NR | NR | Turkey | Cirrhosis identified with histopathology of liver biopsies. Doppler US to measure PVV. |
| 7472909 | Liver disease: 45 (71% male), cirrhosis: 7, controls: 11 (64% male) | Mean age (range); liver disease: 11.4 (2.6-20.8) years, controls: 11.4 (7.3-14.4) years | Children with liver disease | Liver disease: 15.6 % HBsAg-positive asymptomatic carrier, 22.2% chronic persistent hepatitis, 15.6% liver cirrhosis, 2.22% Alagille's syndrome, 2.22% chronic active Epstein-Barr virus infection, 2.22% chronic myelomonocytic leukemia, 2.22% congenital hepatic fibrosis, 2.22% primary sclerosing cholangitis, 2.22% glycogen storage disease, 2.22% graft versus host disease after bone marrow transplantation | Mean maximal velocity in main portal trunkhepatopetal flow in portal trunk ± SD; controls: 69.8±16.3 cm/s, liver cirrhosis: 30.7±10.5 cm/s - P<0.01 - factor 44% | NR | NR | NR | Japan | Cirrhosis diagnosed histologically by biopsy, except one: nodular finding on MRI. Doppler to measure PVV. |
| 26256082 | PVT: 9 (44% male), non-PVT= 119 (51.56% male) | Mean age ± SD; PVT: 1±0.273 years, non-PVT: 0.976±0.536 | Children with BA who underwent LT | 100% BA | Mean maximum portal vein velocity ± SD; PVT: 19.82±3.21 cm/s, non-PVT: 23.17±8.38 cm/s | NR | NR | Mean PELD±SD: PVT: 16.47±4.80, non-PVT: 17.28±6.61 | China | Measurement before LT. Preoperative values compared between patients with early portal vein thrombosis (PVT) and those without PVT after LT. Doppler us within seven days before LT. |

| **Supplementary Table S8** (continued) | | | | | | | | | | |
| --- | --- | --- | --- | --- | --- | --- | --- | --- | --- | --- |
| **PMID/**  **DOI** | **Number of children and sex** | **Age** | **Population(s)** | **Etiology liver disease** | **Value parameter (-P value – factor change)** | **Child-Pugh score** | **Distribution Child-Pugh** | **PELD/**  **MELD score** | **Country** | **Remarks** |
| 35460136 | PVT: 10 (50% male), non-PVT: 105 (52% male) | Median age (IQR); PVT: 0.58 years (4-30 months), non-PVT: 1.08 years (5-79 months) | Children who underwent LT | Both groups together: 56% BA, 7% fulminant hepatic failure, 6% progressive familial intrahepatic cholestasis, 5% CF, 3% hepatoblastoma, and others. | Median portal vein flow velocity (IQR); PVT: 16.5 (12–19.5) cm/s, non-PVT: 20 (15–24) cm/s | NR | NR | Median PELD (IQR); PVT: 21 (5.6-24.1), non-PVT: 17.9 (3.9-27) | Germany | Groups: PVT and non-PVT after LT. Doppler US to measure PV flow velocity preoperatively. |
| 20223318 | 11 (36% male) | Mean age (range): 11 (7-16) months | Children who underwent LT | 90.9% BA, 9.09% Alagille syndrome | Mean portal vein flow ± SD; donor: 124±60 ml/min/100 g liver weight, pretransplant in recipient: 20±17 ml/min/100 g liver weight - P0.0003 - factor 16% | NR | 27.3% B, 72.7% C | NR | Japan | Measurements done in only 7 cases, demographics from all 11 children. Intraoperative blood flow measurements done with ultrasonic transit time flow meter. Portal vein flow measured before removal native liver. Donor values used as control. |
| 17889162 | 53 (58.5% male) | Mean age (range): 3.67 years (1-164 months) | Children who underwent LT | 50% BA | Mean portal flow ± SD: 13±12.3 mL/min/kg | NR | NR | NR | Spain | Portal flow measured in native liver with ultrasonic transit-time probes. |

Abbreviations: *BA* biliary atresia, *CP* Child-Pugh, *HI* hepatic impairment, *IQR* interquartile range, *LT* liver transplantation, *NR* not reported, *SD* standard deviation.

**Supplementary Table S9** Characteristics of studies included in the literature search for plotting and additional information on alterations in hepatic arterial blood flow in children with liver cirrhosis.

| **PMID/**  **DOI** | **Number of children and sex** | **Age** | **Population(s)** | **Etiology liver disease** | **Value parameter (-P value – factor change)** | **Child-Pugh score** | **Distribution Child-Pugh** | **PELD/**  **MELD score** | **Country** | **Remarks** |
| --- | --- | --- | --- | --- | --- | --- | --- | --- | --- | --- |
| 20223318 | 11 (36% male) | Mean age (range): 11 (7-16) months | Children who underwent LT | 90.9% BA, 9.09 % Alagille syndrome | Mean hepatic artery flow ± SD; donor: 55±34 ml/min/100 g liver weight, pretransplant in recipient: 36±11 ml/min/100 g liver weight | NR | 27.3% B, 72.7% C | NR | Japan | Measurements done in only 7 cases, demographics from all 11 children. Intraoperative blood flow measurements done with ultrasonic transit time flow meter. Hepatic artery flow measured before removal native liver. Calculated as sum of right HAF, middle HAF, and left HAF. Donor values used as control. |
| 28834223 | With cirrhosis without BA: 13, with cirrhosis with BA: 33, without cirrhosis: 6 | Median age (95%CI); with cirrhosis without BA: 5.6 (2.5-8.8) years, with cirrhosis with BA: 0.8 (0.7-1.0) years, without cirrhosis: 2.3 (1.4-3.5) years | Children who received a primary LT from a living related donor | All groups: 63.5% BA, 9.6% liver malignancy, 5.8% Alagille syndrome, 5.8% metabolic diseases, 3.8% progressive familial intrahepatic cholestasis, 3.8% cirrhosis of unknown etiology, 3.8% auto-immune hepatitis, 1.9% primary sclerosing cholangitis, 1.9% non-syndromic bile duct paucity | Median hepatic artery flow (95% CI); with cirrhosis without BA: 31 (18-47) ml/min/100 g of liver, with cirrhosis with BA: 17 (13-22) ml/min/100 g of liver, without cirrhosis: 19 (5-29) ml/min/100 g of liver - HA flow similar in children with cirrhosis and children without cirrhosis - factor 105% | NR | NR | Median PELD (95% CI); with cirrhosis without BA: 13.4 (3.8-22.6), with cirrhosis with BA: 21.7 (18.3-25.6), without cirrhosis: 0 (0.0-5.1) | Belgium | Doppler US of native liver. |
| 17889162 | 53 (58.5% male) | Mean age (range): 3.67 years (1-164 months) | Children who underwent LT | 50% BA | Mean arterial flow ± SD: 10.4±8 mL/min/kg | NR | NR | NR | Spain | Portal flow measured in native liver with ultrasonic transit-time probes. |

Abbreviations: *BA* biliary atresia, *CP* Child-Pugh, *HI* hepatic impairment, *LT* liver transplantation, *NR* not reported, *SD* standard deviation.

**Supplementary Table S10** Characteristics of studies included in the literature search for plotting and additional information on alterations in renal blood flow in children with liver cirrhosis.

| **PMID/**  **DOI** | **Number of children and sex** | **Age** | **Population(s)** | **Etiology liver disease** | **Value parameter (-P value – factor change)** | **Child-Pugh score** | **Distribution Child-Pugh** | **PELD/**  **MELD score** | **Country** | **Remarks** |
| --- | --- | --- | --- | --- | --- | --- | --- | --- | --- | --- |
| 10603136 | Cirrhosis: 21 (76% male), ascitic: 10, non-ascitic: 11, controls: 10 (70% male) | Mean age ± SD; cirrhosis: 9.6±3.9 years, ascitic: 9.7±4.2 years, non-ascitic: 9.5±3.8 years | Cirrhosis: children with pathology proven liver cirrhosis and normal kidney function. Controls: matched children without renal or hepatic diseases. | NR | Mean RRI ±SD; ascitic: 0.76±0.07, non-ascitic 0.69±0.07, controls: 0.46±0.02. non-ascitic vs controls P0<0.001. CPA: 0.69±0.06, CPB: 0.68±0.07, CPC: 0.78±0.05 | NR | 23.8% A, 38.1% B, 38.1% C | NR | Egypt | Cirrhosis divided in two groups: ascitic and non-ascitic. Positive relationship between CP score and RRI. RRI measured with duplex Doppler. |
| DOI: 10.1177/8756479310374363 | Cirrhosis: 30 (40% male), controls: 10 | Mean age ± SD; cirrhosis: 9.7±3.1 years | Cirrhosis: children with pathology-proven liver cirrhosis and normal kidney function. Controls: healthy matched children. | NR | Mean RRI±SD; CPA: 0.56±0.1, CPB: 0.68±0.07, CPC:0.78±0.05, controls: 0.36±0.04 - cirrhosis: each CP class significantly different from control group | NR | 33.3%A, 33.3% B, 33.3% C | NR | Egypt | RRI measured with Doppler spectral wave analysis. RRI correlated positively with CP score. |
| 35067841 | Cases: 51, controls: 48, both groups together: 58.6% male | Both groups together; median age(range): 11 (6-18) years | Cases: non-azotemic cirrhotic children with tense ascites. Controls: children with chronic liver disease without ascites. | Cases: 43.1% Wilson's disease, 41.2% autoimmune liver disease, 1.96% chronic hepatitis B, 13.7% cryptogenic. Controls: 25.5% Wilson's disease, 58.3% autoimmune liver disease, 6.25% chronic hepatitis B, 8.33% cryptogenic. | Median baseline RRI (range); cases: 0.71 ( 0.49-0.82), controls: 0.54 (0.42-0.72) | NR | NR | Median PELD (range); cases: 23 (11 to 39), controls: 4(-8 to 15) | India | PELD score had a strong positive correlation with baseline RRI. RRI measured with doppler ultrasonography. |

| **Supplementary Table S10** (continued) | | | | | | | | | | |
| --- | --- | --- | --- | --- | --- | --- | --- | --- | --- | --- |
| **PMID/**  **DOI** | **Number of children and sex** | **Age** | **Population(s)** | **Etiology liver disease** | **Value parameter (-P value – factor change)** | **Child-Pugh score** | **Distribution Child-Pugh** | **PELD/**  **MELD score** | **Country** | **Remarks** |
| 33978827 | AKI: 41 (68% male), non-AKI: 59 (50.8% male) | Mean age ± SD; AKI: 9.2±5.3 years, non-AKI: 7.7±5.4 years | children with diagnosis of chronic liver disease with portal hypertension | AKI: 27% autoimmune hepatitis, 24% Wilson's disease, 22% BA, 10% progressive familial intrahepatic cholestasis, 5% hepatitis B, 5% primary sclerosing cholangitis, 5% Alagille syndrome | Mean RRI±SD; AKI: 0.88±0.09, non-AKI: 0.76±0.08 | NR | NR | Mean PELD±SD; AKI: 25.4±13.2, non-AKI: 17.9±13.8 | India | Group with and group without acute kidney injury (AKI). RRI measured with renal color Doppler ultrasonography. |
| 15043668 | Cirrhosis: 29 (51.7% male), controls: 20 (50% male) | Mean age ± SD; cirrhosis: 11.6±3.5 years, controls: 10.3±3.4 years | Cirrhosis: children with pathology-proven cirrhosis and normal kidney function, controls: healthy children | CP≤ 6: 27.8% chronic viral hepatitis, 44.4% metabolic liver diseases (75% Wilson's disease, 12.5 % glycogen storage type Ib, 12.5% gaucher disease), 5.56% autoimmune, 22.2% cryptogenic. CP>6: 36.4% metabolic liver diseases (75% Wilson's disease, 25 % glycogen storage type Ib), 18.2% autoimmune, 36.4% cholestatic liver diseases (25% BA, 75% intrahepatic cholestasis), 9.09% cryptogenic. | Mean RRI±SD; cirrhosis: 0.69±0.07, controls: 0.62±0.02. CP≤ 6: 0.67±0.08, CP>6: 0.73±0.05. | NR | 62% CP≤6, 38% CP>6 | NR | Turkey | RRI measured with Doppler ultrasonography. RRI increased with decompensated cirrhosis compared to compensated cirrhosis. |

Abbreviations: *BA* biliary atresia, *CP* Child-Pugh, *HI* hepatic impairment, *LT* liver transplantation, *NR* not reported, *RRI* renal resistive index, *SD* standard deviation.

**Supplementary Table S11** Characteristics of studies included in the literature search for plotting and additional information on cardiac index alterations in renal blood flow in children with liver cirrhosis.

| **PMID/**  **DOI** | **Number of children and sex** | **Age** | **Population(s)** | **Etiology liver disease** | **Value parameter (-P value – factor change)** | **Child-Pugh score** | **Distribution Child-Pugh** | **PELD/**  **MELD score** | **Country** | **Remarks** |
| --- | --- | --- | --- | --- | --- | --- | --- | --- | --- | --- |
| 31900969 | 27 (40.7% male) | Mean age (range): 3.2 (8 months-13 years) years | Children with cirrhosis undergoing LT | 77.8% BA, 11.1% progressive familial intrahepatic cholestasis, 3.7% primary sclerosing cholangitis, 3.7% inborn error of bile acid metabolism, 3.7% methylmalonic acidemia | Mean baseline cardiac index ± SD: 4.5 ± 1.3 ml/min m2 | NR | NR | Mean PELD±SD: 12.4 ± 12.5 | Taiwan | Assumed unit L/min/m2 |
| 7699535 | 26 (53.8% male) | Age at investigations (range): 7 months- 16 years and 6 months | Children with cirrhosis and one child with portal vein obstruction, with pulmonary arteriovenous shunting | BA, polysplenia syndrome, Budd-Chiary syndrome, tyrosinemia, Hepatitis C virus, CF, portal vein obstruction, α1-acid antitrypsin deficiency, autoimmune hepatitis, unknown | Mean cardiac index (range): 7 (3.6-12.5) L/min/m2. Median cardiac index (range); children with BA and polysplenia syndrome: 9.7 (7.1-12.6) L/min/m2, remaining children: 6.1 (3.6-7.9) L/min/m2 -P<0.01 , other children (n=93) with cirrhosis without pulmonary arteriovenous shunt: 5.6 (3-9.2) L/min/m2 - not significantly different from children with pulmonary shunting without polysplenia syndrome | NR | NR | NR | France |  |
| 26462482 | 20 (45% male) | Median age (range): 1.8 years (8-108 months) | Cirrhotic children under the age of 15 on a waiting list for LT | 75% BA, 10% Alagille syndrome, 5% progressive familial intrahepatic cholestasis, 10% idiopathic | Median cardiac index (range): 5.4 (3.6-7.2) L/min/m2. Median LVEF (range): 74 (64-89) % | NR | NR | Median PELD (range): 19.5 (14-28) | Thailand | Pre-LT echocardiography |

**Supplementary Table S11** (continued)

| **PMID/**  **DOI** | **Number of children and sex** | **Age** | **Population(s)** | **Etiology liver disease** | **Value parameter (-P value – factor change)** | **Child-Pugh score** | **Distribution Child-Pugh** | **PELD/**  **MELD score** | **Country** | **Remarks** |
| --- | --- | --- | --- | --- | --- | --- | --- | --- | --- | --- |
| 28834223 | With cirrhosis without BA: 13, with cirrhosis with BA: 33, without cirrhosis: 6 | Median age (95%CI); with cirrhosis without BA: 5.6 (2.5-8.8) years, with cirrhosis with BA: 0.8 (0.7-1.0) years, without cirrhosis: 2.3 (1.4-3.5) years | Children who received a primary LT from a living related donor | All groups: 63.5% BA, 9.6% liver malignancy, 5.8% Alagille syndrome, 5.8% metabolic diseases, 3.8% progressive familial intrahepatic cholestasis, 3.8% cirrhosis of unknown etiology, 3.8% auto-immune hepatitis, 1.9% primary sclerosing cholangitis, 1.9% non-syndromic bile duct paucity | Pretransplant mean systemic arterial pressure, heartrate, respiratory rate, cardiac index, and left ventricular ejection fraction similar in children without cirrhosis and children with cirrhosis, regardless of presence of BA (data not shown) | NR | NR | Median PELD (95% CI); with cirrhosis without BA: 13.4 (3.8-22.6), with cirrhosis with BA: 21.7 (18.3-25.6), without cirrhosis: 0 (0.0-5.1) | Belgium | Cardiac output measured with transpulmonary thermodilution, cardiac index calculated with cold saline dissipation curve measured at site of arterial catheter |
| [10.23958/ijirms/vol07-i11/1553](https://doi.org/10.23958/ijirms/vol07-i11/1553) | 41 (53.7%male) | Mean age ± SD: 5.07±5.83 years | Children who underwent LT | 34.1% primary biliary causes. 17.1% fulminant, 9.8% progressive familial intrahepatic cholestasis, 7.3% urea cycle defect, 4.9% Wilson's disease, 4.9% Crigler Najjar, 4.9% hepatoblastoma, 2.4% hyperoxalocis, 2.4% cryptogenic, 2.4% viral, 2.4% hypercholesterolemia, 2.4% autoimmune, 2.4% Budd-Chiari syndrome, 2.4% Caroli congenital hepatic fibrosis | Mean baseline cardiac index ± SD: 5.3±2.27 L/min/m2 | NR | 22.1% A, 57.1% B, 20.7% C | Mean PELD±SD: 12.12±6.82 | Turkey | Cardiac index measured with PiCCO. Value at T_baseline_ was used. |

| **Supplementary Table S11** (continued) | | | | | | | | | | |
| --- | --- | --- | --- | --- | --- | --- | --- | --- | --- | --- |
| **PMID/**  **DOI** | **Number of children and sex** | **Age** | **Population(s)** | **Etiology liver disease** | **Value parameter (-P value – factor change)** | **Child-Pugh score** | **Distribution Child-Pugh** | **PELD/**  **MELD score** | **Country** | **Remarks** |
| 8201225 | 16 (56.3% male) | Mean age at time of LT±SD: 8.6±4.7 years | Children referred for LT due to α1-acid antitrypsin deficiency related liver disease | 100% α1-acid antitrypsin deficiency | Mean cardiac index ± SD: 5.6±1.1 L/min/m2 | NR | 18.75% A6, 18.75% B7, 37.5% B8, 6.25% B9, 6.25% C10, 6.25% C11, 6.25% C12 | NR | France | Pretransplant cardiac index. Hemodynamics with artery catheter in right intrarenal jugular vein and cardiac output measured with thermodilution method. |
| 36754885 | 12 (8.33% male) | Median age at time of LT (range): 10 years and 2 months (7 months to 22 years) | Children to young adults with portopulmonary hypertension who underwent LT | 75% BA, 25% portosystemic shunt | Mean cardiac index ± SD; before LT: 7.6±3.1 L/min/m2 before anti-PH treatment, 6.6±3.1 L/min/m2 after anti-PH treatment, after LT: 4.7±1.5 L/min/m2 - significantly reduced after LT compared to before LT | NR | NR | NR | Japan | Children with pre- and post-operative cardiac catheterization and echocardiogram data. |
| 3887884 | 73 (43% male) | Mean age ± SD; before LT:6.8±6.1 years, after LT:7.8±6.1 years | Children with chronic liver disease referred for LT | 56.2% BA, 17.8% intrahepatic cholestasis, 9.59% α1-acid antitrypsin deficiency, 2.74% glycogen storage disease type I, 1.37% CF, 6.85% chronic aggressive hepatitis, 1.37% neonatal hepatitis, 1.37% malignant tumor, 1.37% trauma | Mean cardiac index ± SD; before LT: 5.8±1.5 L/min/m2, after LT: 3.8±1.2 L/min/m2 - cardiac index reduced with a mean of 35% after LT - P<0.001, 82% of children cardiac index > 4 L/min/m2 | NR | NR | NR | USA | Evaluation with echocardiography. |

| **Supplementary Table S11** (continued) | | | | | | | | | | |
| --- | --- | --- | --- | --- | --- | --- | --- | --- | --- | --- |
| **PMID/**  **DOI** | **Number of children and sex** | **Age** | **Population(s)** | **Etiology liver disease** | **Value parameter (-P value – factor change)** | **Child-Pugh score** | **Distribution Child-Pugh** | **PELD/**  **MELD score** | **Country** | **Remarks** |
| 18537904 | 88 (48.9% male) | Median age (range): 7.35 years (6-194 months) | Children who underwent an evaluation for LT including an electrocardiogram and an echocardiogram, controls: age-and sex-matched controls | 35.2% metabolic liver disease, 19.3% BA, 14.8% autoimmune hepatitis, 11.4% cryptogenic cirrhosis, 5.68% chronic viral hepatitis caused by Hepatitis B virus infection, 5.68% congenital hepatic fibrosis, 4.55% progressive familial intrahepatic cholestasis type 1, 3.41% fulminant hepatic failure | Mean LVEF±SD: 76±14.4 % | NR | Study group excluding patients with acute liver failure (n=3): 15.3% A, 30.6% B, 54.1% C | Median PELD (range); study group excluding patients with acute live failure(n=3): 14 (-10-51) | Turkey | LVEF pre-LT. |
| 34497314 | 30 (53.3% male) | Mean age ± SD: 1.2±1.3 | Children with BA who underwent LT | 100% BA | Mean LVEF±SD: 60.50±4.49 % | NR | NR | Mean PELD±SD: 14.93±9.34 | Brazil | LVEF measured with echocardiography and calculated by Simpson's method, pre-LT. |
| 37901739 | Non-myocardial injury: 160 (48.8% male), myocardial injury: 142 (50.7% male) | Median age (IQR); non-myocardial injury: 0.8 years (6.0-16.8 months), myocardial injury: 0.6 years (6.0-11.0 months) | Children with BA who underwent LT | 100% BA | Median LVEF (IQR); non-myocardial injury: 64 (62-67) %, myocardial injury: 65 (62-67) % | NR | NR | Median PELD (IQR); non-myocardial injury: 14 (4-21), myocardial injury: 22 (12-28) | China | Two groups: myocardial injury and non-myocardial injury during LT. Pre-LT value. |
| 37407250 | Non-PRS: 172 (49.4% male), PRS: 132 (50.0% male) | Median age (IQR); non-PRS: 0.7 years (6-12 months), PRS: 0.7 years (6-12 months) | Children with BA who underwent LT | 100% BA | Mean LVEF±SD; non-PRS: 64.7±3.8 %, PRS: 64.0±3.3 % | NR | NR | Mean PELD±SD; non-PRS: 16.7±11.5, PRS: 17.1±10.7 | China | Two groups: postreperfusion syndrome and non-postreperfusion syndrome. |

| **Supplementary Table S11** (continued) | | | | | | | | | | |
| --- | --- | --- | --- | --- | --- | --- | --- | --- | --- | --- |
| **PMID/**  **DOI** | **Number of children and sex** | **Age** | **Population(s)** | **Etiology liver disease** | **Value parameter (-P value – factor change)** | **Child-Pugh score** | **Distribution Child-Pugh** | **PELD/**  **MELD score** | **Country** | **Remarks** |
| 29908011 | 39 (46.2% male) | Median age (IQR): 0.75 years (6-14 months) | Children with end-stage liver disease who underwent LT | 76.9% BA, 10.3% hepatoblastoma, 2.56% hepatocellular carcinoma, 2.56% neonatal hepatitis, 2.56% CMV hepatitis, 2.56% Wilson's disease, 2.56% familial intrahepatic cholestasis | Mean LVEF±SD: 74.3±6.7 % | NR | NR | Median PELD(IQR): 15 (10-20) | Korea |  |
| 12270348 | Patients: 22 (50% male), controls: 22 (50% male) | Mean age ± SD; patients: 5.0±4.6 years, controls: 4.1±3.5 years | Patients: children with biopsy-proven cirrhosis, controls: healthy age- and sex-matched children | Patients: 27.3% BA, 18.2% Wilson's disease, 9.09% Alagille syndrome, 9.09% idiopathic neonatal hepatitis leading to cirrhosis, 9.09% progressive familial intrahepatic cholestasis, 4.55% non-syndromic paucity of interlobular bile ducts, 4.55% tyrosinemia, 4.55% Gaucher's disease, 4.55% type IV glycogenosis, 4.55% familial cryptogenic cirrhosis, 4.55% erythropoietic protoporphyria | Mean LVEF±SD; patients: 68±10 %, controls: 69±10 - no significant difference - factor 98.6% | NR | 36.4% A, 13.6% B, 50.0% C | NR | Turkey |  |

Abbreviations: *BA* biliary atresia, *CP* Child-Pugh, *HI* hepatic impairment, *IQR* interquartile range, *LT* liver transplantation, *LVEF* left ventricular ejection fraction, *NR* not reported, *SD* standard deviation.

**Supplementary Figure S1** Interaction analysis of the relationship between MELD/PELD score and serum albumin concentrations (g/dL) in children and adults.


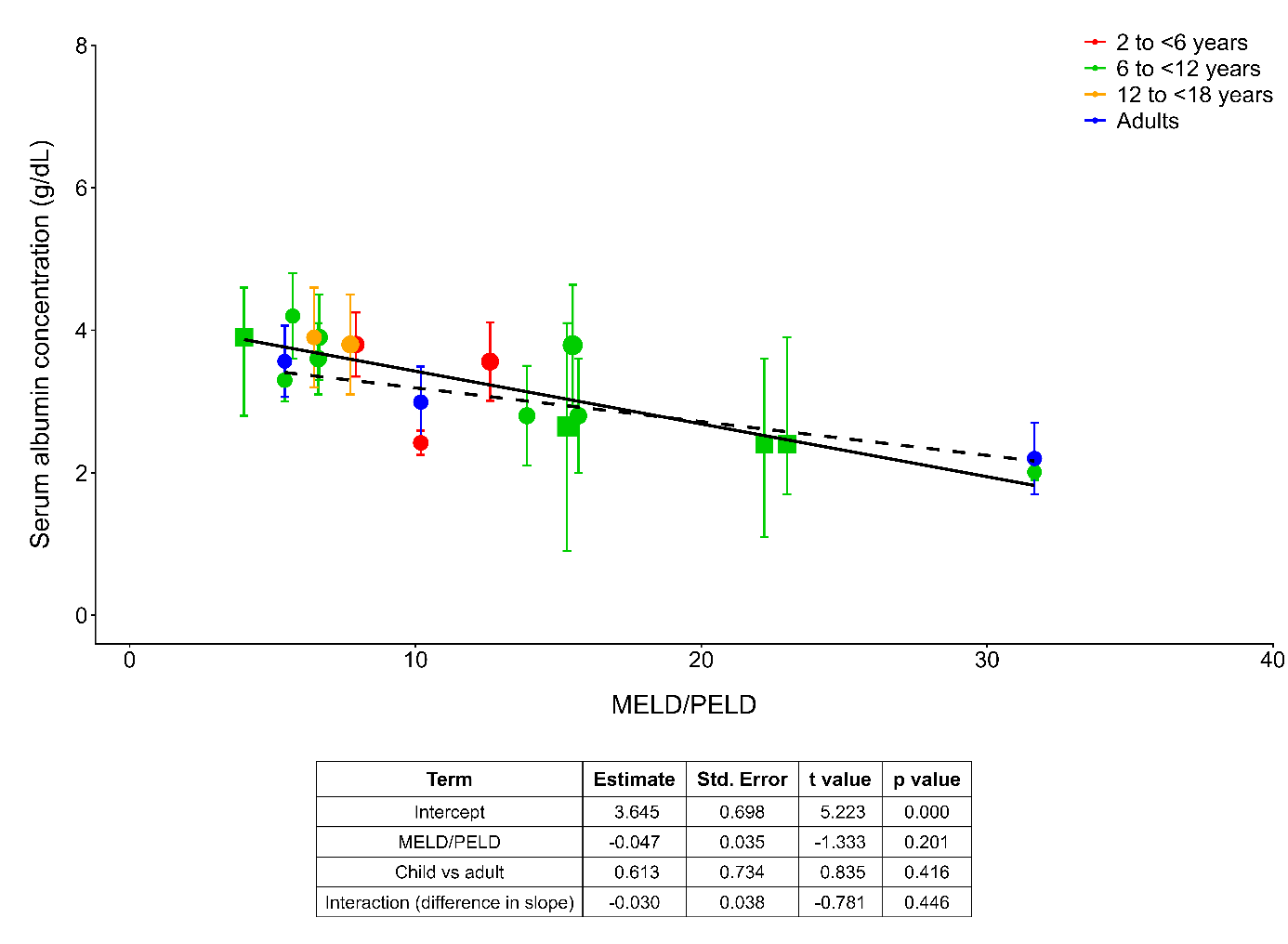


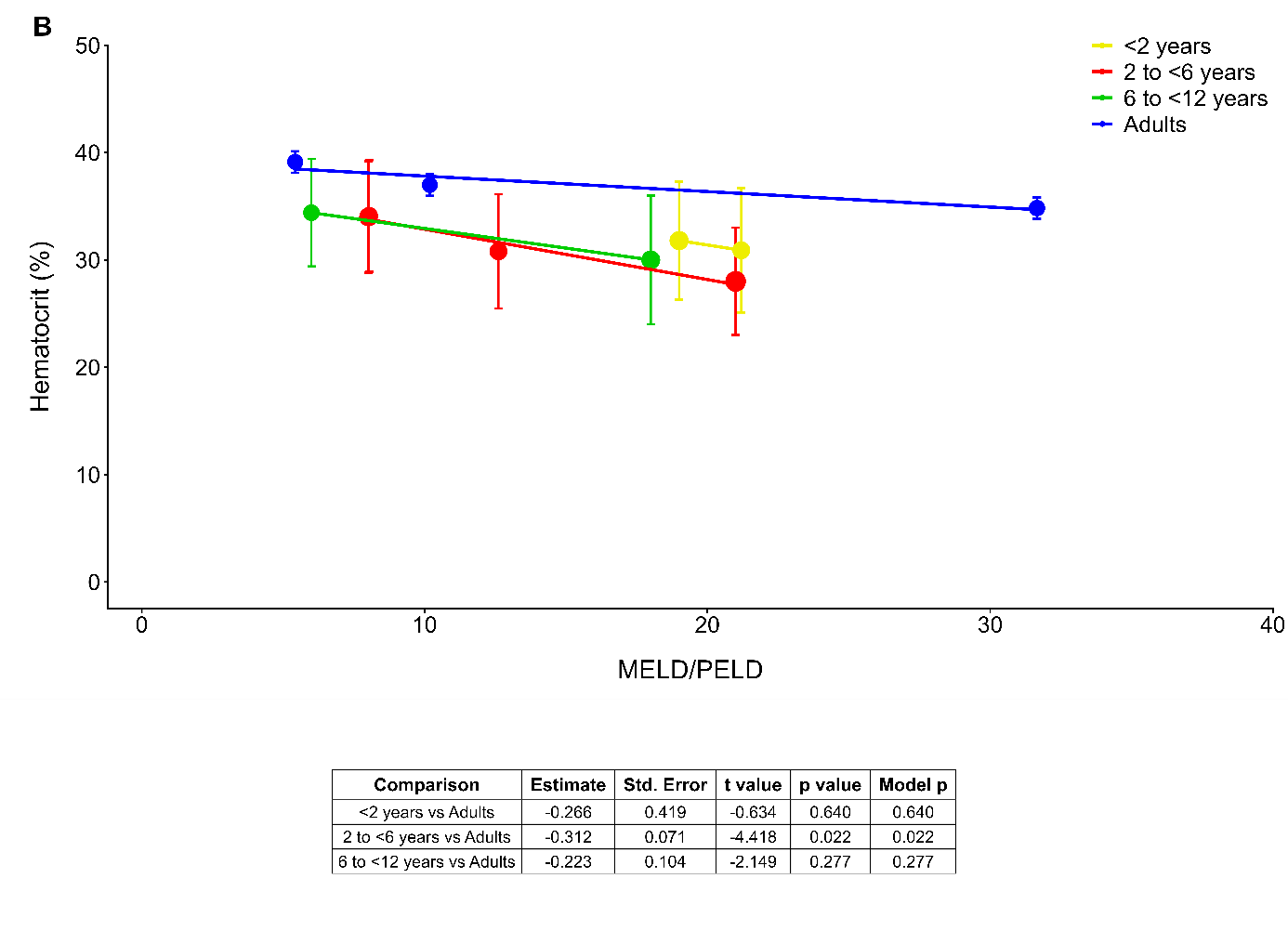

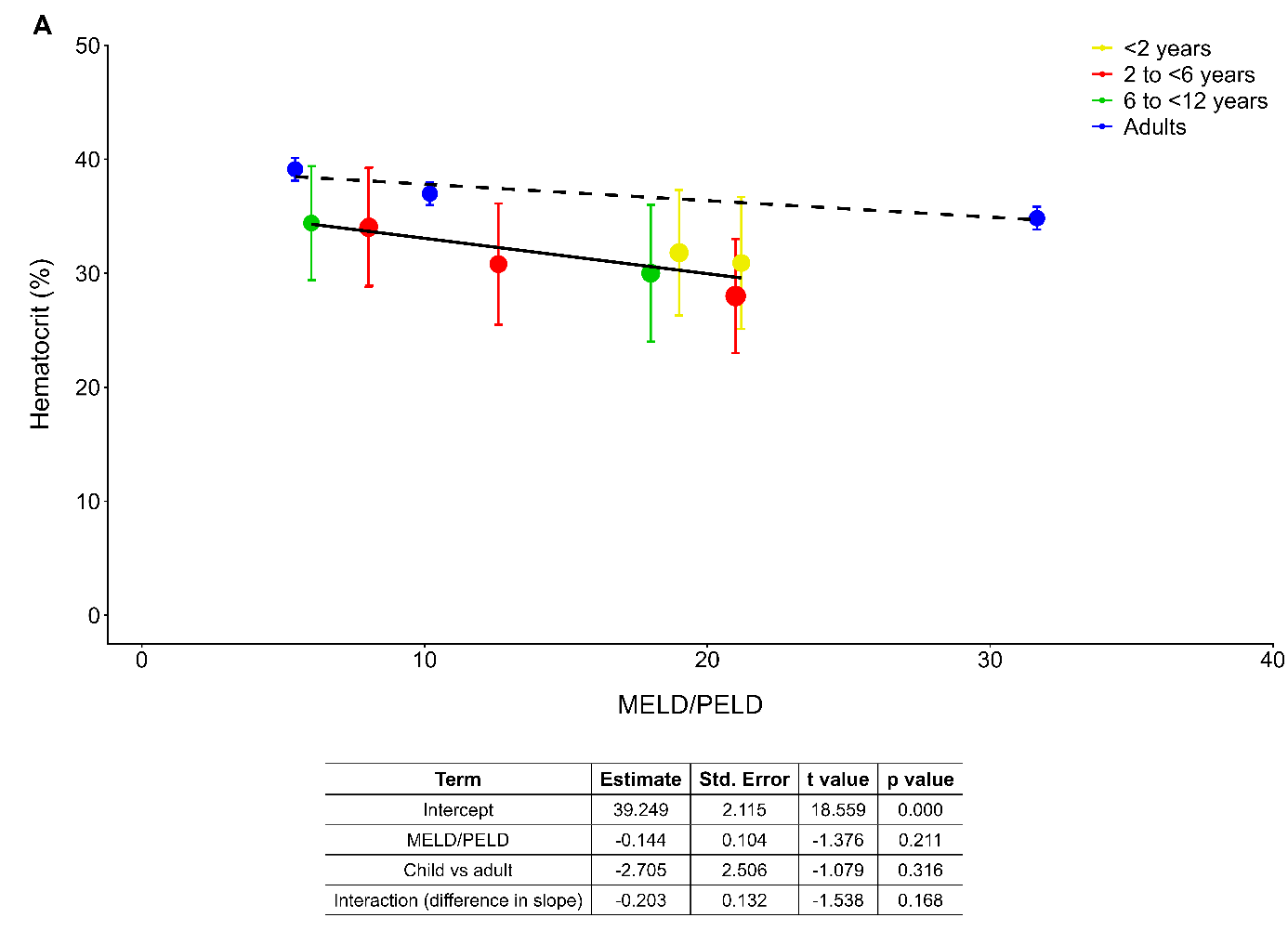
**Supplementary Figure S2** Interaction analysis of the relationship between MELD/PELD score and hematocrit (%) in children versus adults (**A**) and across pediatric age subgroups (**B**).

**Supplementary Figure S3** Interaction analysis of the relationship between MELD/PELD score and glomerular filtration rate (mL/min/1.73 m^2^) in children and adults.


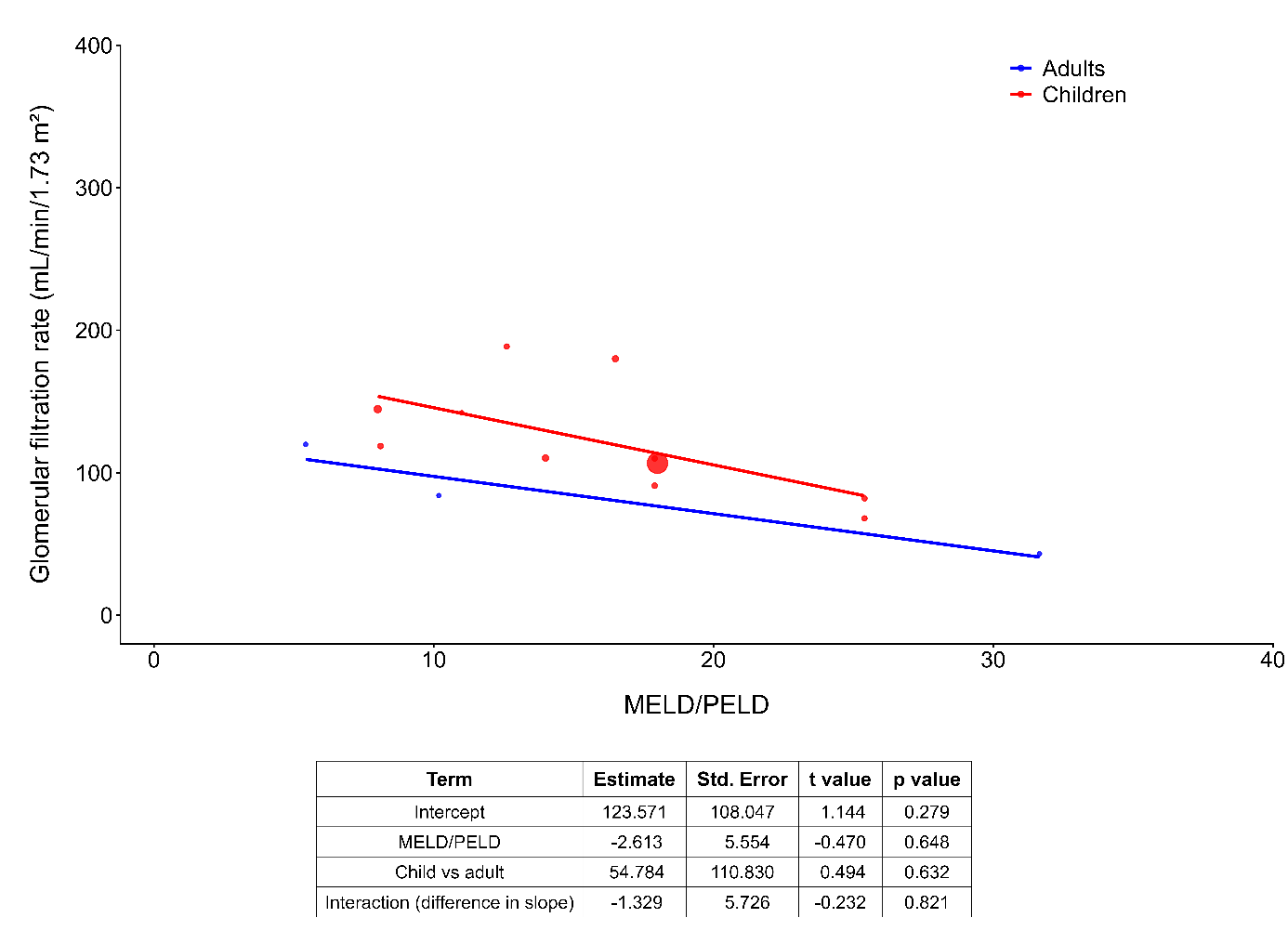


**Supplementary Figure S4** Interaction analysis of the relationship between PELD score and fractional portal blood flow (mL/min/100 g liver) in children and adults.


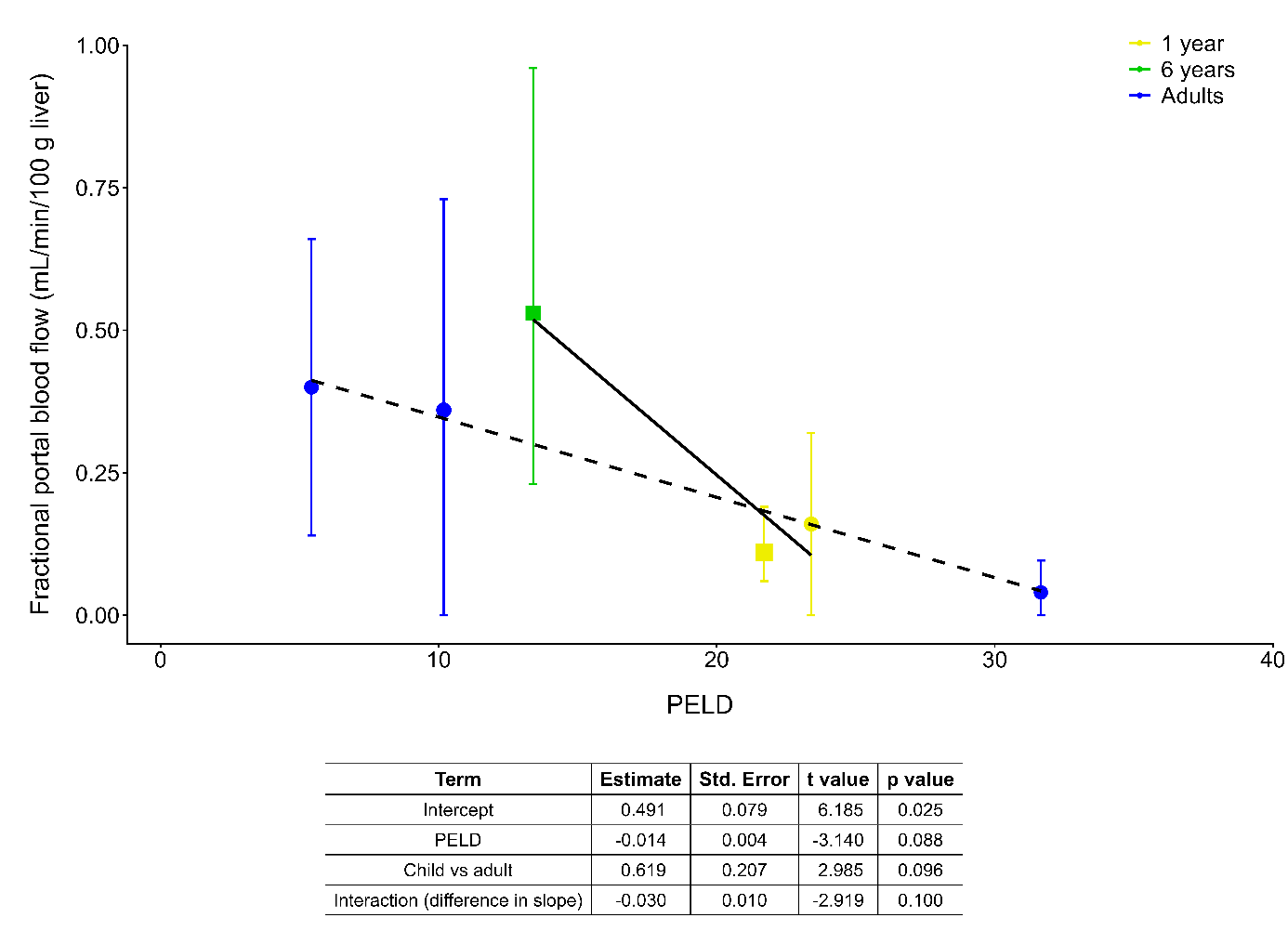


**Supplementary Figure S5** Interaction analysis of the relationship between PELD score and fractional hepatic arterial blood flow (mL/min/100 g liver) in children and adults.


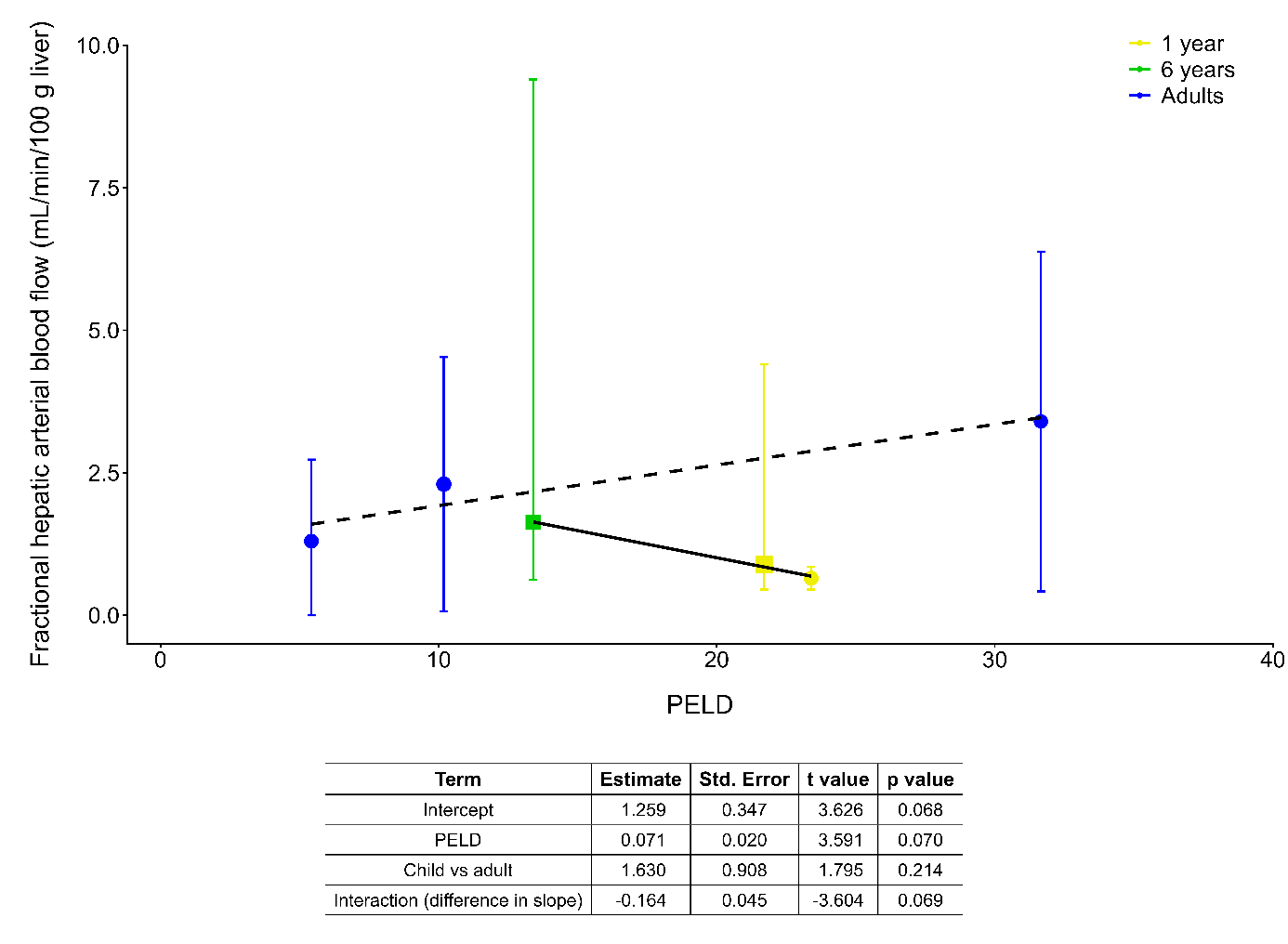


**Supplementary Figure S6** Interaction analysis of the relationship between Child-Pugh score and fractional renal blood flow (represented by the inverse of the fraction of renal resistive index) in children and adults.


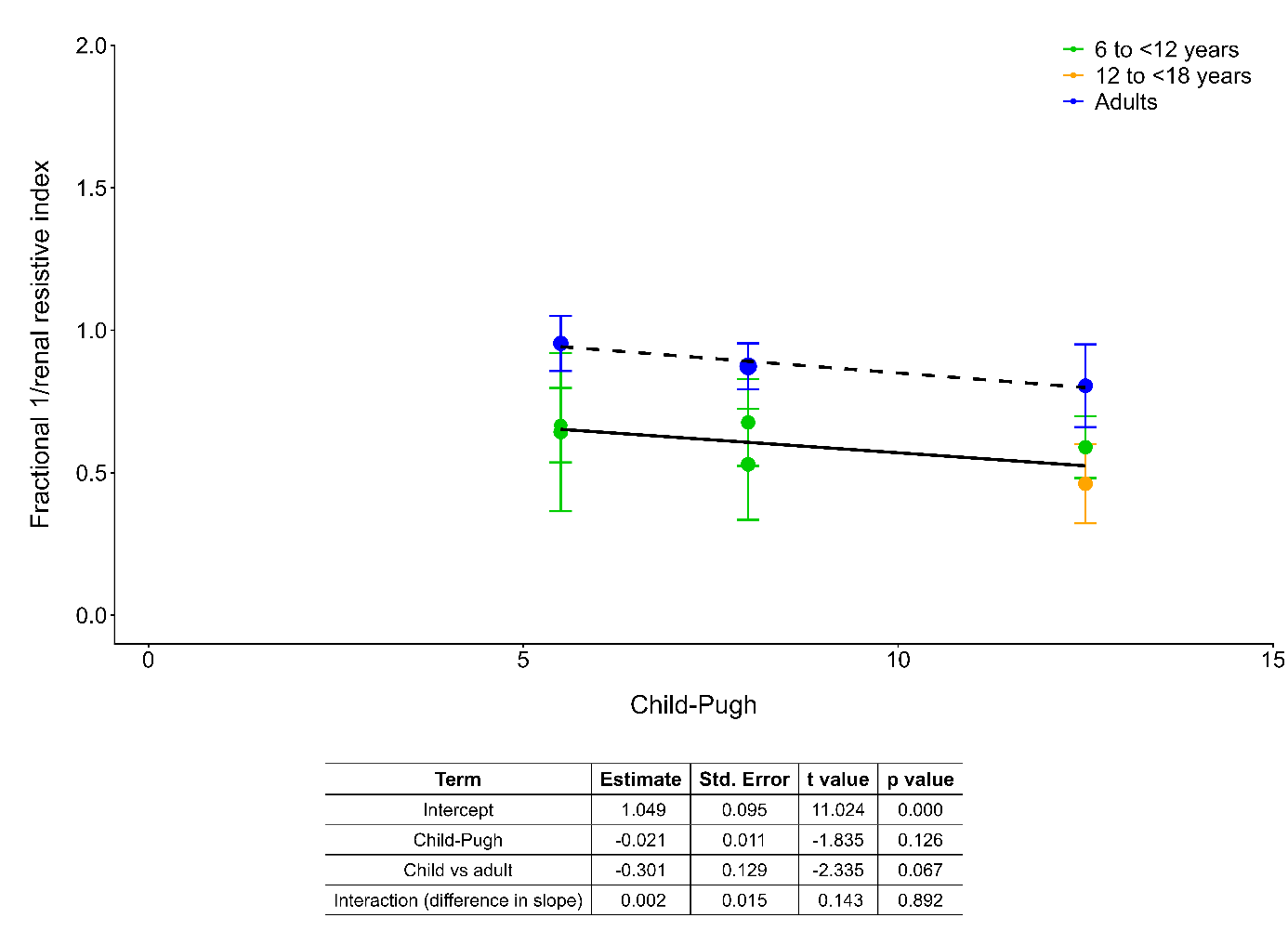


**Supplementary Figure S7** Interaction analysis of the relationship between PELD score and renal resistive index in children and adults.


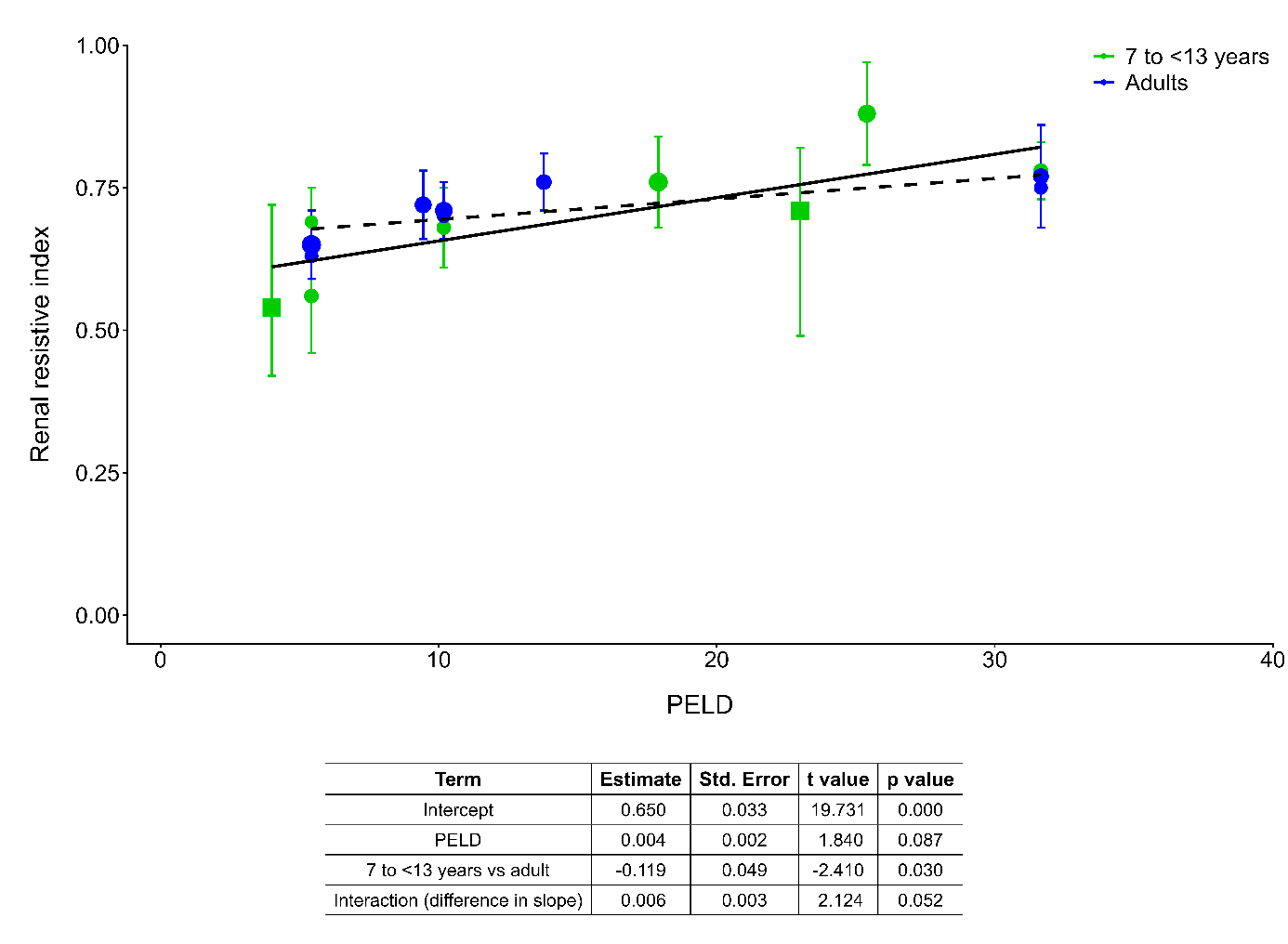


**Supplementary Figure S8** Interaction analysis of the relationship between PELD score and cardiac index (L/min/m^2^) in children and adults.


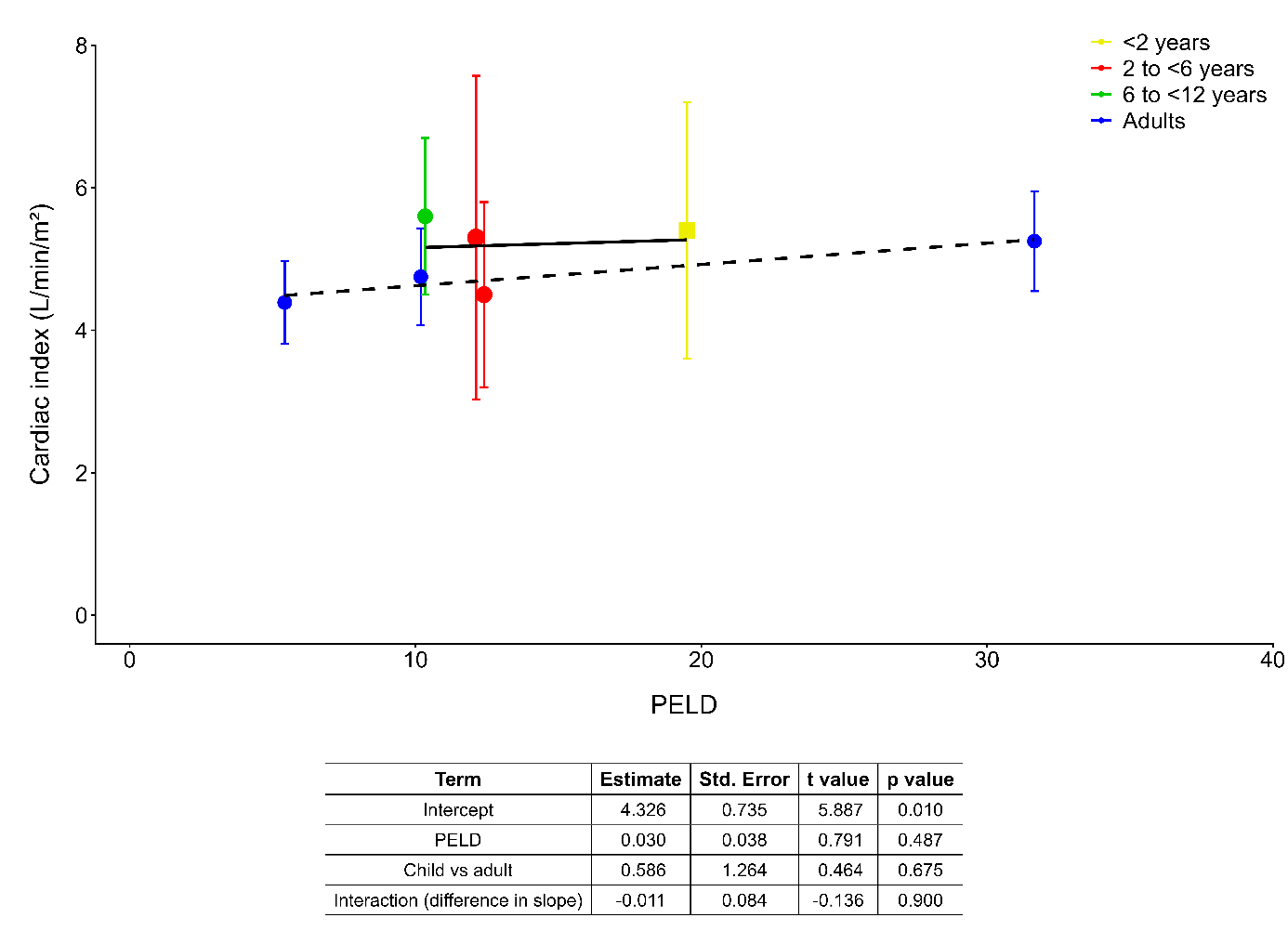


**Supplementary Figure S9** Interaction analysis of the relationship between PELD score and left ventricular ejection fraction (%) in children and adults.


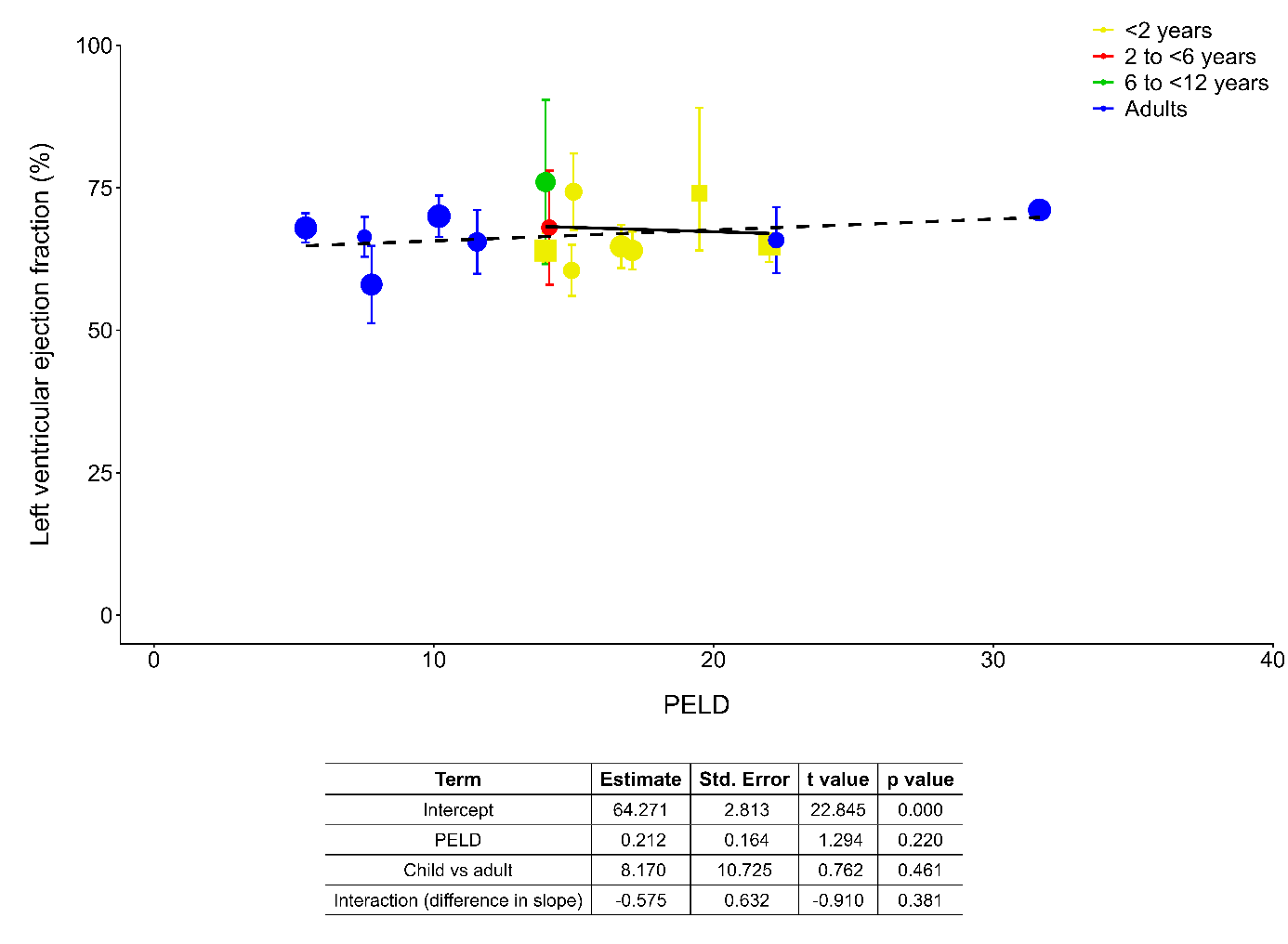

Supplement: Supplementary file 1 — Supplementary file1 (DOCX 547 KB) [file 11095_2026_4119_MOESM1_ESM.docx]
